# Genome-scale CRISPR activation screen uncovers tumor-intrinsic modulators of CD3 bispecific antibody efficacy

**DOI:** 10.1038/s41598-019-56670-x

**Published:** 2019-12-27

**Authors:** Corinne E. Decker, Tara Young, Elizabeth Pasnikowski, Joyce Chiu, Hang Song, Yi Wei, Gavin Thurston, Christopher Daly

**Affiliations:** Regeneron Pharmaceuticals, Inc., Tarrytown, NY 10591 United States

**Keywords:** Tumour immunology, Immunotherapy

## Abstract

Bispecific antibodies (bsAb) that bridge tumor cells and CD3-positive effector T cells are being developed against many tumor cell targets. While tumor cell factors other than target expression level appear to play a role in determining the efficacy of CD3 bsAb, the identity of such factors remains largely unknown. Using a co-culture system of primary human T cells and B lymphoma cell lines, we demonstrate a range of sensitivities to CD20xCD3 bsAb that is independent of CD20 surface expression. To identify genes that modulate tumor cell sensitivity to CD3 bsAb, we employed a genome-scale CRISPR activation screen in a CD20xCD3-sensitive human B lymphoma cell line. Among the most highly enriched sgRNAs were those targeting genes with predicted effects on cell-cell adhesion, including sialophorin (SPN). Increased expression of SPN impeded tumor cell clustering with T cells, thereby limiting CD3 bsAb-mediated tumor cell lysis. This inhibitory effect of SPN appeared to be dependent on sialylated core 2 O-glycosylation of the protein. While SPN is not endogenously expressed in the majority of B cell lymphomas, it is highly expressed in acute myeloid leukemia. CRISPR-mediated SPN knockout in AML cell lines facilitated T cell-tumor cell clustering and enhanced CD3 bsAb-mediated AML cell lysis. In sum, our data establish that the cell cross-linking mechanism of CD3 bsAb is susceptible to subversion by anti-adhesive molecules expressed on the tumor cell surface. Further evaluation of anti-adhesive pathways may provide novel biomarkers of clinical response and enable the development of effective combination regimens for this promising therapeutic class.

## Introduction

CD3 bispecific antibodies (CD3 bsAb) that simultaneously engage a tumor cell surface target and CD3 on T cells are emerging as promising therapeutics^1–5^. While the concept of bypassing MHC-restriction to cross-link any effector T cell to a tumor cell is straightforward in theory, in practice CD3 bsAb show variable efficacy in both hematological and solid malignancies^6,7^. Emerging preclinical and clinical data suggest that high expression of the target is not sufficient to drive tumor regression^8,9^.

A major unanswered question in the field of CD3 bsAb is the degree to which tumor-intrinsic factors, other than target expression, modulate T cell activation and cytotoxic effector function. *In vitro* studies using freshly-isolated healthy donor T cells stimulated with Blinatumomab, a CD19xCD3 bispecific T cell engager (BiTE) approved for pediatric B-ALL, demonstrated that tumor cell surface molecules other than CD19 modulate the magnitude of T cell activation, proliferation, and ultimately tumor cell killing^10^. While induction of PD-L1 on B-ALL target cells limited CD19xCD3-induced killing, CD80 up-regulation increased tumor cell sensitivity to CD19xCD3 *in vitro*. Importantly, these tumor factors showed the greatest effects at low effector-to-target ratios *in vitro* which may be more representative of physiological conditions *in vivo*. Similar studies of AMG330, a CD33xCD3 BiTE, showed that forced expression of PD-L1 on AML target cells decreased killing by freshly-isolated human T cells, while up-regulation of CD80 or CD86 potentiated T cell killing^11^.

The concept that tumor cell phenotype plays an active role in shaping CD3 bsAb efficacy also extends to clinical findings. In a retrospective study of CD19+ relapsed/refractory ALL patients treated with Blinatumomab, it was found that robust effector T cell expansion during treatment correlated with an overall better therapeutic response^8^. While myriad factors might influence the clinical outcome it is clear that CD19 expression alone is not sufficient to drive T cell expansion and effector function. Discovery of tumor factors that augment or limit the T cell response could lead to the development of novel therapeutic biomarkers and/or combination approaches. Identification of tumor-intrinsic factors distinct from known inhibitory or stimulatory T cell ligands would be of particular interest.

CD20xCD3 bsAb is in clinical trials for Non-Hodgkin’s B cell lymphoma and was previously described to induce tumor regression in xenogenic human B cell models^12^. Using an *in vitro* co-culture system of primary human T cells and B lymphoma cell lines, we demonstrate a range of sensitivities to CD20xCD3 bsAb that is independent of CD20 surface expression. Here we describe the implementation of an unbiased CRISPR activation screen to identify tumor-intrinsic factors that limit CD3 bsAb-mediated tumor cell killing.

## Results

### Tumor cell determinants, other than target expression level, modulate CD20xCD3-induced T cell activation and cytotoxicity *in vitro*

**Figure 1:**
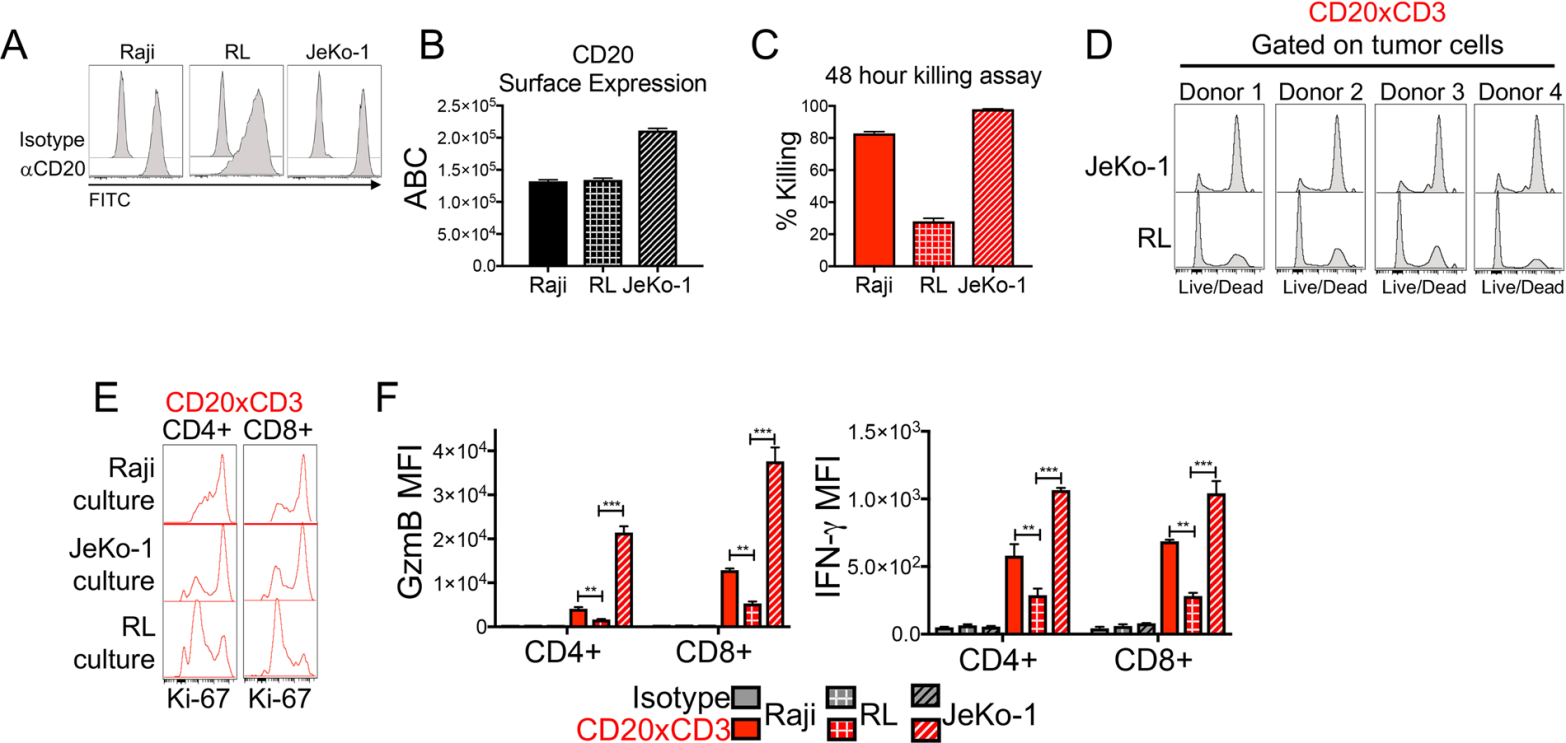
Tumor cell determinants other than target expression level modulate CD20xCD3-induced T cell activation and cytotoxicity. (**A**) Three human non-Hodgkin’s Lymphoma cell lines, Raji (Burkitt’s lymphoma), JeKo-1 (Mantle cell lymphoma) and RL (Diffuse large B cell lymphoma) were stained for the cell surface molecule CD20. (**B**) Raji, RL, and JeKo-1 cells were stained with BD Quantibrite anti-CD20 antibody. CD20 antigen density is expressed as antibodies bound per cell (ABC). (**C**) Raji, RL, and JeKo-1 cells were co-cultured with freshly-isolated healthy donor T cells at a 3:1 E:T ratio. Co-cultures were treated with 30 ng/ml CD20xCD3 bsAb for 48 hours, followed by staining for viable cells and FACS analysis. Percent killing was calculated by quantifying the number of live cells in CD20xCD3-treated wells compared to cells treated with a CD3-binding isotype control antibody. (**D**) T cells were isolated from four different healthy donors and co-cultured with JeKo-1 and RL tumor cells. After 48 hours of treatment with 30 ng/ml CD20xCD3 bsAb, cells were stained with viability dye and analyzed by FACS to assess tumor cell killing. (**E**) T cells were co-cultured with human tumor cell lines and 30 ng/ml CD20xCD3 bsAb or a CD3-binding isotype control antibody. After 48 hours, cells were collected and stained for the proliferation marker Ki-67. Representative histograms of gating to CD4+ and CD8 + T cell populations are shown. (**F**) T cells were co-cultured with human tumor cell lines and 30 ng/ml CD20xCD3 bsAb or a CD3-binding isotype control. After 48 hours, cells were collected and stained for T cell effector molecules Granzyme B (GzmB) and IFNγ. Average median fluorescence intensity (MFI) for CD4 + and CD8 + T cell populations are shown. **P < 0.01, ***P < 0.001 by two-tailed T-test.

To identify tumor-intrinsic factors that influence CD3 bsAb efficacy, we first sought to develop an *in vitro* human T cell-tumor cell co-culture system which would allow us to detect a range of tumor cell sensitivities to CD3 bsAb. Such a system could then be manipulated in screening approaches to identify tumor cell factors that modulate CD3 bsAb-mediated T cell killing. We compared the *in vitro* sensitivity of three human B cell lymphoma lines: Raji (Burkitt’s lymphoma), JeKo-1 (Mantle Cell Lymphoma), and RL (Diffuse Large B Cell Lymphoma). Each of these cell lines expresses high surface levels of the target CD20 (Fig. 1A). Quantification of CD20 antigen density using the QuantiBrite system revealed equivalent anti-CD20 binding capacity of Raji and RL cells, with JeKo-1 cells exhibiting moderately higher CD20 antigen density (Fig. 1B). To determine the sensitivity of these cell lines to CD3 bsAb, we co-cultured healthy donor T cells with each tumor cell line and CD20xCD3 bsAb for 48 hours. Both Raji and JeKo-1 tumor cells were sensitive to CD20xCD3 bsAb with 80–90% of tumor cells lysed by T cells (Fig. 1C). RL tumor cells, however, were strikingly less susceptible to CD20xCD3-mediated T cell killing *in vitro*. T cells isolated from four different healthy donors were able to kill the majority of JeKo-1 tumor cells within 48 hours of treatment, while RL tumor cells survived, confirming that this difference in sensitivity is not donor-dependent **(**Fig. 1D**)**. Thus, CD20xCD3 bsAb efficacy is modulated by tumor-intrinsic factors other than CD20 surface expression.

To determine if differential killing of tumor cells is associated with differences in CD20xCD3-induced T cell effector function, we assessed CD4+ and CD8+ T cell activation in co-culture with Raji, JeKo-1, and RL tumor cells. After 48 hours of culture with Raji or JeKo-1 tumor cells and CD20xCD3 bsAb, both CD4+ and CD8+ T cells were highly proliferative, as indicated by increased expression of Ki-67 **(**Fig. 1E**)**. Both CD4+ and CD8+ T cells cultured with Raji or JeKo-1 tumor cells exhibited increased expression of the effector molecules Granzyme B and IFNγ **(**Fig. 1F**)**. CD20xCD3-induced T cell activation was blunted in co-culture with RL tumor cells, as evidenced by weak CD4+ and CD8+ T cell proliferation and relatively modest induction of IFNγ and Granzyme B. These data suggest that tumor-intrinsic factors in addition to target expression level determine the magnitude of CD3 bsAb-induced T cell activation and ultimate cytotoxicity.

### Tumor-intrinsic factors underlie differential sensitivity to CD20xCD3-mediated killing

**Figure 2:**
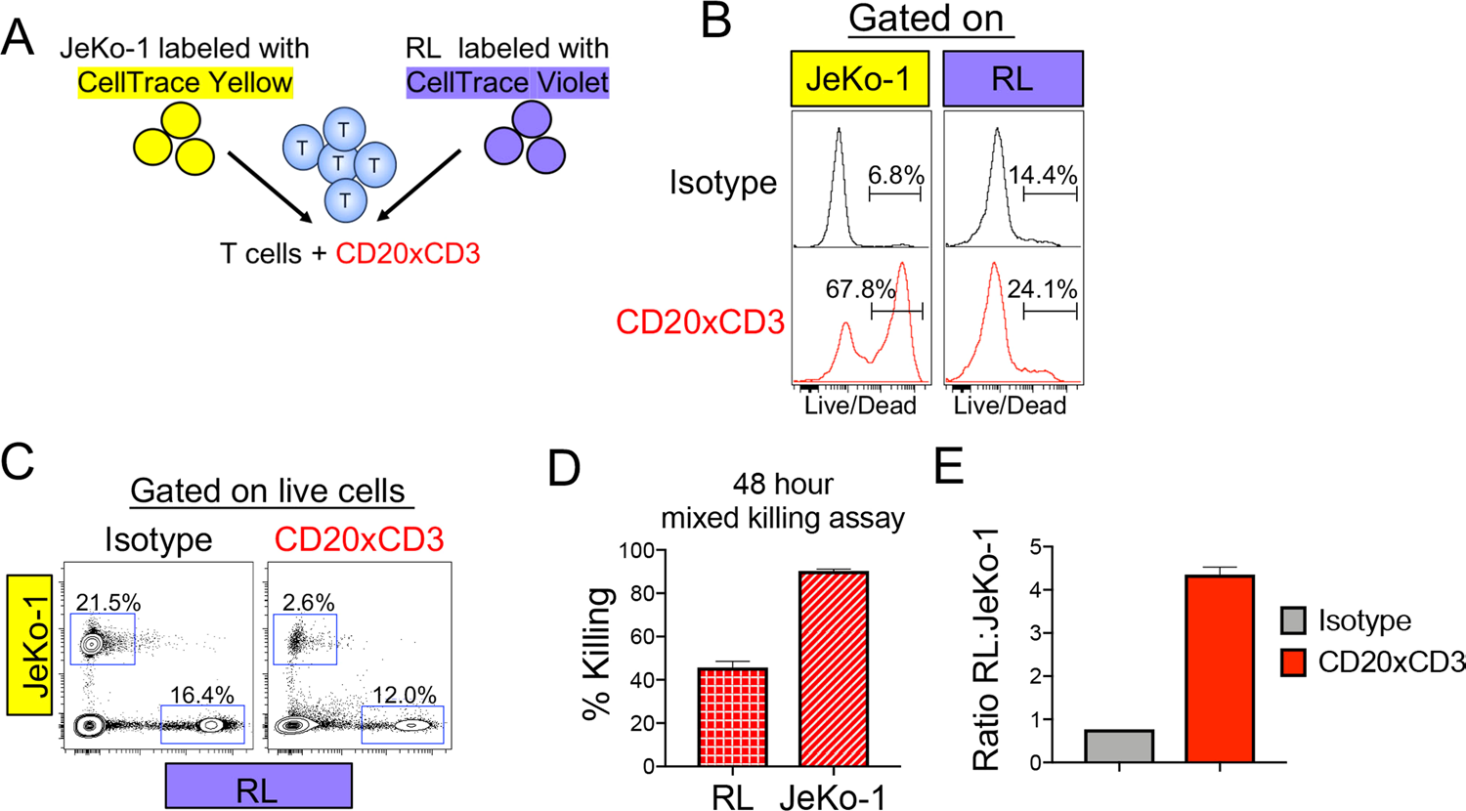
Tumor cell-intrinsic factors mediate differential sensitivity to CD20xCD3-induced T cell killing. (**A**) JeKo-1 tumor cells were labeled with CellTrace Yellow (CTY), and RL tumor cells were labeled with CellTrace Violet (CTV). Labeled tumor cells were mixed at a 1:1 ratio and co-cultured with healthy donor T cells (3:1 E:T) and 30 ng/ml CD20xCD3 bsAb or CD3-binding isotype control antibody for 48 hours, followed by staining with viability dye and FACS analysis. (**B**) Representative histograms show viability staining of JeKo-1 (CTY+) and RL (CTV+) cells after co-culture with T cells and CD20xCD3 bsAb or CD3-binding isotype control antibody. (**C**) Representative FACS plots show relative proportions of live JeKo-1 and RL tumor cells in co-culture with T cells and CD20xCD3 bsAb or CD3-binding isotype control antibody. (**D**) The percent killing for JeKo-1 and RL tumor cells in a mixed killing assay was calculated by quantifying the number of live cells remaining after CD20xCD3 bsAb treatment compared to CD3-binding isotype control antibody. (**E**) Decreased sensitivity of RL cells to T cell-mediated killing results in an increase in the ratio of live RL tumor cells to live JeKo-1 tumor cells after 48 hours of CD20xCD3 bsAb treatment.

Our preceding data suggest the possibility that weaker killing of RL cells reflects a reduced ability to support T cell activation. To directly compare the susceptibility of RL and Jeko-1 cells to T cell killing, we designed a mixed killing assay. JeKo-1 and RL tumor cells were differentially labeled with CellTrace dyes and mixed with healthy donor T cells (Fig. 2A). After 48 hours of CD20xCD3 bsAb treatment, T cells cultured with a mixture of JeKo-1 and RL tumor cells strongly up-regulated IFNγ and Granzyme B, indicating that the T cells were effectively activated (Supplemental Fig. 1). The majority of JeKo-1 cells remaining after 48 hours of treatment with CD20xCD3 bsAb showed compromised viability, evidenced by the uptake of membrane-permeable live/dead stain (Fig. 2B), concomitant with an ~80% reduction in JeKo-1 cells compared to cultures treated with isotype control (Fig. 2C,D). RL tumor cells were able to persist in this mixed culture, even in the presence of highly effective cytotoxic T cells, resulting in a 4-fold increase in the ratio of live RL cells to live JeKo-1 cells after 48 hours of treatment (Fig. 2E). Thus, independent of their ability to support CD3 bsAb-induced T cell activation, JeKo-1 and RL tumor cells are differentially sensitive to T cell killing, suggesting that tumor-intrinsic factors limit the killing mechanism of cytotoxic T cells.

It is increasingly well-understood that tumor cells can express a number of co-stimulatory and co-inhibitory molecules that potently modulate the magnitude of T cell activation and effector responses^13^. To measure the expression of such factors on Raji, JeKo-1, and RL tumor cells in a high-throughput manner, we utilized a LegendARRAY of ~360 antibodies. We found no striking differences in expression of known co-inhibitory or co-stimulatory molecules such as PD-L1, CD80/86, or 4-1BB that might explain the differential tumor cell sensitivity to CD20xCD3 bsAb (Supplemental Fig. 2).

### Genome-scale CRISPR activation screen identifies novel determinants of CD20xCD3-mediated T cell killing

To identify genes that can limit CD20xCD3-mediated tumor cell killing, we employed a genome-scale CRISPR activation screen in CD20xCD3-sensitive JeKo-1 tumor cells. This CRISPR/Cas9 system utilizes modified single-guide RNAs (sgRNAs) and a catalytically-inactive Cas9 (dCas9) to recruit a synergistic activation mediator (SAM) complex to gene promoters, resulting in robust target gene induction^14^. To validate this transcriptional activation system in engineered JeKo-1/dCas9/MS2 cells, we expressed sgRNAs targeting the granzyme B inhibitor SERPINB9 as well as sgRNAs targeting PD-L1. SERPINB9 protein expression was robustly induced by the SERPINB9 sgRNA compared to a control non-targeting sgRNA (Supplemental Fig. 3A). Similarly, PD-L1 total protein and surface levels were significantly increased by PD-L1-targeted sgRNAs (Supplemental Fig. 3B,C). Thus, this CRISPR SAM system effectively induces the expression of targeted genes in JeKo-1/dCas9/MS2 cells.

**Figure 3:**
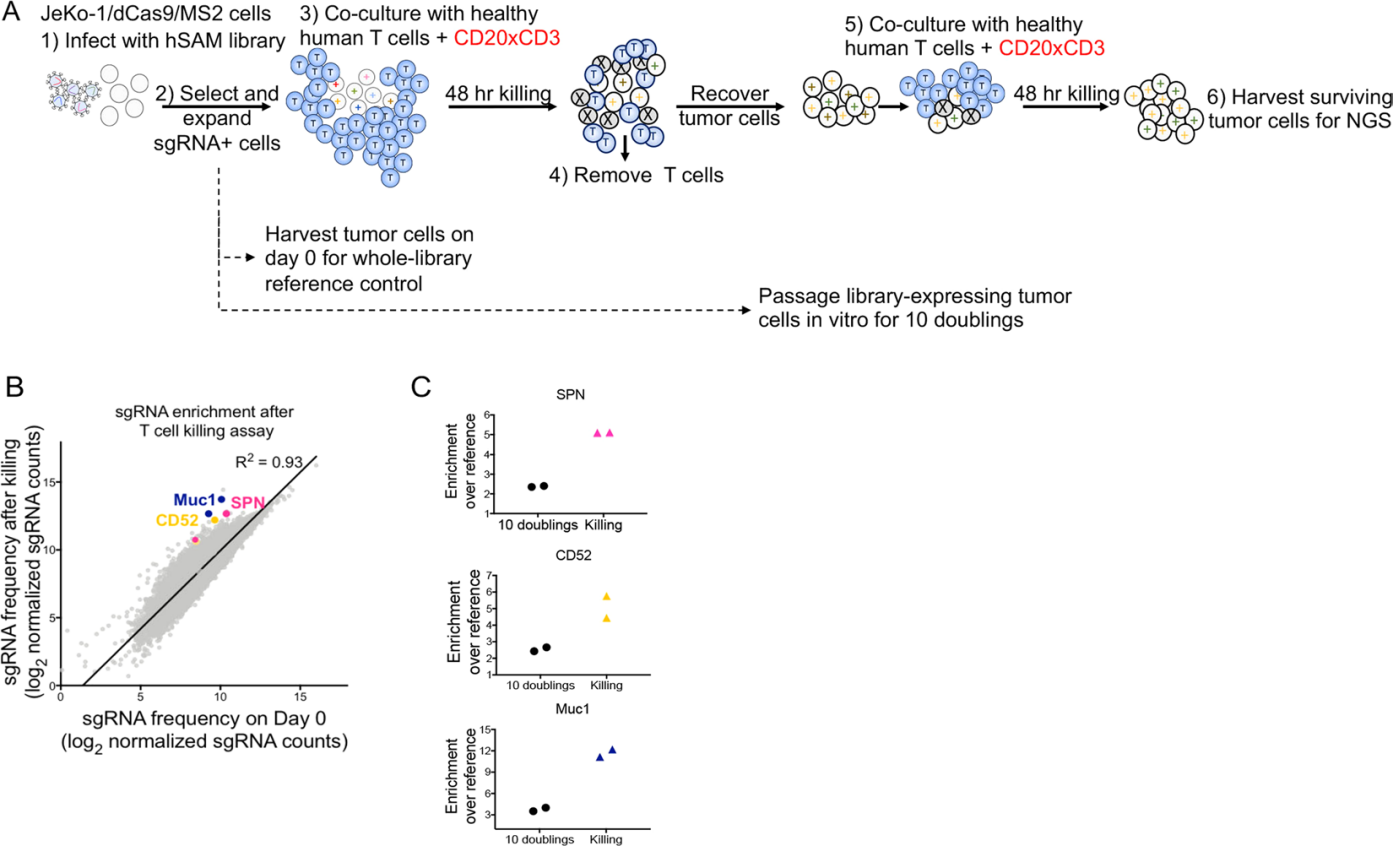
Genome-scale CRISPR transcriptional activation screen in Jeko-1 cells. (**A**) JeKo-1/dCas9/MS2 cells were infected with a human CRISPR SAM library of 70,290 sgRNAs. sgRNA-expressing cells were co-cultured with human T cells (3:1) E:T and 30 ng/ml CD20xCD3 bsAb. Triplicate killing assays were set up at 500x library representation. After an initial killing assay of 48 hours, T cells were removed by anti- CD3 positive selection, surviving tumor cells were expanded, and the killing assay was repeated with fresh T cells and CD20xCD3 bsAb. After 48 hours, surviving tumor cells were harvested and processed for Next-Generation Sequencing and comparison of sgRNA representation to that in reference control tumor cells harvested immediately after antibiotic selection. In parallel with T cell killing assays, library-modified JeKo-1/dCas9/MS2 cells were passaged *in vitro* and harvested after 10 doublings. (**B**) Comparison of normalized sgRNA counts in the tumor cell population collected after T cell killing compared to tumor cells harvested on day 0 before T cell killing. Normalized sgRNA counts were averaged across triplicate samples for each condition. 3 genes of interest (SPN, CD52, and MUC1), each with 2 top-scoring sgRNAs are highlighted. R^2^ value calculated by Pearson’s correlation. (**C**) Enrichment of 2 sgRNAs targeting SPN, CD52, or MUC1 in tumor cells passaged *in vitro* for 10 doublings and in tumor cells that survived CD20xCD3-mediated T cell killing.

JeKo-1/dCas9/MS2 tumor cells were transduced with a genome-scale pooled sgRNA library consisting of 70,290 sgRNAs targeting 23,430 genes (Fig. 3A). JeKo-1/dCas9/MS2 cells expressing library sgRNAs were cultured with freshly-isolated human T cells and CD20xCD3 bsAb. After 48 hours, T cells were removed by CD3 positive selection, and remaining tumor cells were expanded before a second round of CD20xCD3-mediated T cell killing to increase enrichment of resistant tumor cells. JeKo-1/SAM library cells were also passaged *in vitro* for 10 doublings to identify genes that affect tumor cell survival or growth independent of T cells and CD20xCD3 bsAb treatment.

We used Next-Generation Sequencing to quantify the representation of each sgRNA in the live tumor cell population harvested before and after CD20xCD3-mediated T cell killing (Fig. 3B). Importantly, sgRNA representation measured after 10 population doublings was not significantly shifted from the day 0 whole library reference control (Supplemental Fig. 4A), suggesting that changes in sgRNA frequency after T cell killing reflect a biological effect and not a technical artifact.

To discover genes that limit JeKo-1 sensitivity to CD20xCD3-mediated T cell killing, we first identified sgRNAs that were significantly (p < 0.05) enriched at least 1.5-fold in live tumor cells harvested after T cell killing compared to tumor cells harvested on day 0 (Supplemental Table 1). From this list, we identified 37 genes that were targeted with at least 2 distinct sgRNAs (Supplemental Table 2). Genes for which the average enrichment of the two sgRNAs that scored in the screen was at least 2-fold are listed in Table 1. Among these genes are known regulators of apoptosis including A20 and MCL1, a BCL2 family protein^15,16^. Many of the sgRNAs that were increased after T cell killing were also enriched, albeit to a lesser extent, after 10 population doublings, suggesting that the targeted gene may affect both general cell fitness and sensitivity to CD3 bsAb (Supplemental Table 3).

**Table 1:** Genes with an average enrichment of 2 sgRNAs ≥ 2-fold over Day 0 Reference, p < 0.05 by two-tailed T-test.

Gene	Average enrichment
SPN (CD43)	5.1
CD52	5.1
GLIS3	5.0
GSTK1	4.0
KSR2	3.5
ZP3	2.8
ATP8B1	2.6
PROX1	2.5
NR4A1	2.4
MCL1	2.3
ATP8B2	2.3
A20	2.2
HLA-DPA1	2.1
B4GALNT1	2.1
B3GNT4	2.0

We noticed that several of the most highly-enriched sgRNAs target genes that encode cell surface proteins with purported effects on cell-cell adhesion including sialophorin (SPN), CD52, and MUC1. Two sgRNAs targeting each of these genes were enriched at least 4-fold in the live tumor cell population harvested after T cell killing (Fig. 3C). The fact that these are cell surface proteins is consistent with the possibility that they might exert a direct effect on the CD3 bsAb mechanism of action (physical cross-linking of effector T cells to tumor cells). In addition, SPN and CD52 are both endogenously expressed in the B cell lineage and thus physiologically relevant to our CD3 bsAb of interest, CD20xCD3^17–19^. While MUC1 is normally expressed in epithelial cells, where its highly glycosylated extracellular domain acts as a protective barrier^20^, we decided to include MUC1 in our screen validation work due to the possible functional similarity between MUC1 and SPN (both proteins carry extensive O-linked glycan structures).

To evaluate on-target activity for these sgRNAs, we expressed each sgRNA in JeKo-1/dCas9/MS2 cells and assessed protein induction by FACS. Control non-targeted Jeko-1 cells express both SPN and CD52 at baseline but do not express MUC1. For each gene, two of the three sgRNAs in the library substantially increased protein expression, and these corresponded to the sgRNAs that were significantly enriched in the screen (Supplemental Fig. 4B–D). For each gene, there was one sgRNA in the library that was not enriched in the screen; these sgRNAs were the least effective at inducing target gene expression. Thus, sgRNA enrichment in the screen is associated with robust on-target gene induction.

### SPN, CD52, and MUC1 induction in tumor cells limits CD20xCD3-mediated T cell killing

**Figure 4:**
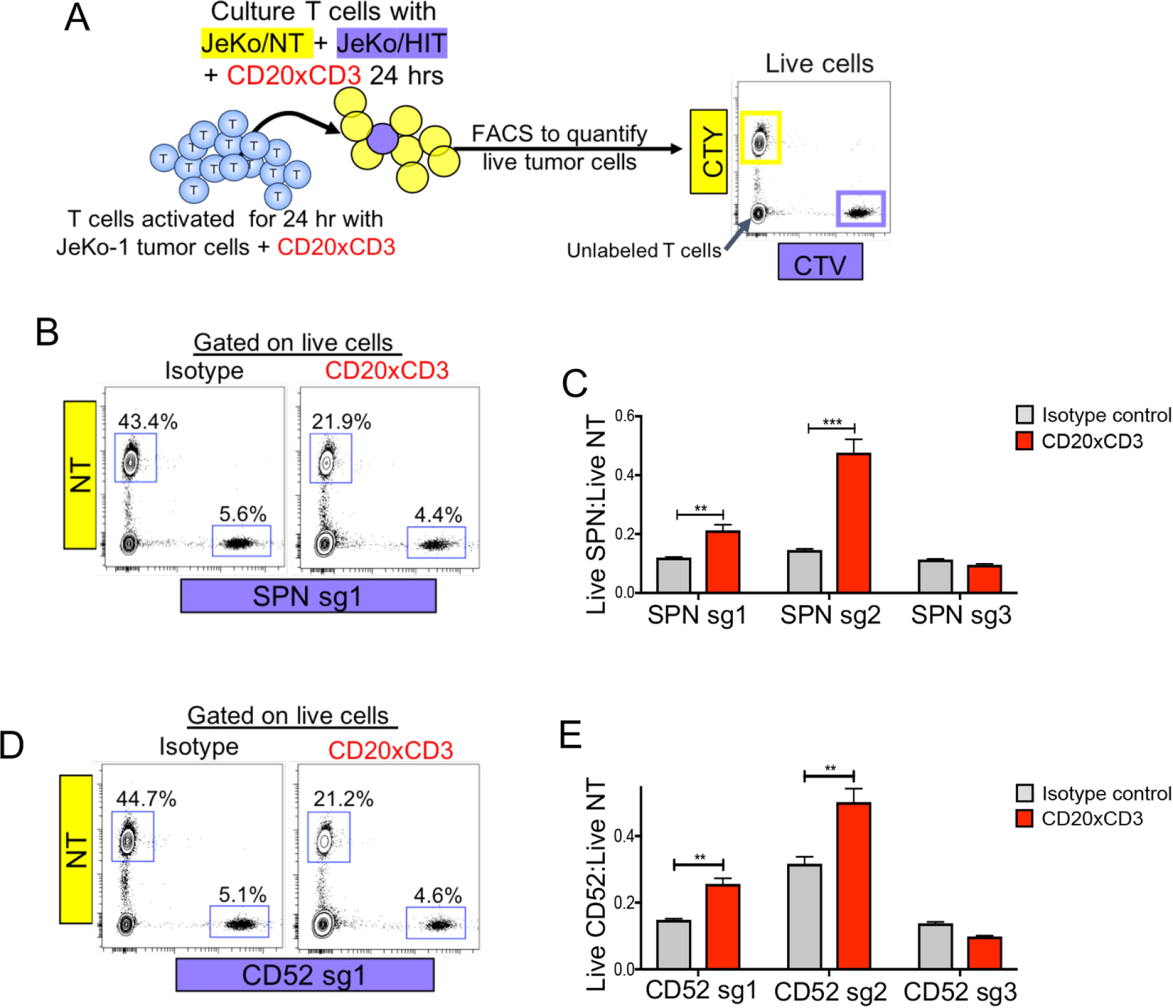
SPN and CD52 modulate CD20xCD3 bsAb efficacy *in vitro*. (**A**) JeKo-1/dCas9/MS2 cells expressing a non-targeting (NT) control sgRNA or a SPN/CD52-targeted sgRNA were differentially labeled with CellTrace Yellow (CTY) or CellTrace Violet (CTV). Differentially labeled cells were mixed at a ratio of 8:1 control:targeted cells and co-cultured with pre-activated T cells and 30 ng/ml CD20xCD3 bsAb or CD3-binding isotype control antibody. After 24 hours, cells were stained with viability dye and analyzed by FACS. (**B**) Representative FACS plots show the proportions of live JeKo-1/NT and JeKo-1/SPN cells following treatment. (**C**) The ratio of live JeKo-1/SPN cells to JeKo-1/NT cells was calculated following treatment. JeKo-1 cells expressing SPN sg1 or sg2 (which effectively induce SPN expression and were enriched in the screen) were less sensitive to T cell killing, while the less effective SPN sg3 did not confer protection from killing. **P < 0.01, *** P < 0.001 by two-tailed T-test. (**D**) Representative FACS plots show the proportions of live JeKo-1/NT and JeKo-1/CD52 cells following treatment. (**E**) The ratio of live JeKo-1/CD52 cells to JeKo-1/NT cells was calculated following treatment. JeKo-1 cells expressing CD52 sg1 or sg2 (which effectively induce CD52 expression and were enriched in the screen) were less sensitive to T cell killing, while the less effective CD52 sg3 did not confer protection from killing. **P < 0.01 by two-tailed T-test.

We next evaluated whether sgRNA-mediated overexpression of the candidate genes (SPN, CD52, MUC1) could protect JeKo-1 cells in a competition killing assay (Fig. 4A). JeKo-1/dCas9/MS2 cells expressing a targeted sgRNA were mixed at a low ratio with JeKo-1/Non-targeting (NT) cells. The killing assays were conducted for 24 hours, a time frame in which control and targeted Jeko-1 cells exhibited no differences in growth rate (Supplemental Fig. 4E). Activated T cells were co-cultured with dye-labeled NT and targeted JeKo-1 cells and CD20xCD3 bsAb. In isotype control-treated conditions, live JeKo-1/NT cells outnumbered targeted cells 8-to-1 (Fig. 4B,D). JeKo-1/SPN and JeKo-1/CD52 cells were less sensitive to T cell killing compared to non-targeted cells, as evidenced by a significant increase in the ratio of live targeted cells to NT cells (Fig. 4C,E). The sgRNAs that were not enriched in the screen (SPN sg3 and CD52 sg3) did not confer any survival advantage. Further validating our screen results, JeKo-1/MUC1 cells were less sensitive to CD20xCD3-mediated killing compared to NT cells (Supplemental Fig. 5A,B). These data substantiate the screen results that expression of SPN, CD52 or MUC1 on tumor cells limits CD20xCD3-mediated killing.

Reduced sensitivity to CD20xCD3 bsAb could result from reduced surface expression of the target CD20. While induction of SPN or MUC1 expression did not affect anti-CD20 binding compared to control JeKo-1/NT cells, JeKo-1/CD52 exhibited a 2-fold reduction in anti-CD20 binding capacity (Supplemental Fig. 6A). Total CD20 protein levels were unchanged in JeKo-1/CD52 cells, however, suggesting that CD52 upregulation affects localization of CD20 on the cell surface (Supplemental Fig. 6B). Altogether, these data indicate that SPN and MUC1 modulate tumor cell sensitivity to CD20xCD3 bsAb independent of CD20 expression, while increased expression of CD52 directly affects CD20 cell surface levels.

### SPN and MUC1 impede T cell-tumor cell clustering

**Figure 5:**
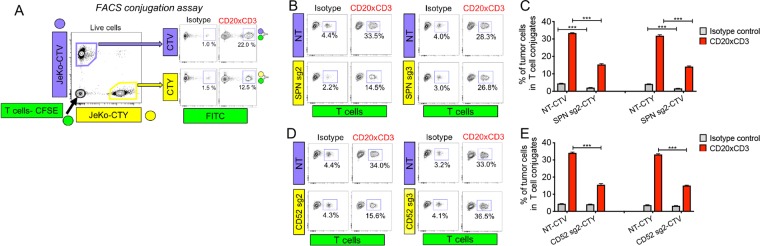
SPN limits tumor cell clustering with T cells. (**A**) To detect T cell-tumor cell clusters, healthy donor T cells were labeled with CellTrace CFSE and tumor cells were labeled with CellTrace Violet (CTV) or CellTrace Yellow (CTY). Differentially labeled tumor cells were mixed at a 1:1 ratio and co-cultured with labeled T cells (3:1 E:T) + 30 ng/ml CD20xCD3 bsAb or CD3-binding isotype control antibody. After 18 hours, cells were collected, fixed in 4% paraformaldehyde and immediately analyzed by FACS. (**A**) Representative plots showing the detection of T cell-tumor cell clusters. JeKo-1/NT cells in T cell conjugates are CTV + FITC +, and JeKo-1/SPN cells in T cell conjugates are CTY + FITC+ . (**C**) The average percent of tumor cells in conjugates with T cells was quantified across triplicate samples. Note that the extent of clustering was not affected by CellTrace dyes. ***P < 0.001 by two-tailed T-test. (**D**) Representative plots showing the detection of T cell-tumor cell clusters. JeKo-1/NT cells in T cell conjugates are CTV + FITC +, and JeKo-1/CD52 cells in T cell conjugates are CTY + FITC +. (**E**) The average percent of tumor cells in conjugates with T cells was quantified across triplicate samples. Note that the extent of clustering was not affected by CellTrace dyes. ***P < 0.001 by two-tailed T-test.

Since both SPN and MUC1 have been reported to limit T cell contacts with tumor cells *in vitro*^21–23^, we hypothesized that tumor cells overexpressing these proteins may be less sensitive to T cell killing as a result of decreased clustering with T cells. We adopted a FACS-based cell conjugation assay in which tumor cells and T cells are labeled with distinct CellTrace dyes. Control JeKo-1/NT cells and targeted JeKo-1 cells were mixed with healthy donor T cells and CD20xCD3 bsAb followed by FACS analysis to detect the presence of dual-fluorescing tumor cell-T cell clusters (Fig. 5A). In CD20xCD3 bsAb-treated conditions, ~30% of JeKo-1/NT cells were present in clusters with T cells (Fig. 5B,D). Upregulation of SPN or CD52 on tumor cells reduced T cell contacts by approximately half, while ineffective sgRNAs for each gene (SPN sg3 and CD52 sg3) had no effect on clustering. Consistent with its physiological role as an epithelial barrier protein, MUC1 induction on JeKo-1 cells similarly limited T cell contacts compared to JeKo-1/NT controls (Supplemental Fig. 5C,D). Reduced clustering was not an artifact of dye labeling, as JeKo-1/SPN, JeKo-1/CD52, or JeKo-1/MUC1 cells labeled with either CellTrace Violet (CTV) or CellTrace Yellow (CTY) dyes showed a significant reduction in T cell clustering (Fig. 5C,E; Supplemental Fig. 5D). Consistent with our observation that CD52 reduces CD20 surface levels, upregulation of CD52 affected only CD20xCD3-mediated T cell clustering. JeKo-1/SPN and JeKo-1/MUC1 cells, formed approximately half the number of T cell clusters as JeKo-1/NT cells even in isotype control conditions, indicating that SPN and MUC1 repel spontaneous tumor cell/T cell contacts that occur in co-culture conditions. These data support the hypothesis that SPN and MUC1 on tumor cells can drive T cell evasion by an anti-adhesive mechanism.

### SPN expression and glycosylation varies across human NHL and AML cell lines

**Figure 6:**
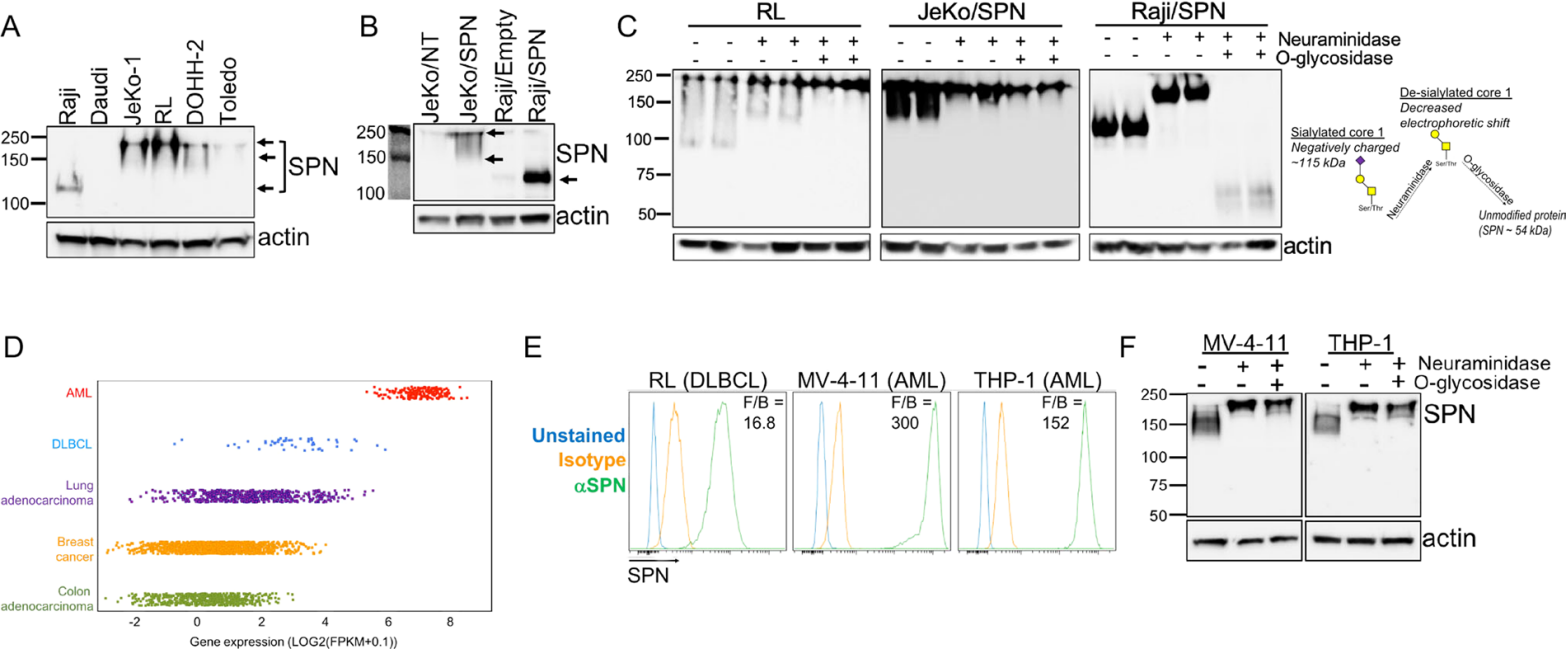
SPN expression and glycosylation varies across human NHL and AML cell lines. (**A**) Total cell lysates from six human Non-Hodgkin’s lymphoma cell lines were separated by SDS-PAGE and subjected to Western blot analysis for SPN detection. Arrows indicate distinct SPN bands in different cell lines. Uncropped blots shown in Figure S9A. (**B**) Total cell lysates from JeKo-1 and Raji control cells or cells expressing ectopic SPN were separated by SDS-PAGE and subjected to Western blot analysis for SPN detection. Uncropped blots shown in Figure S8B. (**C**) RL, JeKo-1/SPN and Raji/SPN cells were untreated, treated with 1 U/ml neuraminidase alone to remove sialic acids, or neuraminidase in combination with 0.2 U/ml O-glycosidase to remove sialic acids and core 1 O-glycans. Total cell lysates were separated by SDS-PAGE and subjected to Western blot analysis for SPN detection. Uncropped blots shown in Figure S9C. (**D**) Comparison of SPN transcript expression across acute myeloid leukemia (AML), Diffuse Large B cell Lymphoma (DLBCL), lung adenocarcinoma, breast cancer, and colon adenocarcinoma samples in The Cancer Genome Atlas (TCGA) database. (**E**) Human tumor cell lines RL (DLBCL), MV-4-11 (AML), and THP-1 (AML) were stained for surface expression of SPN. (**F**) The sialic acid and O-glycan content of SPN in MV-4-11 and THP-1 cells was assessed following treatment with neuraminidase, to remove sialic acids, and O-glycosidase, to cleave core 1 O-glycans. Total cell lysates were separated by SDS-PAGE and subjected to Western blot analysis for SPN detection. Uncropped blots shown in Figure S9D.

To investigate more broadly the potential therapeutic significance of our screen results in hematological malignancy, we focused on SPN. SPN is tightly regulated during B cell development with expression limited to the pro- and pre-B cell stages; SPN is not expressed by mature resting B cells^24,25^. Similarly, SPN is detected in a minority of Non-Hodgkin’s Lymphoma (NHL), especially in the two most common subtypes: follicular lymphoma (FL) and diffuse large B cell lymphoma (DLBCL). By immunohistochemistry, SPN protein is detected in less than 10% of FL biopsies and approximately 25% of DLBCL samples^17,18,26^. With these data in mind, we examined SPN levels in NHL cell lines and found that both the expression level and electrophoretic mobility of SPN was variable (Fig. 6A). Raji cells, which are relatively sensitive to T cell killing (Fig. 1), express low levels of SPN. To assess whether SPN can modulate T cell killing in a cell line other than Jeko-1, we generated a SPN-overexpressing Raji line. While transduced Raji cells exhibited high expression of SPN, the protein showed increased electrophoretic mobility compared to SPN in JeKo-1/SPN cells (Fig. 6B), which could be the result of differential glycosylation. The extracellular domain of SPN extends 45 nm from the cell surface and is post-translationally modified by ~80 O-glycans capped with negatively-charged sialic acid residues^27,28^. These structures are dynamic and can exist as core 1 O-glycans or larger, more highly-branched core 2 O-glycans^29,30^. The addition of core 2 O-glycans to SPN has been reported to be important for its anti-adhesive function in T cells^29,31,32^.

We used an enzymatic approach to characterize SPN glycosylation in RL, JeKo-1, and Raji cells. Cells were treated with neuraminidase to remove negatively-charged sialic acid residues and O-glycosidase to remove core-1 O-glycans; O-glycosidase cannot cleave when core 2 O-glycans are present. In untreated RL and JeKo-1 cells, SPN was detected as multiple bands ranging from ~130–250 kDa, consistent with previous reports of extensive glycosylation (Fig. 6C). SPN in transduced Raji cells was detected as a single band ~115 kDa, suggesting modification by sialylated core 1 O-glycans^27^. Neuraminidase treatment resulted in decreased electrophoretic mobility of SPN in each cell line, consistent with the removal of negatively-charged sialic acid residues. After O-glycosidase treatment, SPN in Raji cells was detected as ~55 kDa band, indicating that all core 1 O-glycans were cleaved and only the unmodified protein remained. O-glycosidase treatment did not affect SPN in RL or JeKo-1 cells, however, suggesting that SPN is modified by core 2 O-glycans in these cell lines. Consistent with these findings, SPN overexpression in Raji cells had only modest effects on T cell clustering and cytotoxity (data not shown), presumably due to the lack of SPN modification by core 2 O-glycans in this cell line.

Given that RL cells express SPN (with core 2 O-glycans) at somewhat higher levels than Jeko-1 cells, we asked whether knockout of SPN in RL cells would sensitize them to T cell killing (Supplemental Fig. 7A,B). As shown in Supplemental Fig. 7C, knockout of SPN had only a very minor sensitizing effect on RL cells. Thus, mechanisms other than differential SPN expression clearly contribute to the relative insensitivity of RL cells to CD3 bsAb mediated killing.

### SPN on AML cells limits CD3 bsAb efficacy *in vitro*

**Figure 7:**
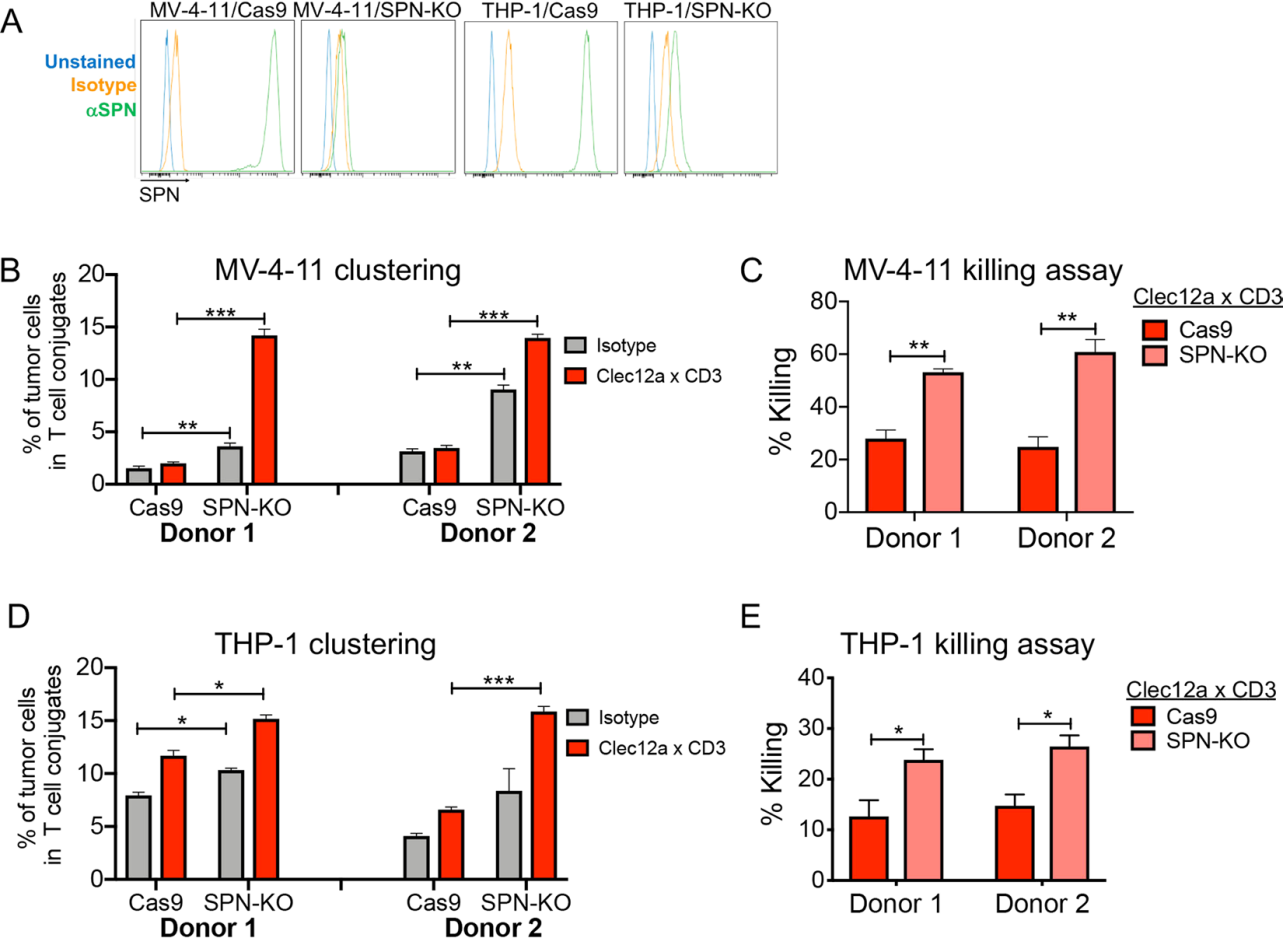
SPN knockout in AML cell lines potentiates clustering with T cells and tumor cell killing. (**A**) Plasmids encoding Cas9 alone or Cas9 plus an SPN-targeted KO sgRNA were packaged in lentivirus and transduced into MV-4-11 and THP-1 cells. Staining of cells expressing SPN KO sgRNA demonstrates almost complete elimination of SPN cell surface expression compared to Cas9 control cells. (**B**) Healthy donor T cells were labeled with CellTrace CFSE and MV-4-11 cells were labeled with CellTrace Violet (CTV). Labeled T cells and tumor cells were mixed at a 3:1 E:T and incubated with 15 ng/ml Clec12a x CD3 or CD3-binding isotype control antibody. After 8 hours of co-culture, cells were collected, fixed in 4% paraformaldehyde, and immediately analyzed by FACS. Significantly enhanced clustering of MV-4-11/SPN knockout cells was observed with two different T cell donors. **P < 0.01, ***P < 0.001 by two-tailed T-test. (**c**) MV-4-11 cells were loaded with Cell Trace Violet, mixed with pre-activated T cells and treated with 15 ng/ml Clec12a x CD3 bsAb or CD3-binding isotype control antibody. After 24 hours, cells were stained with viability dye and analyzed by FACS. Significantly increased sensitivity of MV-4-11/SPN KO cells to T cell killing was observed with two different T cells donors. (Donor 1 = 1:1 E:T, Donor 2 = 0.5:1 E:T) **P < 0.01 by two-tailed T-test. (**D**) Healthy donor T cells were labeled with CellTrace CFSE and THP-1 cells were labeled with CellTrace Violet (CTV). Labeled T cells and tumor cells were mixed (3:1 E:T) and incubated with 15 ng/ml Clec12a × CD3 or CD3-binding isotype control antibody. After 4 hours of co-culture, cells were collected, fixed in 4% paraformaldehyde, and immediately analyzed by FACS. Significantly enhanced clustering of THP-1/SPN knockout cells was observed with 2 different T cell donors. * P < 0.05, *** P < 0.001 by two-tailed T-test. (**E**) THP-1 cells were loaded with Cell Trace Violet, mixed with pre-activated T cells and treated with 15 ng/ml Clec12a x CD3 bsAb or CD3-binding isotype control antibody. After 24 hours, cells were stained with viability dye and analyzed by FACS. Significantly increased sensitivity of THP-1/SPN KO cells to T cell killing was observed with two different T cells donors. (Donor 1 = 1:1 E:T, Donor 2 = 0.5:1 E:T) ** P < 0.05 by two-tailed T-test.

Because SPN expression in NHL is rare, we examined SPN levels in additional malignances using The Cancer Genome Atlas (TCGA) database. We noted that SPN transcript is uniformly highly expressed in acute myeloid leukemia (AML) (Fig. 6D). We measured SPN protein expression in 2 human AML cell lines, MV-4-11 and THP-1, and detected cell surface levels 10 to 20-fold higher than those in RL cells (Fig. 6E). Moreover, neuraminidase and O-glycosidase treatment of MV-4–11 and THP-1 cells suggest that SPN is modified by sialylated core 2 O-glycans (Fig. 6F). Because this characterization shows that SPN is highly expressed in AML cells in a glycoform that is potentially anti-adhesive, we used CRISPR/Cas9 to generate SPN knockout cells (Fig. 7A, Supplemental Fig. 8A). To test the effect of SPN deletion on CD3 bsAb efficacy, we utilized a Clec12a x CD3 bsAb, as Clec12a is highly expressed on AML cells^33^. SPN deletion in MV-4-11 cells and THP-1 cells significantly increased clustering with healthy T cells (Fig. 7B,D**;** Supplemental Fig. 8B,C), even in isotype control conditions, indicating increased spontaneous clustering. In line with increased T cell contacts, SPN deletion increased AML cell sensitivity to Clec12a x CD3-mediated T cell killing (Fig. 7C,E). SPN deletion alone did not affect MV-4-11 or THP-1 survival in the absence of T cells (Supplemental Fig. 8D,E). These data support the notion that SPN on tumor cells imparts a physical barrier to effector T cells, thereby limiting the efficacy of CD3 bsAb.

## Discussion

Using a genome-scale CRISPR activation screen in human B lymphoma cells, we demonstrated that tumor-intrinsic factors other than target expression can modulate CD3 bsAb-mediated tumor cell killing. In particular, our findings uncover a role for highly-glycosylated cell surface molecules (SPN and MUC1) in hindering CD3 bsAb-induced T cell-tumor cell clustering and consequent tumor cell lysis. To extend our study beyond the B cell lineage, we focused on SPN in AML, where it is inherently highly expressed in an anti-adhesive glycoform. CRISPR-mediated knockout of SPN in AML cell lines significantly enhanced tumor cell-T cell clustering and consequent tumor cell lysis. Thus, our findings suggest that SPN may play a protective role in hematological cancers (particularly AML), promoting evasion of CD3 bsAb-mediated T cell killing. Similarly, MUC1 anti-adhesive function might limit the sensitivity of epithelial cell-derived solid tumors to T cell killing. While CD3 bsAb appear to be less effective when countered by anti-adhesive tumor cell surface proteins, our data do not indicate that these proteins provide complete protection from cytotoxic T cells. Rather, SPN and MUC1 modulate the degree of tumor cell sensitivity.

Our data are consistent with previous findings that SPN expression on tumor cells can limit tumor cell-T cell adhesion and T cell-mediated killing^21,23,34^. However, our work significantly extends prior studies by demonstrating the ability of SPN to limit tumor cell-T cell clustering even when cell-cell adhesion is actively promoted by a CD3 bsAb. It is important to note that SPN is natively expressed in leukocytes, including T cells, where it also appears to limit cell-cell adhesion^35–37^. Interestingly, T cell-expressed SPN is actively excluded from immune synapses^38^, consistent with the possibility that SPN functions to limit the formation of stable contacts between T cells and target cells. The anti-adhesive effect of T cell SPN might be particularly strong when the target cell (in our case a tumor cell) also expresses this large, negatively-charged molecule. Importantly, our data suggest that the ability of tumor cell SPN to limit T cell killing is dependent on its modification with core 2 O-glycans, which is consistent with previous findings on the anti-adhesive functions of both SPN and MUC1^31,32,39,40^.

Our data suggest the possibility that inhibition of SPN function might sensitize cancers to treatment with CD3 bsAb (as well as to other immunotherapies, since SPN significantly affects the ability of tumor cells to cluster with T cells even in the absence of CD3 bsAb). In particular, the uniform and high-level expression of SPN in AML (in contrast to the highly variable expression in NHL) suggests that it may have a clinically-relevant role in this indication. One potential concern with targeting SPN, however, is that it may have important functions in leukocytes via interactions with ligands such as E-selectin or Galectin-1^41–46^. Recently, Gillisen *et al*. identified a SPN-specific antibody derived from the B cell repertoire of a long-term AML survivor^47^. This antibody targeted a sialylated epitope on SPN that was expressed across all AML subtypes, but was not found on healthy monocytes, granulocytes, B cells or T cells. Targeting this unique SPN epitope in a CD3 bsAb format showed anti-tumor efficacy against human AML cell lines *in vitro* and *in vivo* while engrafted healthy myeloid cells were unaffected^48^. While discovery and generation of glycoform-specific therapeutics is arduous, it could be an effective way to preferentially inhibit SPN function on tumor cells. However, even if tumor-selective blockade of SPN function proves to be impractical (or not highly effective), SPN expression level might be a useful biomarker for sensitivity to CD3 bsAb therapy.

In summary, we have used an unbiased genetic screening approach to identify tumor cell factors that modulate responsiveness to CD3 bsAb. In addition to the specific mechanistic insights into CD3 bsAb-mediated cytotoxicity and SPN function that our work provides, our study suggests more broadly that CRISPR-based activation or knockout screens are an attractive way to elucidate the mechanisms that regulate tumor cell killing by T cells.

## Methods

See Supplemental Methods for details.

### Human tumor cell lines

Raji (Burkitt’s lymphoma), Daudi (Burkitt’s lymphoma), JeKo-1 (mantle cell lymphoma), RL (DLBCL), DOHH-2 (DLBCL), Toledo (DLBCL), MV-4-11 (AML), and THP-1 (AML) cell lines were obtained from ATCC, authenticated by Regeneron, and tested negative for Mycoplasma. All experiments were conducted with low-passage (1-6) cell cultures.

### Generation of CD3 bsAb

CD3 bsAb were generated using VelocImmune® mice as previously described^12,49,50^. Anti-human CD3, CD20, and Clec12a antibodies were produced with a human IgG4 Fc harboring substitutions that minimize Fc-dependent effector functions. To facilitate bsAb purification, two point mutations were introduced into the CD3 heavy chain to limit protein A binding^51^. Antibodies were produced in Chinese hamster ovary cells, and CD20xCD3 and Clec12axCD3 bsAbs were isolated by selective protein A affinity chromatography.

### Human T cell-tumor cell co-culture assays

Healthy donor peripheral blood mononuclear cells (PBMCs) were purchased from ReachBio Research Labs. T cells were isolated by negative selection using the Dynabeads Untouched Human T cells kit (Invitrogen, catalog number 11344D). Freshly-isolated T cells were co-cultured with human tumor cell lines at indicated effector:target (E:T) ratios and CD3 bsAb for 24–48 hours.

### CRISPR transcriptional activation screen in JeKo-1 tumor cells

JeKo-1 tumor cells were sequentially transduced with dCas9-VP64 and MS2-p65-HSF1 lentiviruses at a MOI of 0.3. JeKo-1/dCas9/MS2 cells were transduced with the human SAM library at 500x library representation. JeKo-1/SAM library cells were set up in 48-hour killing assays with freshly-isolated human T cells at 500x library representation. JeKo-1/SAM cells for NGS analysis were harvested on day 0 after antibiotic selection (reference control sample), after T cell killing and after 10 population doublings. Genomic DNA was extracted and PCR of the integrated sgRNAs was performed on genomic DNA at 400x library representation as previously described^14^.

## Supplementary information

Supplementary Info.

## Data Availability

All data generated during this study, including sgRNA representation in each arm of the screen, are included in the published article and its Supplementary Information.
