# Supplementary material for: Genome-scale CRISPR activation screen uncovers tumor-intrinsic modulators of CD3 bispecific antibody efficacy
Source: Sci Rep. 2019 Dec 27;9:20068. doi: 10.1038/s41598-019-56670-x (PMC6934601; doi:10.1038/s41598-019-56670-x)
Supplement: Supplementary file 1 — Supplementary Info. [file 41598_2019_56670_MOESM1_ESM.pdf]

Supplemental Materials

Title: Genome-scale CRISPR activation screen uncovers tumor-intrinsic modulators of CD3 bispecific antibody efficacy

Authors: Corinne E. Decker, Tara Young, Elizabeth Pasnikowski, Joyce Chiu, Hang Song, Yi Wei, Gavin Thurston and Christopher Daly*

-Supplemental Methods
-9 Supplemental Figures
-3 Supplemental Tables

Supplemental Methods

Quantification of CD20 surface levels. For each tumor cell line, 1 million cells were stained on ice with Quantibrite anti-CD20-PE (BD Biosciences, catalog number 347201) according to manufacturer's protocol and acquired on a BD LSR Fortessa. Antibodies bound per cell were calculated using a standard curve generated with the PE Fluorescence Quantitation Kit (BD Biosciences, catalog number 340495).

CellTrace labeling. Tumor cells were resuspended at 1 million cells/ml in PBS and loaded with 2.5 uM CellTrace Violet (Invitrogen Molecular Probes, catalog number C34557) or 4 uM CellTrace Yellow (Invitrogen Molecular Probes, catalog number C34567) for 15 minutes at 37°C, followed by quenching with complete media. For FACS conjugation assays, freshly-isolated human T cells were resuspended at 2 million cells/ml in PBS and loaded with 5 uM CellTrace CFSE (Invitrogen Molecular Probes, catalog number C34554) for 8 minutes at room temperature, followed by quenching with complete media.

T cell activation, tumor cell killing, and cell clustering assays. Freshly-isolated T cells were co-cultured with human tumor cell lines at indicated effector:target (E:T) ratios and CD3 bsAb for 24-48 hours. At each time point, cells were collected and stained with fixable viability dye eFluor 780 (Invitrogen eBioscience, catalog number 65-0865-14). In T cell activation experiments, cells were stained with anti-CD4-PE-Cy7 (clone OKT4, BioLegend, catalog number 317414), anti-CD8-BUV737 (clone SK1, BD Biosciences, catalog number 564629), anti-Ki-67-AlexaFluor 647 (clone Ki-67, BioLegend, catalog number 350510), anti-IFNg-Brilliant Violet 711 (clone 4S.B3, BioLegend, catalog number 502540), and anti-Granzyme B-FITC (clone GB11, BioLegend, catalog number 515403). Intracellular staining was performed using the eBioscience Foxp3 kit (Invitrogen, catalog number 00-5523-00) according to manufacturer's protocol. Samples were acquired on a BD LSR Fortessa, and the absolute number of live tumor cells in each sample was quantified using Countbright Absolute Counting Beads (Invitrogen Molecular Probes, catalog number C36950). For cell clustering assays, CellTrace-loaded T cells and tumor cells were mixed and treated with indicated concentrations of CD3 bsAb for 4-18 hours. At each time point, cells were transferred to FACS tubes containing 4% paraformaldehyde (Electron Microscopy Services, catalog number 15710) and immediately acquired on a BD LSR Fortessa.

CRISPR SAM library reagent preparation. The genome-scale human transcriptional activation SAM library was purchased from Genscript and amplified at Regeneron. Briefly, Lucigen E.coli 10G Elite electrocompetent cells (Lucigen, catalog number 80026-1) were transformed with the library by electroporation and plated on carbenicillin LB agar. Colonies were harvested, and plasmid DNA was extracted using the EndoFree Plasmid Maxi Prep (Qiagen, catalog number #12362). Sequencing of the original and amplified plasmids confirmed maintenance of library representation. Plasmids encoding the SAM components dCas9-VP64 and MS2-p65-HSF1 were purchased from Genscript. Each plasmid was transfected into HEK293T cells along with lentivirus packaging plasmids psPAX2 (Addgene, catalog number 12260) and pMD2.G (Addgene, catalog number 12259) using Lipofectamine (Thermo Fisher, catalog number 18324020), and virus-containing culture supernatant was concentrated by ultracentrifugation through a 25% sucrose cushion.

CRISPR transcriptional activation screen in JeKo-1 tumor cells. The JeKo-1/dCas9/MS2 cell line was established in blasticidin and hygromycin antibiotic selection. For expression of SAM library sgRNAs, JeKo-1/dCas9/MS2 cells were transduced at 500x library representation, followed by zeocin antibiotic selection for 7 days. JeKo-1/SAM library cells were set up in killing assays with freshly-isolated human T cells at 500x library representation. After 48 hours of treatment with 30 ng/ml CD20xCD3 bsAb, T cells were depleted using the EasySep Human CD3 Positive Selection Kit II (Stemcell, catalog number 17851), and surviving tumor cells were recovered in complete media. Surviving JeKo-1/SAM cells were expanded, and the killing assay was repeated as previously described to enrich for resistant tumor cells. In parallel with the T cell killing assays, JeKo-1/SAM library-expressing cells were cultured in complete media for 10 population doublings. JeKo-1/SAM cells for NGS analysis were harvested on day 0 after antibiotic selection (reference control sample), after T cell killing and after 10 population doublings. At each time point, cells were washed in PBS, pelleted, and preserved at -80°C.

To validate potential hits from the screen, individual sgRNAs were purchased from Genscript, packaged in lentivirus as described above, transduced into JeKo-1/dCas9/MS2 cells at a MOI < 0.3, and selected in zeocin for 7 days. Cell surface expression of targeted genes was assessed by FACS using the following antibodies: anti-SPN-FITC (clone MEM-50, BioLegend catalog number

315204), anti-SPN-Brilliant Violet 421 (clone L60, BD Biosciences, catalog number 744813), anti-CD52-FITC (clone 4C8, BD Pharmingen, catalog number 563609), anti-MUC1-APC (clone 16A, BioLegend, catalog number 355608).

Genomic DNA extraction, PCR amplification/barcoding, and PCR product purification. Genomic DNA was extracted from cell pellets using the QIAamp Blood and Cell Culture DNA Maxi Kit (Qiagen, catalog number #13362) according to manufacturer's protocol. 40 parallel reactions (5 ug of genomic DNA/reaction) were amplified using NEBnext High Fidelity 2x Master Mix (New England Biolabs, catalog number M0541) in a single-step reaction of 22 cycles. Sequences of forward primers and barcoded reverse primers are provided in Supplemental methods. PCR products from all reactions were pooled, purified using the Qiagen PCR purification kit (catalog number 28106), and gel purified using the Zymoclean Gel DNA Recovery Kit (Zymo, catalog number D4008). The purified PCR product was diluted to 10 ng/ul and deep-sequenced on an Illumina platform with a total coverage >45 million mapped reads for each sample.

Tumor cell growth assay. $2x10^4$ JeKo-1 cells/well were seeded in a 96-well plate in complete media on day 0. For MV-411 and THP-1 assays, $6x10^4$ cells were plated with 15 ng/ml Clec12a x CD3 in complete media. At each time point, CellTiter 96 Aqueous One Solution (Promega G3581) was added to each well and incubated for 1 hour at 37C. Absorbance at 490 nm was measured on a Spectramax M3 spectrophotometer (Molecular Devices).

24 hour killing assay with pre-activated T cells. Freshly-isolated T cells were co-cultured with parental JeKo-1 cells (3:1 E:T) and 30 ng/ml CD20xCD3 bsAb to stimulate T cell activation. After 24 hours, JeKo-1 cells were depleted from the culture using the EasySep Human CD19 Positive Selection Kit II (StemCell, catalog number 17854). Remaining T cells were re-plated with CellTrace-loaded tumor cells and indicated concentration of CD3 bsAb. After 24 hours, all cells were collected, stained with fixable viability dye, and acquired on a BD LSR Fortessa to quantify live tumor cells.

SPN overexpression in Raji cells. SPN cDNA was cloned into a pLX-IRES-Neo vector by Genscript. pLX/empty vector and pLX/SPN plasmids were transfected into HEK293T cells along with viral packaging plasmids psPAX2 and pMD2.G using Lipofectamine 2000 (Invitrogen, catalog number

11668019) according to the manufacturer's protocol. Virus-containing culture supernatant was concentrated using Lenti-X Concentrator (Takara, catalog number 63123) according to manufacturer's protocol. Lentiviruses were transduced into Raji cells and stably-expressing cell lines were established in G418 selection.

Neuraminidase and O-glycosidase treatment. Tumor cells were treated with 1 U/ml α(2→3,6,8,9) Neuraminidase from Arthrobacter ureafaciens (Sigma-Aldrich, catalog number N3786) and 0.2 U/ml O-Glycosidase from Streptococcus pneumoniae (Sigma-Aldrich, catalog number G1163). Following incubation for 3 hours at 37°C, cells were pelleted, lysed directly in SDS loading buffer and boiled. Cell lysates were separated by SDS-PAGE on a 7.5% polyacrylamide gel and subjected to western blotting for SPN (Abcam ab235453 rabbit monoclonal used at 1:1000, 4°C overnight)

Generation of SPN-KO cell lines. A gRNA targeting human SPN (GGATCCCACACCGTGACAGG) was cloned into the eSpCas9 pLentiCRISPR v2 lentiviral vector by Genscript. The Cas9 empty plasmid and Cas9/SPN gRNA plasmid were transfected into HEK293T cells along with viral packaging plasmids psPAX2 and pMD2.G using Lipofectamine 2000 (Invitrogen, catalog number 11668019) according to the manufacturer's protocol. Virus-containing culture supernatant was concentrated using Lenti-X Concentrator (Takara, catalog number 63123) according to manufacturer's protocol. Lentiviruses were transduced into tumor cells by spinfection at 1200g for 90 minutes on retronectin-coated plates (Takara, catalog number T100B). Stably-expressing cell lines were expanded in puromycin selection.

Forward and reverse primer sequences for SAM library PCR amplification

**Forward primers (each used in separate reactions to create sufficient library diversity)**	
F1	AAT GAT ACG GCG ACC ACC GAG ATC TAC ACT CTT TCC CTA CAC GAC GCT CTT CCG ATC TTA AGT AGA GGC TTT ATA TAT CTT GTG GAA AGG ACG AAA CAC C
F2	AAT GAT ACG GCG ACC ACC GAG ATC TAC ACT CTT TCC CTA CAC GAC GCT CTT CCG ATC TAT CAT GCT TAG CTT TAT ATA TCT TGT GGA AAG GAC GAA ACA CC
F3	AAT GAT ACG GCG ACC ACC GAG ATC TAC ACT CTT TCC CTA CAC GAC GCT CTT CCG ATC TGA TGC ACA TCT GCT TTA TAT ATC TTG TGG AAA GGA CGA AAC ACC
F4	AAT GAT ACG GCG ACC ACC GAG ATC TAC ACT CTT TCC CTA CAC GAC GCT CTT CCG ATC TCG ATT GCT CGA CGC TTT ATA TAT CTT GTG GAA AGG ACG AAA CAC C
F5	AAT GAT ACG GCG ACC ACC GAG ATC TAC ACT CTT TCC CTA CAC GAC GCT CTT CCG ATC TTC GAT AGC AAT TCG CTT TAT ATA TCT TGT GGA AAG GAC GAA ACA CC
F6	AAT GAT ACG GCG ACC ACC GAG ATC TAC ACT CTT TCC CTA CAC GAC GCT CTT CCG ATC TAT CGA TAG TTG CTT GCT TTA TAT ATC TTG TGG AAA GGA CGA AAC ACC
F7	AAT GAT ACG GCG ACC ACC GAG ATC TAC ACT CTT TCC CTA CAC GAC GCT CTT CCG ATC TGA TCG ATC CAG TTA GGC TTT ATA TAT CTT GTG GAA AGG ACG AAA CAC C
F8	AAT GAT ACG GCG ACC ACC GAG ATC TAC ACT CTT TCC CTA CAC GAC GCT CTT CCG ATC TCG ATC GAT TTG AGC CTG CTT TAT ATA TCT TGT GGA AAG GAC GAA ACA CC
F9	AAT GAT ACG GCG ACC ACC GAG ATC TAC ACT CTT TCC CTA CAC GAC GCT CTT CCG ATC TAC GAT CGA TAC ACG ATC GCT TTA TAT ATC TTG TGG AAA GGA CGA AAC ACC
F10	AAT GAT ACG GCG ACC ACC GAG ATC TAC ACT CTT TCC CTA CAC GAC GCT CTT CCG ATC TTA CGA TCG ATG GTC CAG AGC TTT ATA TAT CTT GTG GAA AGG ACG AAA CAC C
**Each reverse primer has a unique barcode (italicized)- use 1 reverse primer for each sample**	
R1	CAAGCAGAAGACGGCATACGAGAT*TCGCCTTG* GTGACTGGAGTTCAGACGTGTGCTCTTCCGATCTGCCAAGTTGATAACGGACTAGCCTT
R2	CAAGCAGAAGACGGCATACGAGAT*ATAGCGTC* GTGACTGGAGTTCAGACGTGTGCTCTTCCGATCTGCCAAGTTGATAACGGACTAGCCTT
R3	CAAGCAGAAGACGGCATACGAGAT*GAAGAAGT* GTGACTGGAGTTCAGACGTGTGCTCTTCCGATCTGCCAAGTTGATAACGGACTAGCCTT
R4	CAAGCAGAAGACGGCATACGAGAT*ATTCTAGG* GTGACTGGAGTTCAGACGTGTGCTCTTCCGATCTGCCAAGTTGATAACGGACTAGCCTT
R5	CAAGCAGAAGACGGCATACGAGAT*CGTTACCA* GTGACTGGAGTTCAGACGTGTGCTCTTCCGATCTGCCAAGTTGATAACGGACTAGCCTT
R6	CAAGCAGAAGACGGCATACGAGAT*GTCTGATG* GTGACTGGAGTTCAGACGTGTGCTCTTCCGATCTGCCAAGTTGATAACGGACTAGCCTT
R7	CAAGCAGAAGACGGCATACGAGAT*TTACGCAC* GTGACTGGAGTTCAGACGTGTGCTCTTCCGATCTGCCAAGTTGATAACGGACTAGCCTT
R8	CAAGCAGAAGACGGCATACGAGAT*TTGAATAG* GTGACTGGAGTTCAGACGTGTGCTCTTCCGATCTGCCAAGTTGATAACGGACTAGCCTT
R9	CAAGCAGAAGACGGCATACGAGAT*TCCTTGGT* GTGACTGGAGTTCAGACGTGTGCTCTTCCGATCTGCCAAGTTGATAACGGACTAGCCTT
R10	CAAGCAGAAGACGGCATACGAGAT*ACAGGTAT* GTGACTGGAGTTCAGACGTGTGCTCTTCCGATCTGCCAAGTTGATAACGGACTAGCCTT
R11	CAAGCAGAAGACGGCATACGAGAT*AGGTAAGG* GTGACTGGAGTTCAGACGTGTGCTCTTCCGATCTGCCAAGTTGATAACGGACTAGCCTT
R12	CAAGCAGAAGACGGCATACGAGAT*AACAATGG* GTGACTGGAGTTCAGACGTGTGCTCTTCCGATCTGCCAAGTTGATAACGGACTAGCCTT
R13	CAAGCAGAAGACGGCATACGAGAT*ACTGTATC* GTGACTGGAGTTCAGACGTGTGCTCTTCCGATCTGCCAAGTTGATAACGGACTAGCCTT

Supplemental Figure 1. CD4+ and CD8+ T cells are activated in a mixed killing assay of JeKo-1 and RL tumor cells

Healthy donor T cells were cultured with a 1:1 mix of JeKo-1 and RL tumor cells and treated with 30 ng/ml CD20xCD3 bsAb or CD3-binding isotype control antibody for 48 hours followed by staining for IFNγ and Granzyme B (GzmB)

Supplemental Figure 2. Surface expression of potential T cell ligands on Raji, RL, and JeKo-1 tumor cells

Surface expression of T cell ligands on tumor cells was assessed using the LEGENDscreen Human PE kit. Histograms show the expression of well-characterized co-stimulatory or co-inhibitory molecule expression on each cell line.

Supplemental Figure 3. Validation of CRISPR-mediated transcriptional activation in JeKo-1/dCas9/MS2 cells

- **(A)** JeKo-1/dCas9/MS2 cells were transduced with a non-targeting control sgRNA or sgRNA targeting SERPINB9. Following antibiotic selection, cells were lysed and subjected to Western blot analysis to detect SERPINB9 protein levels. Uncropped blots shown in **Figure S8E**.
- **(B)** JeKo-1/dCas9/MS2 cells were transduced with a non-targeting control sgRNA or sgRNAs targeting PD-L1. Following antibiotic selection, cells were lysed and subjected to Western blot analysis to detect PD-L1 protein levels. Uncropped blots shown in **Figure S8F**.
- **(C)** FACS detection of surface PD-L1 in JeKo-1/dCas9/MS2 cells transduced with non-targeting control sgRNA or sgRNAs targeting PD-L1.

Supplemental Figure 4. Validation of JeKo/SAM CRISPR screen hits

- **(A)** Comparison of normalized sgRNA counts in the tumor cell population collected after 10 population doublings in vitro compared to tumor cells on day 0. Normalized sgRNA counts were averaged across triplicate samples. R^2 value calculated by Pearson's correlation.

(B-D) Three sgRNAs each for SPN **(B)**, CD52 **(C)**, and MUC1 **(D)** were individually packaged in lentivirus and transduced into JeKo-1/dCas9/MS2 cells. Transduced tumor cells were stained with specific antibodies to measure cell surface levels of the targeted gene. For each gene, the 2 sgRNAs that were significantly enriched after CD20xCD3-mediated T cell killing in the screen are shown in boxes.

(C) Viable cell number of JeKo-1/NT, JeKo-1/SPN. , JeKo-1/CD52, and JeKo-1/MUC1 was measured on day 0 at the time of plating and after 24 hours in complete media. Increased expression of SPN, CD52 or MUC1 does not augment JeKo-1 cell growth in a 24-hour period

Supplemental Figure 5. MUC1 limits clustering of Jeko-1 cells with T cells and decreases killing

- **(A)** A competition killing assay was performed to evaluate the effect of MUC1 overexpression on JeKo-1 cell sensitivity to CD20xCD3-mediated T cell killing. Representative FACS plots show the proportions of live JeKo-1/NT and JeKo-1/MUC1

cells after 24 hours of co-culture with T cells and CD20xCD3 bsAb or CD3-binding isotype control antibody.

(B) The ratio of live JeKo-1/MUC1 cells to JeKo-1/NT cells was calculated after treatment with CD20xCD3 bsAb or CD3-binding isotype control antibody. JeKo-1/dCas9/MS2 cells expressing MUC1 sg1 (which effectively induces MUC1 expression and was enriched in the screen) were less sensitive to T cell killing, while the ineffective sg2 (fails to induce MUC1 expression) did not confer protection from killing. $**P < 0.01$, by two-tailed T-test.

(C) To detect T cell-tumor cell clusters, healthy donor T cells were labeled with CellTrace CFSE and tumor cells were labeled with CellTrace Violet (CTV) or CellTrace Yellow (CTY). Differentially labeled tumor cells were mixed at a 1:1 ratio and co-cultured with labeled T cells (3:1 E:T) plus 30 ng/ml CD20xCD3 bsAb or CD3-binding isotype control antibody. After 18 hours, cells were collected, fixed in 4% paraformaldehyde and immediately analyzed by FACS. Representative plots show the detection of T cell-tumor cell clusters. JeKo-1/NT cells in T cell conjugates are CTV+ FITC+, and JeKo-1/MUC1 cells in T cell conjugates are CTY+ FITC+. Note that MUC1 sg1 is highly effective at inducing MUC1 expression while sg2 is ineffective.

(D) The average percent of tumor cells in conjugates with T cells was quantified across triplicate samples. The extent of clustering was not affected by CellTrace dyes. $***P < 0.001$, by two-tailed T-test.

Supplemental Figure 6. CD20 surface expression is decreased in JeKo-1/CD52 cells

(A) CD20 surface expression on modified JeKo-1/dCas9/MS2 cells was quantified by staining with Quantibrite anti-CD20. CD20 antigen density is reduced on JeKo-1/CD52 cells.

(B) Total CD52 protein was assessed by Western blot analysis of JeKo-1/NT and JeKo-1/CD52 cells. Uncropped blots shown in **Figure S9G**.

Supplemental Figure 7. SPN knockout in RL cells

(A) Western blot detection of SPN protein in total cell lysates confirms SPN knockout in targeted RL cells. Uncropped blots shown in **Figure S9H**

(B) A competition killing assay was performed to evaluate the effect of SPN knockout on RL cell sensitivity to CD20xCD3-mediated T cell killing. RL cells transduced with the effective gRNA2 were mixed with RL cells expressing the ineffective gRNA3 (retain SPN expression). The extent of killing of each RL cell population was quantified after 48 hrs of co-culture with T cells and CD20xCD3.

(C) The ratio of live RL/SPN KO (g2) to RL (g3) cells was calculated after treatment with CD20xCD3 bsAb or CD3-binding isotype control antibody.* $P< 0.05$, ** $P < 0.01$, by two-tailed T-test

Supplemental Figure 8. SPN knockout in AML cell lines

(A) Western blot detection of SPN protein in total cell lysates confirms SPN knockout in targeted MV-4-11 and THP-1 cells. Uncropped blots shown in **Figure S9I**.

(B) Representative plots showing the detection of T cell-tumor cell clusters. SPN KO augments clustering of MV-4-11 cells

(C) Representative plots showing the detection of T cell-tumor cell clusters. SPN KO augments clustering of THP-1 cells

(D) MV-4-11/Cas9 and MV-4-11/SPN KO cells were cultured with 15 ng/ml Clec12a x CD3 bsAb for 24 hours and relative cell growth was determined.

(E) THP-1/Cas9 and THP-1/SPN KO cells were cultured with 15 ng/ml Clec12a x CD3 bsAb for 24 hours and relative cell growth was determined.

Supplemental Figure 9. Uncropped Western blots

(A) Uncropped blots corresponding to Figure 6A.
(B) Uncropped blots corresponding to Figure 6B.
(C) Uncropped blots corresponding to Figure 6C.
(D) Uncropped blots corresponding to Figure 6F.
(E) Uncropped blots corresponding to Figure S3A.
(F) Uncropped blots corresponding to Figure S3B.
(G) Uncropped blots corresponding to Figure S6B.
(H) Uncropped blots corresponding to Figure S7A.
(I) Uncropped blots corresponding to Figure S8A.

Supplemental Table 1. sgRNAs enriched ≧1.5-fold after T cell killing compared to Day 0 reference control, $p < 0.05$

Supplemental Table 2. Genes with at least 2 sgRNAs enriched ≧1.5-fold after T cell killing compared to Day 0 reference control, $p < 0.05$

Supplemental Table 3. sgRNAs enriched ≧ 1.5-fold after 10 population doublings compared to Day 0 reference control, $p < 0.05$

Supplemental Figure 1 CD4+ and CD8+ T cells are activated in a mixed killing assay of JeKo-1 and RL tumor cells

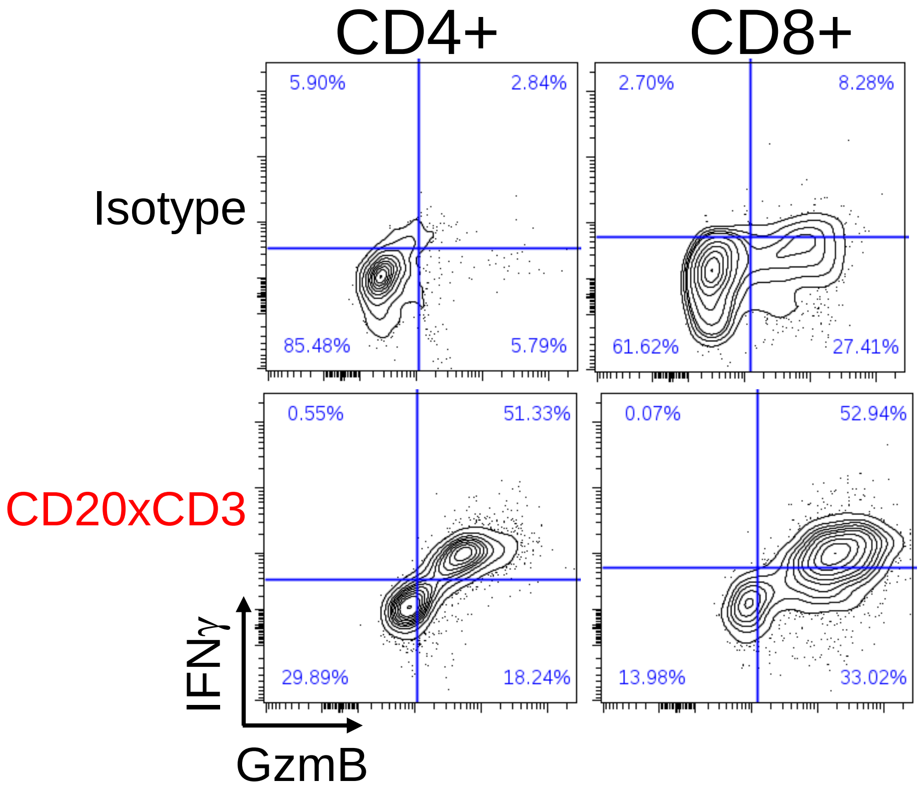

Supplemental Figure 2. LEGENDscreen: surface expression of potential T cell ligands on Raji, RL, and JeKo-1 tumor cells

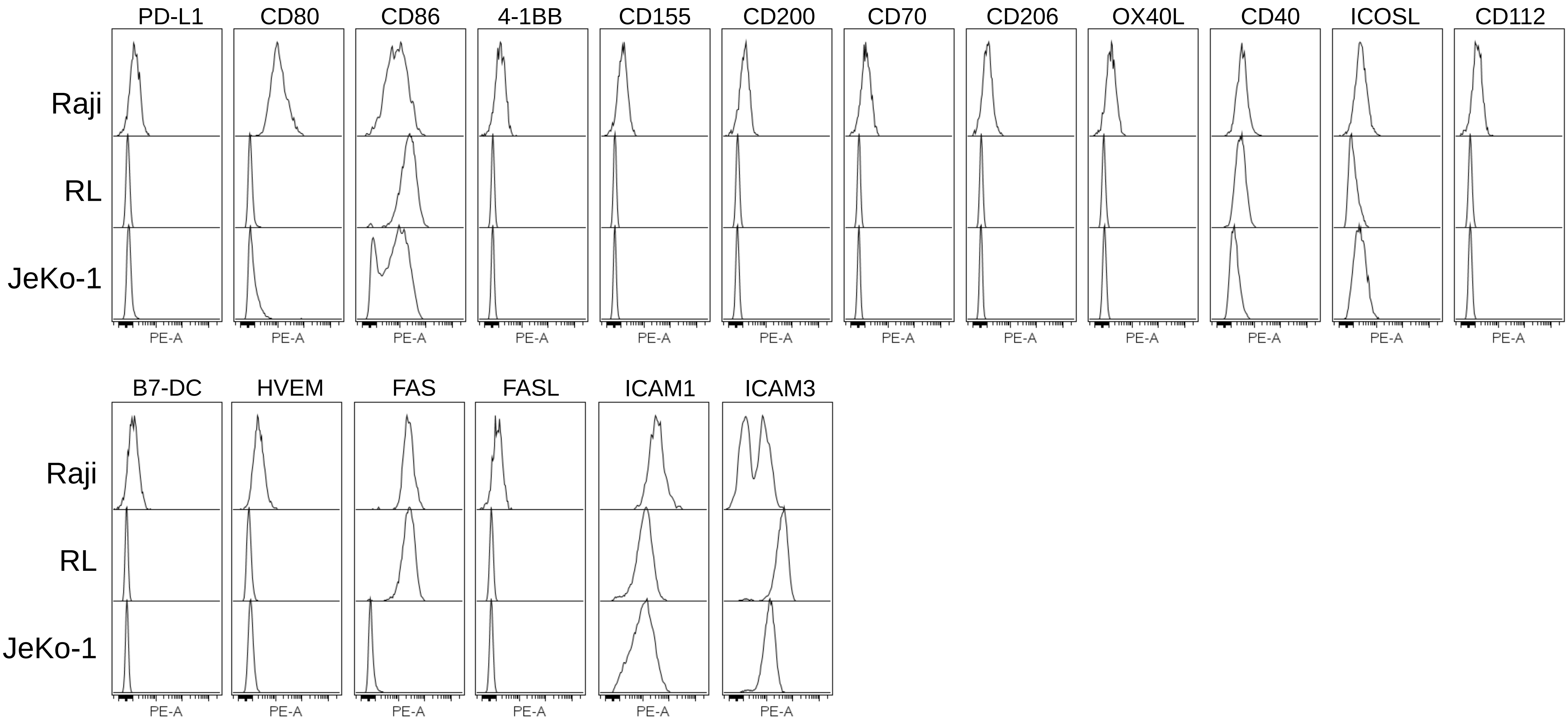

Supplemental Figure 3. Validation of JeKo/dCas9/MS2 cells

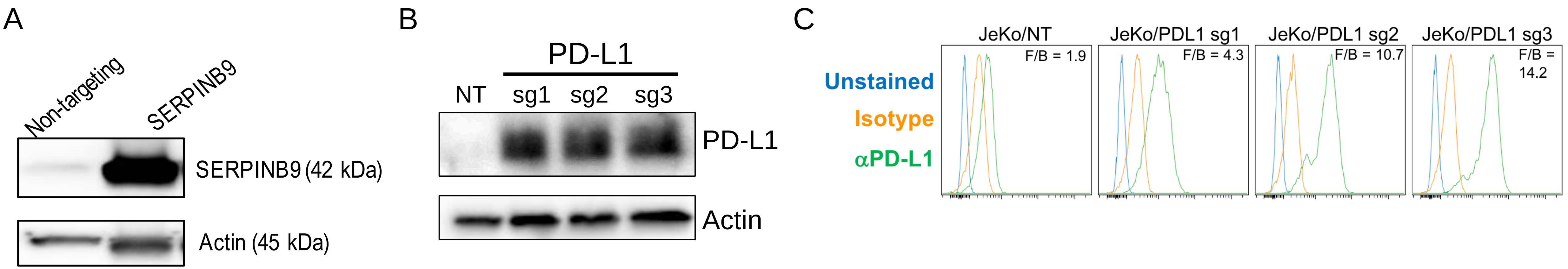

Supplemental Figure 4. Validation of JeKo/SAM CRISPR screen hits

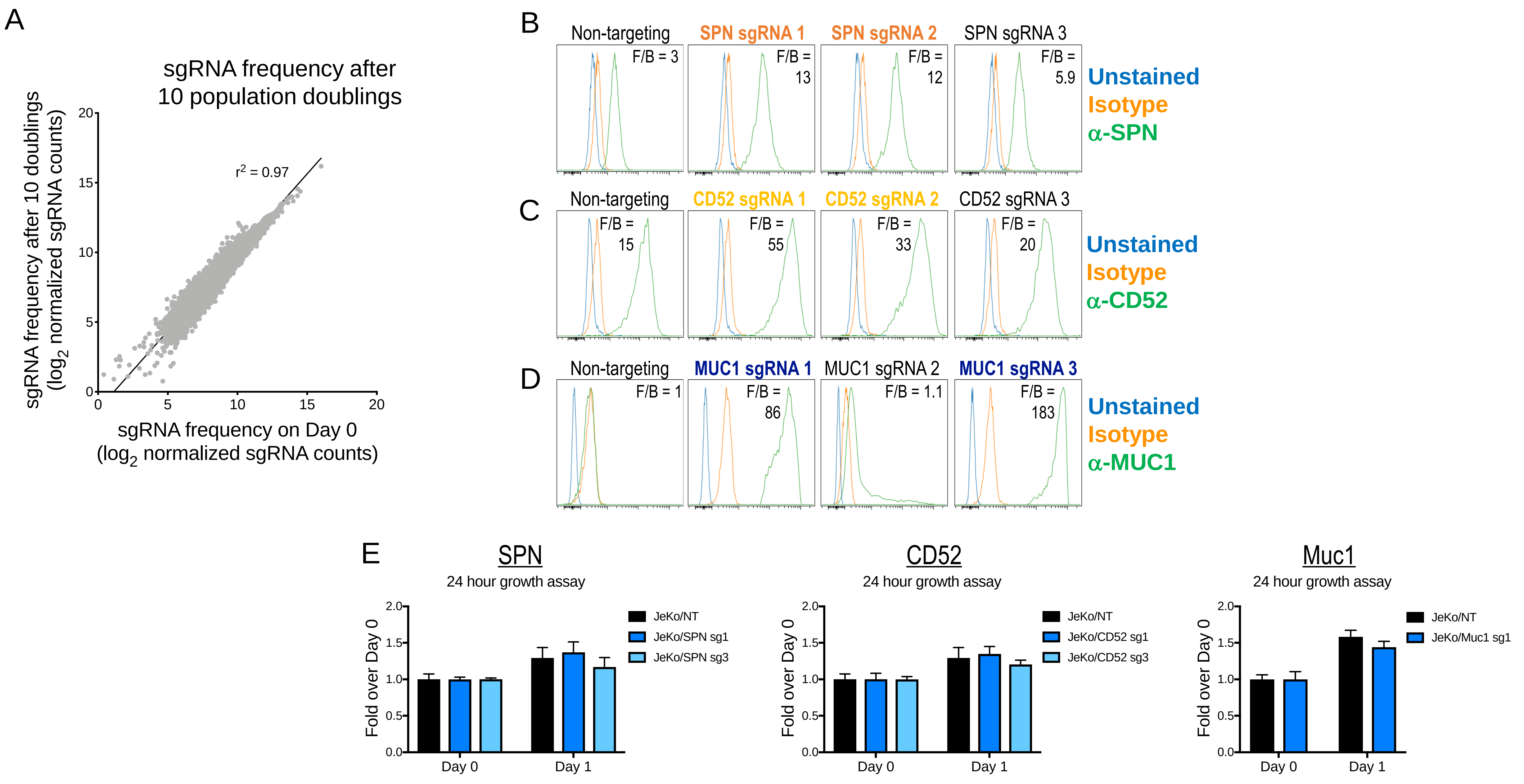

Supplemental Figure 5. Muc1 limits CD20xCD3-mediated JeKo-1 cell killing and clustering with T cells

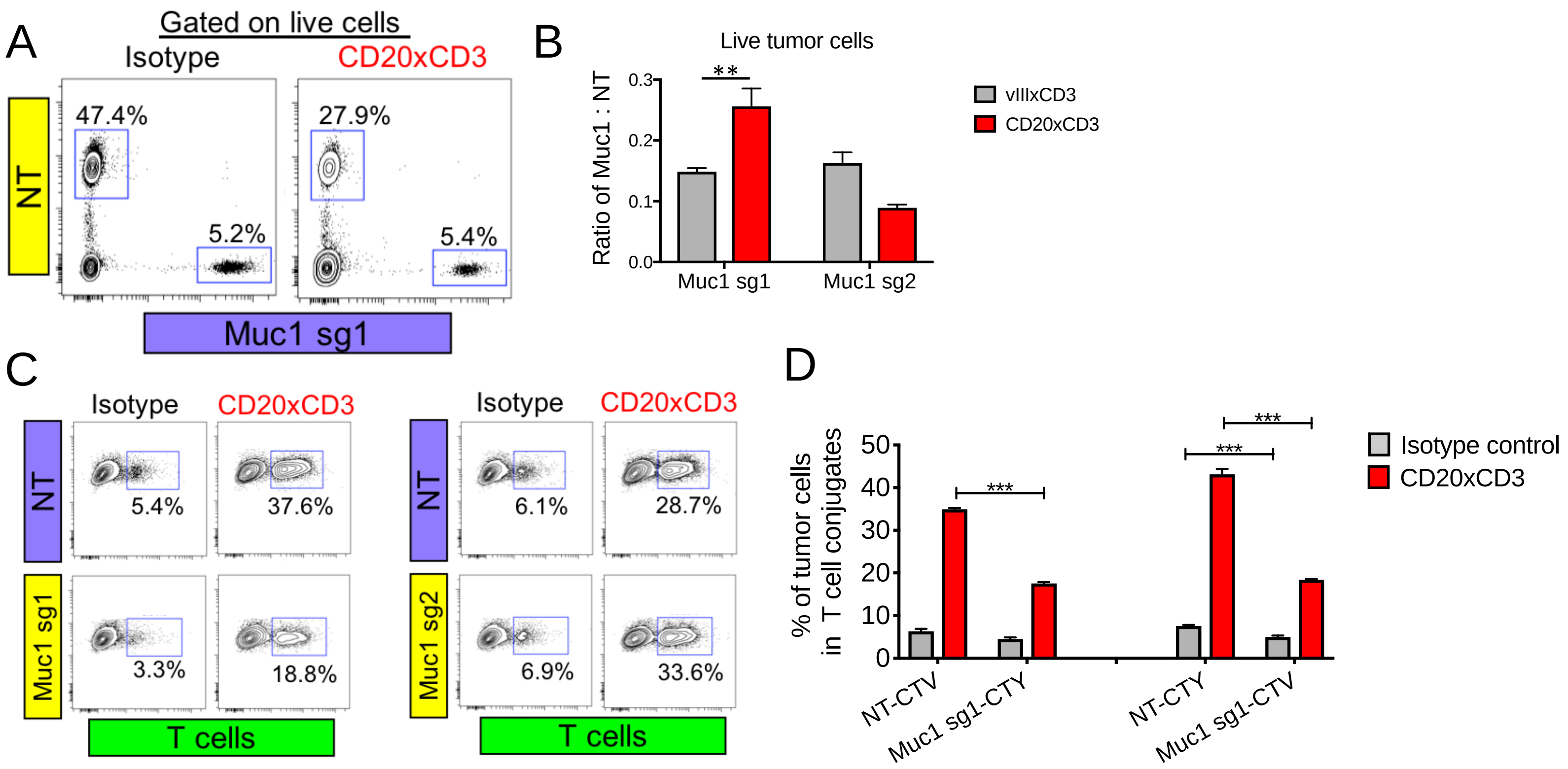

Supplemental Figure 6 CD20 surface expression is decreased in JeKo/CD52 cells

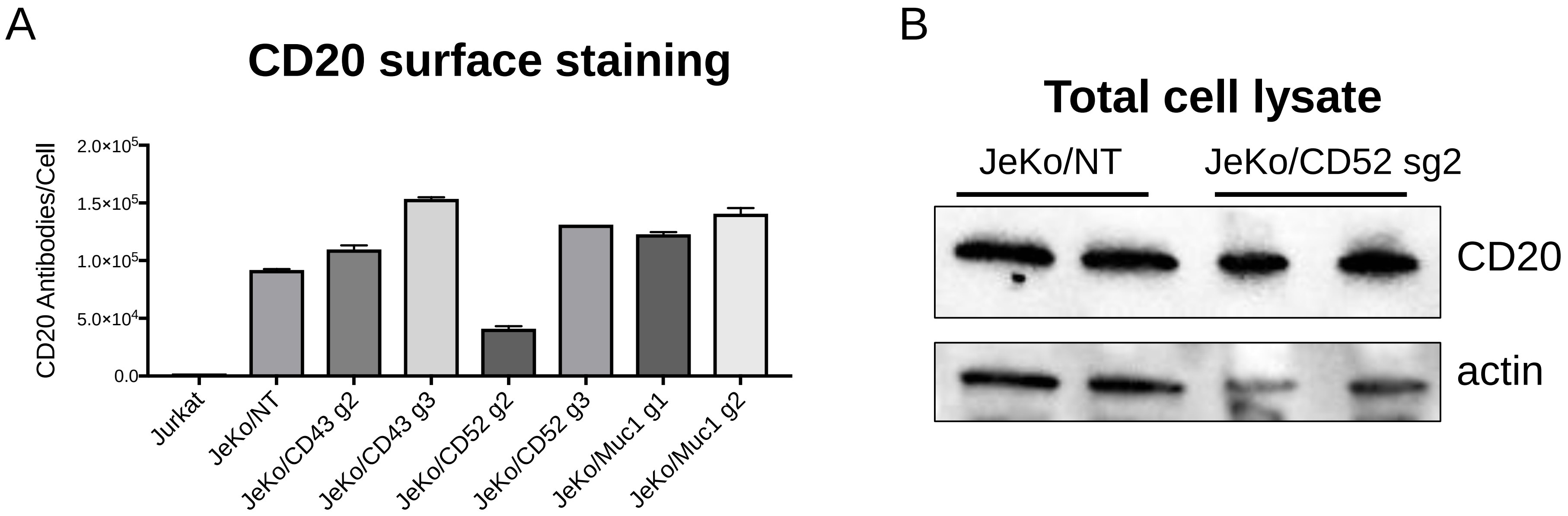

Supplemental Figure 7 SPN knockout in RL cells

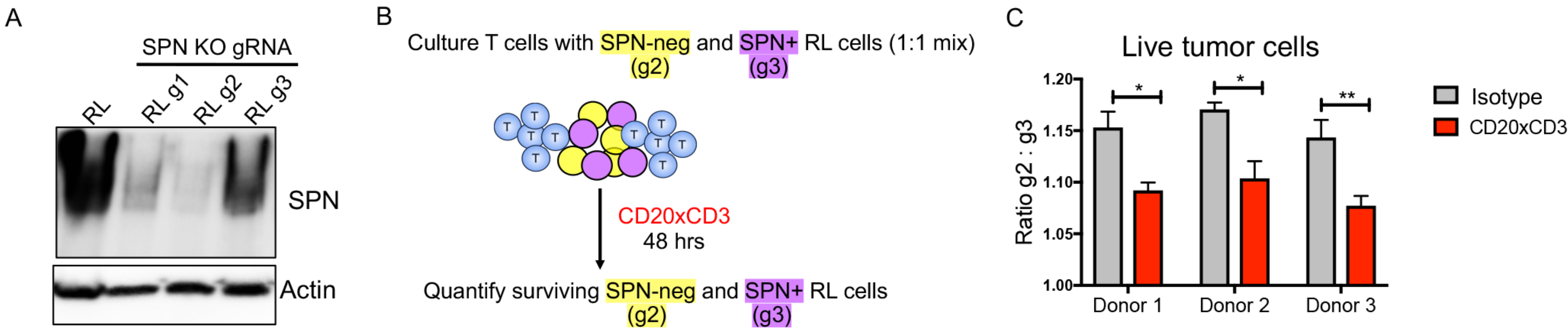

Supplemental Figure 8 SPN knockout in AML cell lines

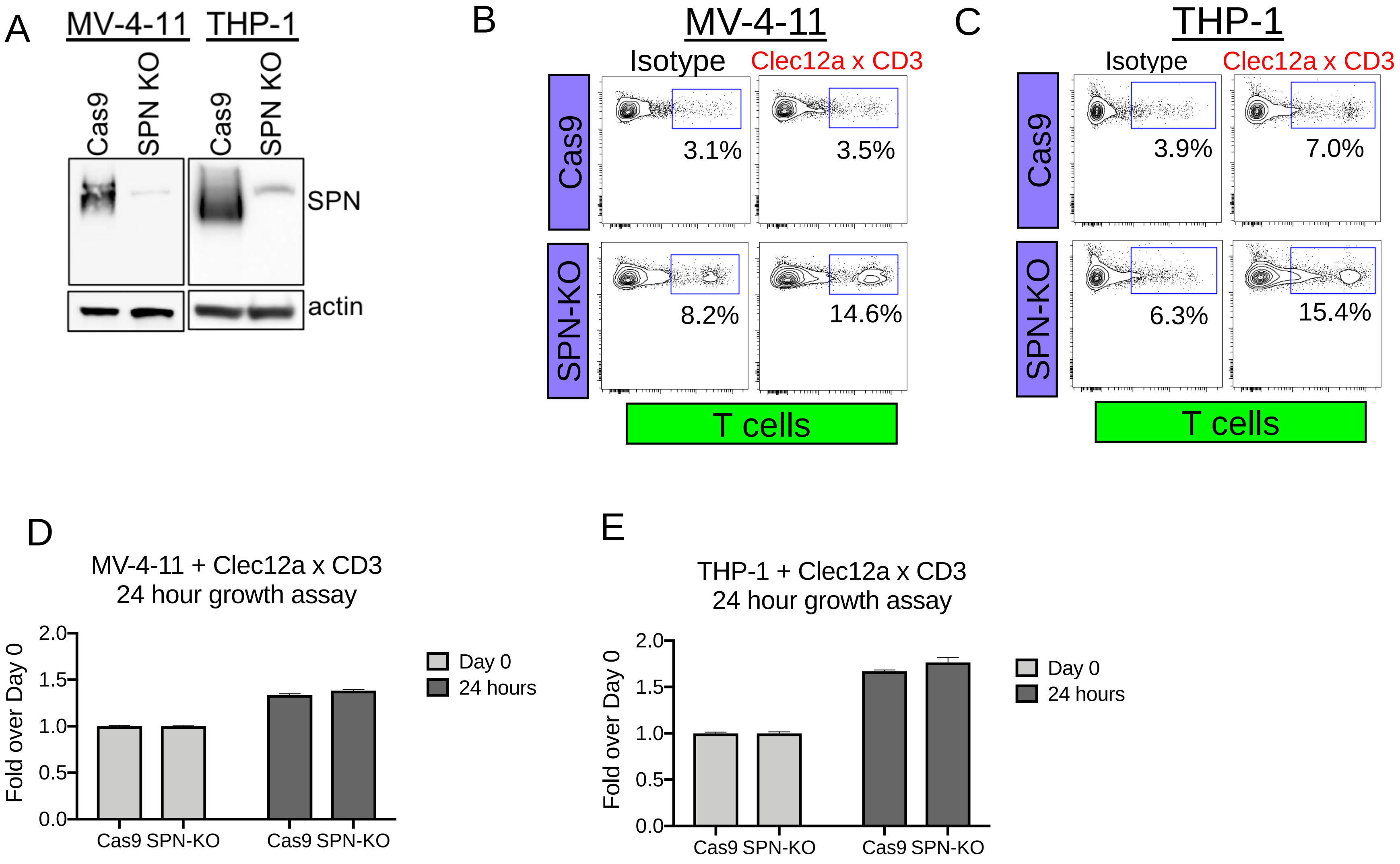

Supplemental Figure 9. Uncropped Western blots

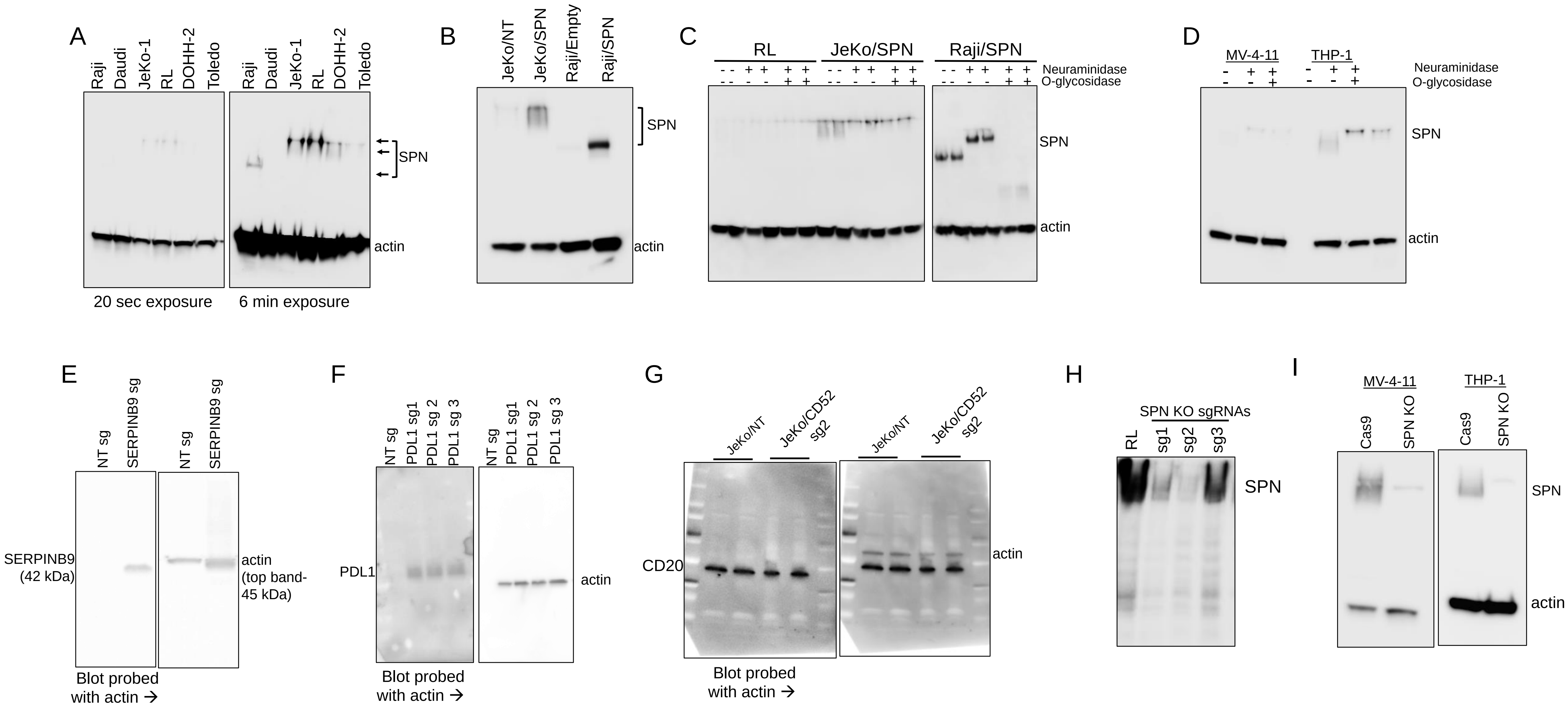

Supplemental Table 1. sgRNAs enriched ≧1.5-fold after T cell killing compared to Day 0 reference control, $p < 0.05$

Enrichment over Day 0	T-Test	Access. Number	Gene Symbol
27.63	0.0221	NM_014619	*GRIK4*
12.20	0.0056	NM_001204294	*MUC1*
10.15	0.0003	NM_001136205	*KCTD1*
9.09	0.0255	NM_001037171	*ACOT9*
8.88	0.0282	NM_001172697	*TSFM*
8.13	0.0123	NM_152629	*GLIS3*
7.66	0.0299	NM_002865	*RAB2A*
7.63	0.0230	NM_001525	*HCRTR1*
7.22	0.0391	NM_177454	*FAM171B*
6.93	0.0331	NM_020851	*ISLR2*
6.31	0.0159	NM_001143680	*GSTK1*
6.24	0.0499	NM_176815	*DHFR2*
5.76	0.0032	NM_001803	*CD52*
5.60	0.0097	NM_080552	*SLC32A1*
5.57	0.0272	NM_139283	*PPTC7*
5.47	0.0308	NM_001010909	*MUC21*
5.11	0.0413	NM_001030288	*SPN*
5.10	0.0496	NM_001030288	*SPN*
4.96	0.0002	NM_173598	*KSR2*
4.94	0.0083	NM_023009	*MARCKSL1*
4.85	0.0004	NM_002699	*POU3F1*
4.68	0.0157	NM_022550	*XRCC4*
4.58	0.0085	NM_016240	*SCARA3*
4.57	0.0004	NM_014079	*KLF15*
4.53	0.0236	NM_001079881	*PRKD2*
4.44	0.0063	NM_001803	*CD52*
4.28	0.0344	NM_001278544	*C8B*
4.27	0.0266	NM_001425	*EMP3*
4.23	0.0145	NM_001098205	*HNRNPF*
4.12	0.0130	NM_001172664	*RAB40C*
4.10	0.0313	NM_003123	*SPN*
4.07	0.0035	NM_000744	*CHRNA4*
4.04	0.0184	NM_032511	*FAXC*
3.95	0.0467	NM_174892	*CD300LB*

Enrichment over Day 0	T-Test	Access. Number	Gene Symbol
3.88	0.0068	NM_207391	*RGS9BP*
3.85	0.0131	NM_001289823	*FURIN*
3.82	0.0046	NM_017613	*DONSON*
3.78	0.0292	NM_003259	*ICAM5*
3.77	0.0080	NM_006412	*AGPAT2*
3.64	0.0027	NM_005304	*FFAR3*
3.56	0.0000	NM_153046	*TDRD9*
3.54	0.0164	NM_001079530	*CFC1B*
3.53	0.0047	NM_020979	*SH2B2*
3.44	0.0471	NM_003290	*TPM4*
3.41	0.0005	NM_005603	*ATP8B1*
3.40	0.0022	NM_001278116	*L1CAM*
3.39	0.0274	NM_001256302	*ETF1*
3.35	0.0253	NM_006010	*MANF*
3.34	0.0233	NM_145109	*MAP2K3*
3.34	0.0333	NM_080622	*ABHD16B*
3.33	0.0126	NM_002698	*POU2F2*
3.33	0.0460	NM_001513	*GSTZ1*
3.26	0.0489	NM_201550	*LRRC10*
3.25	0.0091	NM_001280	*CIRBP*
3.24	0.0374	NM_001253901	*MEST*
3.21	0.0108	NM_001110354	*ZP3*
3.18	0.0186	NM_138433	*KLHDC7B*
3.18	0.0003	NM_001004470	*ST8SIA6*
3.14	0.0248	NM_032152	*PRAM1*
3.10	0.0457	NM_001134775	*KLC2*
3.08	0.0475	NM_006278	*ST3GAL4*
3.03	0.0482	NM_001098204	*HNRNPF*
3.02	0.0003	NM_001164688	*RD3*
3.02	0.0290	NM_001290061	*SEMA3B*
3.02	0.0174	NM_001198656	*AKAP2*
3.01	0.0140	NM_002135	*NR4A1*
3.01	0.0013	NM_006213	*PHKG1*
3.00	0.0249	NM_001145033	*C11orf96*
3.00	0.0005	NM_020992	*PDLIM1*
2.99	0.0150	NM_000232	*SGCB*
2.97	0.0039	NM_001270616	*PROX1*

Enrichment over Day 0	T-Test	Access. Number	Gene Symbol
2.95	0.0423	NM_001257378	*SEC14L3*
2.95	0.0066	NM_020871	*LRCH2*
2.95	0.0134	NM_001012728	*DPRX*
2.95	0.0226	NM_005033	*EXOSC9*
2.94	0.0009	NM_002630	*PGC*
2.93	0.0456	NM_005113	*GOLGA5*
2.92	0.0324	NM_182488	*USP12*
2.92	0.0366	NM_019595	*ITSN2*
2.91	0.0431	NM_018842	*BAIAP2L1*
2.91	0.0259	NM_153240	*NPHP3*
2.89	0.0479	NM_018017	*CCDC186*
2.85	0.0114	NM_001012279	*SOGA3*
2.82	0.0165	NM_001270991	*EPGN*
2.81	0.0090	NM_000391	*TPP1*
2.81	0.0039	NM_024761	*MOB3B*
2.80	0.0340	NM_001135776	*ZBTB43*
2.80	0.0285	NM_001048195	*RCC1*
2.76	0.0083	NM_001286693	*RGS22*
2.76	0.0230	NM_024808	*BORA*
2.76	0.0005	NM_000524	*HTR1A*
2.76	0.0001	NM_197962	*GLRX2*
2.75	0.0397	NM_024531	*SLC52A2*
2.74	0.0001	NM_001009568	*SMPDL3B*
2.74	0.0171	NM_001257392	*CD63*
2.72	0.0416	NM_003044	*SLC6A12*
2.71	0.0307	NM_001113203	*NACA*
2.70	0.0269	NM_001282765	*CDV3*
2.70	0.0424	NM_001014380	*KATNAL1*
2.69	0.0335	NM_001277163	*CEACAM3*
2.69	0.0007	NM_138350	*THAP3*
2.69	0.0010	NM_022659	*EBF2*
2.67	0.0503	NM_004429	*EFNB1*
2.66	0.0117	NM_001160226	*CNR1*
2.66	0.0109	NM_207116	*RNF216*
2.66	0.0079	NM_207316	*TMEM207*
2.65	0.0052	NM_145044	*ZNF501*
2.65	0.0314	NM_001005855	*ATP8B2*

Enrichment over Day 0	T-Test	Access. Number	Gene Symbol
2.64	0.0085	NM_001080442	*SLC38A8*
2.63	0.0267	NM_006662	*SRCAP*
2.63	0.0000	NM_001168393	*CREBRF*
2.63	0.0238	NM_015140	*TTLL12*
2.62	0.0150	NM_145912	*NFAM1*
2.62	0.0200	NM_001085481	*MAP1LC3B2*
2.61	0.0013	NM_001278613	*PCDH1*
2.61	0.0057	NM_020873	*LRRN1*
2.60	0.0033	NM_006290	*TNFAIP3*
2.58	0.0041	NM_002509	*NKX2-2*
2.58	0.0004	NM_014434	*NDOR1*
2.55	0.0454	NM_003503	*CDC7*
2.54	0.0336	NM_015325	*ICE1*
2.54	0.0503	NM_006829	*ADIRF*
2.54	0.0020	NM_021960	*MCL1*
2.52	0.0131	NM_033554	*HLA-DPA1*
2.52	0.0487	NM_144992	*VWA3B*
2.50	0.0006	NM_001258346	*PACRGL*
2.50	0.0158	NM_205849	*FAM9B*
2.49	0.0414	NM_001160111	*NOS3*
2.49	0.0320	NM_032687	*CYHR1*
2.49	0.0007	NM_177543	*PLPP2*
2.49	0.0001	NM_001256510	*SSBP1*
2.48	0.0312	NM_001145276	*ZFY*
2.48	0.0038	NM_001127215	*GFI1*
2.47	0.0032	NM_001040100	*SPTSSB*
2.47	0.0182	NM_001252406	*ZBTB7B*
2.47	0.0159	NM_001267698	*CD63*
2.47	0.0217	NM_024959	*SLC8B1*
2.46	0.0479	NM_207352	*CYP4V2*
2.46	0.0220	NM_016175	*C5orf45*
2.46	0.0254	NM_001290104	*ZP2*
2.46	0.0136	NM_006984	*CLDN10*
2.45	0.0250	NM_001256653	*ZNF43*
2.45	0.0462	NM_001005241	*OR4N4*
2.44	0.0108	NM_199047	*TBPL2*
2.44	0.0001	NM_001282116	*RFX3*

Enrichment over Day 0	T-Test	Access. Number	Gene Symbol
2.44	0.0003	NM_024122	*APOO*
2.44	0.0376	NM_001127196	*CNBP*
2.43	0.0009	NM_001282741	*OSBP2*
2.43	0.0196	NM_175883	*OR7D2*
2.42	0.0061	NM_006577	*B3GNT2*
2.42	0.0188	NM_001162894	*KIAA0040*
2.42	0.0145	NM_152755	*CNPY4*
2.41	0.0258	NM_001012337	*ROPN1B*
2.41	0.0317	NM_002227	*JAK1*
2.40	0.0185	NM_080836	*STK35*
2.40	0.0357	NM_001110354	*ZP3*
2.40	0.0085	NM_024980	*GPR157*
2.40	0.0213	NM_001159587	*CD109*
2.40	0.0073	NM_032107	*L3MBTL1*
2.38	0.0342	NM_022479	*WBSCR17*
2.38	0.0143	NM_138723	*BCL2L14*
2.37	0.0023	NM_012316	*KPNA6*
2.37	0.0012	NM_002235	*KCNA6*
2.37	0.0046	NM_001282687	*SH3YL1*
2.36	0.0302	NM_001178045	*SLC44A4*
2.36	0.0093	NM_014357	*LCE2B*
2.36	0.0323	NM_016548	*GOLM1*
2.36	0.0271	NM_013305	*ST8SIA5*
2.35	0.0260	NM_001276469	*B4GALNT1*
2.34	0.0085	NM_015387	*MOB4*
2.34	0.0231	NM_001286754	*SYNPO2*
2.34	0.0402	NM_001244871	*DAB2*
2.32	0.0447	NM_145214	*TRIM11*
2.32	0.0167	NM_001130487	*NDRG4*
2.32	0.0435	NM_000537	*REN*
2.32	0.0029	NM_020744	*MTA3*
2.32	0.0249	NM_001029863	*C6orf120*
2.32	0.0087	NM_181361	*KCNMB2*
2.31	0.0152	NM_001082968	*TOM1L2*
2.30	0.0457	NM_178504	*DNAH12*
2.29	0.0339	NM_017676	*GIN1*
2.28	0.0228	NM_001076785	*SLC7A6*

Enrichment over Day 0	T-Test	Access. Number	Gene Symbol
2.28	0.0018	NM_002969	*MAPK12*
2.28	0.0121	NM_013453	*SPANXA1*
2.28	0.0093	NM_001191058	*PDE1C*
2.28	0.0008	NM_001282864	*SLC2A11*
2.28	0.0243	NM_001795	*CDH5*
2.28	0.0072	NM_006824	*EBNA1BP2*
2.27	0.0356	NM_001085420	*PLSCR5*
2.27	0.0087	NM_032693	*NAA11*
2.27	0.0468	NM_018416	*FOXJ2*
2.26	0.0003	NM_001256163	*BIRC2*
2.26	0.0399	NM_004760	*STK17A*
2.26	0.0275	NM_001193301	*SEMA4A*
2.26	0.0356	NM_183419	*RNF19A*
2.26	0.0424	NM_022463	*NXN*
2.25	0.0037	NM_206860	*TACC2*
2.24	0.0179	NM_022165	*LIN7B*
2.24	0.0190	NM_207401	*C1orf229*
2.24	0.0198	NM_033518	*SLC38A5*
2.24	0.0022	NM_005828	*DCAF7*
2.23	0.0144	NM_022153	*C10orf54*
2.23	0.0428	NM_001161357	*FCHO1*
2.22	0.0044	NM_001282965	*SLA*
2.22	0.0035	NM_001143827	*MAPRE2*
2.22	0.0085	NM_021962	*ABR*
2.21	0.0493	NM_005723	*TSPAN5*
2.21	0.0002	NM_001242860	*PRKACB*
2.21	0.0111	NM_018026	*PACS1*
2.21	0.0016	NM_001031700	*FAM198B*
2.21	0.0152	NM_000685	*AGTR1*
2.20	0.0003	NM_000056	*BCKDHB*
2.20	0.0298	NM_002940	*ABCE1*
2.19	0.0018	NM_012333	*MYCBP*
2.19	0.0216	NM_152470	*RNF165*
2.19	0.0022	NM_001127896	*CHST8*
2.18	0.0396	NM_001286771	*ANKRD17*
2.18	0.0038	NM_001267560	*TJP3*
2.18	0.0267	NM_001164405	*BHLHA9*

Enrichment over Day 0	T-Test	Access. Number	Gene Symbol
2.18	0.0283	NM_198320	*CPM*
2.17	0.0152	NM_001291410	*LOC653602*
2.17	0.0417	NM_001289401	*ZNF135*
2.17	0.0254	NM_001145648	*RASGRF1*
2.17	0.0346	NM_152263	*TPM3*
2.16	0.0066	NM_004599	*SREBF2*
2.16	0.0348	NM_001170331	*LANCL3*
2.16	0.0263	NM_005398	*PPP1R3C*
2.16	0.0088	NM_173824	*C3orf38*
2.16	0.0012	NM_024092	*TMEM109*
2.16	0.0220	NM_001013706	*PLIN5*
2.16	0.0414	NM_001039877	*STRN4*
2.16	0.0434	NM_001097622	*OCM*
2.16	0.0264	NM_001282290	*ARHGAP27*
2.15	0.0054	NM_022725	*FANCF*
2.15	0.0117	NM_001100592	*ATP6V0E2*
2.15	0.0245	NM_032451	*SPIRE2*
2.15	0.0345	NM_001005492	*OR5J2*
2.14	0.0365	NM_001282146	*NLGN4X*
2.14	0.0037	NM_000417	*IL2RA*
2.14	0.0325	NM_206827	*RASL11A*
2.14	0.0252	NM_012401	*PLXNB2*
2.14	0.0029	NM_023035	*CACNA1A*
2.14	0.0007	NM_003000	*SDHB*
2.13	0.0026	NM_000403	*GALE*
2.13	0.0399	NM_002863	*PYGL*
2.13	0.0046	NM_018890	*RAC1*
2.13	0.0105	NM_001145643	*PHGR1*
2.13	0.0134	NM_001002258	*ATP5G3*
2.13	0.0461	NM_002317	*LOX*
2.13	0.0339	NM_001142462	*OSR2*
2.12	0.0165	NM_178454	*DRAM2*
2.12	0.0090	NM_003893	*LDB1*
2.12	0.0010	NM_194454	*KRIT1*
2.12	0.0161	NM_001113491	*SEPT9*
2.11	0.0002	NM_001143764	*SYCE1*
2.11	0.0136	NM_000161	*GCH1*

Enrichment over Day 0	T-Test	Access. Number	Gene Symbol
2.11	0.0005	NM_001166434	*ITIH1*
2.11	0.0011	NM_004450	*ERH*
2.11	0.0055	NM_021960	*MCL1*
2.10	0.0174	NM_017837	*PIGV*
2.10	0.0377	NM_007373	*SHOC2*
2.10	0.0082	NM_001198695	*MFAP4*
2.09	0.0476	NM_001020819	*MYADM*
2.09	0.0441	NM_021965	*PGM5*
2.09	0.0094	NM_001159531	*BEGAIN*
2.09	0.0419	NM_001159576	*SCNN1A*
2.09	0.0157	NM_005263	*GFI1*
2.09	0.0063	NM_058244	*WNT8A*
2.08	0.0260	NM_002099	*GYPA*
2.08	0.0003	NM_138334	*JOSD2*
2.08	0.0016	NM_001146344	*PRAMEF11*
2.07	0.0052	NM_005534	*IFNGR2*
2.07	0.0020	NM_000363	*TNNI3*
2.06	0.0274	NM_024831	*TGS1*
2.06	0.0149	NM_138801	*GALM*
2.06	0.0442	NM_001206672	*RIC3*
2.06	0.0262	NM_001080556	*CFAP52*
2.06	0.0456	NM_001145077	*LRRC10B*
2.06	0.0304	NM_016026	*RDH11*
2.06	0.0156	NM_133640	*MED22*
2.05	0.0002	NM_001160302	*SYNJ1*
2.05	0.0118	NM_018694	*ARL6IP4*
2.05	0.0023	NM_014800	*ELMO1*
2.05	0.0344	NM_173598	*KSR2*
2.05	0.0361	NM_001105203	*RUSC1*
2.05	0.0212	NM_001207005	*ZNF233*
2.05	0.0034	NM_133433	*NIPBL*
2.04	0.0489	NM_001286680	*NPM2*
2.04	0.0029	NM_030807	*SLC2A11*
2.04	0.0386	NM_001145784	*BORCS8*
2.04	0.0011	NM_014725	*STARD8*
2.04	0.0017	NM_175850	*DNMT3B*
2.03	0.0427	NM_001168407	*RIMS1*

Enrichment over Day 0	T-Test	Access. Number	Gene Symbol
2.03	0.0491	NM_020753	*CASKIN2*
2.03	0.0306	NM_032039	*FAM234A*
2.03	0.0030	NM_003709	*KLF7*
2.03	0.0471	NM_017635	*KMT5B*
2.03	0.0476	NM_001407	*CELSR3*
2.03	0.0119	NM_004383	*CSK*
2.03	0.0293	NM_016216	*DBR1*
2.02	0.0283	NM_024552	*CERS4*
2.02	0.0447	NM_000521	*HEXB*
2.02	0.0242	NM_030622	*CYP2S1*
2.02	0.0077	NM_001271854	*GPR176*
2.01	0.0193	NM_148896	*NPB*
2.01	0.0339	NM_004657	*SDPR*
2.01	0.0433	NM_001083885	*WHRN*
2.01	0.0129	NM_001271823	*SERPINB6*
2.01	0.0005	NM_003468	*FZD5*
2.01	0.0285	NM_030765	*B3GNT4*
2.00	0.0178	NM_004406	*DMBT1*
2.00	0.0234	NM_001170779	*FAM122C*
2.00	0.0479	NM_032410	*HOOK3*
2.00	0.0114	NM_205833	*IGSF1*
2.00	0.0232	NM_013278	*IL17C*
2.00	0.0466	NM_198494	*ZFP69*
1.99	0.0226	NM_001085399	*RELL1*
1.99	0.0215	NM_020404	*CD248*
1.99	0.0422	NM_178836	*PLD6*
1.99	0.0323	NM_001258024	*SKOR1*
1.99	0.0188	NM_014938	*MLXIP*
1.99	0.0100	NM_001126049	*KLLN*
1.99	0.0001	NM_024038	*C19orf43*
1.99	0.0179	NM_001965	*EGR4*
1.99	0.0389	NM_017675	*CDHR2*
1.99	0.0483	NM_032596	*C9orf24*
1.99	0.0289	NM_000741	*CHRM4*
1.98	0.0039	NM_001184720	*GYG1*
1.98	0.0422	NM_020211	*RGMA*
1.98	0.0299	NM_053049	*UCN3*

Enrichment over Day 0	T-Test	Access. Number	Gene Symbol
1.98	0.0037	NM_001010927	*TIAM2*
1.98	0.0005	NM_001286649	*STK24*
1.98	0.0145	NM_001242474	*ZNF345*
1.98	0.0399	NM_033280	*SEC11C*
1.98	0.0037	NM_001243646	*CD2BP2*
1.98	0.0070	NM_001243135	*PELI3*
1.98	0.0095	NM_012342	*BAMBI*
1.98	0.0080	NM_000913	*OPRL1*
1.98	0.0004	NM_002507	*NGFR*
1.97	0.0090	NM_014332	*SMPX*
1.97	0.0152	NM_001143888	*BSDC1*
1.97	0.0180	NM_020870	*SH3RF1*
1.97	0.0005	NM_030765	*B3GNT4*
1.97	0.0004	NM_001286086	*C11orf98*
1.97	0.0106	NM_033255	*EPSTI1*
1.97	0.0169	NM_000081	*LYST*
1.97	0.0020	NM_014648	*DZIP3*
1.97	0.0230	NM_024337	*IRX1*
1.97	0.0387	NM_003712	*PLPP2*
1.97	0.0023	NM_001101362	*KBTBD13*
1.96	0.0044	NM_001005855	*ATP8B2*
1.96	0.0419	NM_018337	*ZNF444*
1.96	0.0005	NM_145644	*MRPL35*
1.96	0.0243	NM_006244	*PPP2R5B*
1.96	0.0100	NM_015508	*TIPARP*
1.96	0.0063	NM_001197222	*PDE4D*
1.96	0.0293	NM_147184	*TP53I3*
1.96	0.0010	NM_022818	*MAP1LC3B*
1.96	0.0025	NM_001284269	*EFCAB11*
1.96	0.0295	NM_130466	*UBE3B*
1.96	0.0150	NM_006681	*NMU*
1.96	0.0012	NM_031476	*CRISPLD2*
1.95	0.0093	NM_201380	*PLEC*
1.95	0.0289	NM_005188	*CBL*
1.95	0.0009	NM_001122770	*ZBTB37*
1.95	0.0338	NM_052839	*PANX2*
1.94	0.0070	NM_005839	*SRRM1*

Enrichment over Day 0	T-Test	Access. Number	Gene Symbol
1.94	0.0060	NM_001025236	*TSPAN4*
1.94	0.0086	NM_015175	*NBEAL2*
1.94	0.0377	NM_001270616	*PROX1*
1.94	0.0008	NM_003766	*BECN1*
1.94	0.0283	NM_000336	*SCNN1B*
1.94	0.0489	NM_201222	*MAGED2*
1.94	0.0469	NM_198540	*B3GNT8*
1.94	0.0325	NM_130439	*MXI1*
1.94	0.0211	NM_001040633	*PRKAG2*
1.93	0.0448	NM_015912	*FAM135B*
1.93	0.0021	NM_001166449	*ITIH4*
1.93	0.0442	NM_053282	*SH2D1B*
1.93	0.0360	NM_145279	*MOB3C*
1.93	0.0456	NM_139354	*MATK*
1.93	0.0229	NM_022106	*FAM217B*
1.93	0.0290	NM_004585	*RARRES3*
1.93	0.0398	NM_004404	*SEPT2*
1.93	0.0045	NM_022044	*SDF2L1*
1.92	0.0189	NM_001001671	*MAP3K15*
1.92	0.0136	NM_001099294	*KIAA1644*
1.92	0.0107	NM_001040715	*KIAA0895L*
1.92	0.0418	NM_153209	*KIF19*
1.92	0.0020	NM_007124	*UTRN*
1.92	0.0010	NM_022365	*DNAJC1*
1.92	0.0057	NM_001128165	*FBLN7*
1.91	0.0079	NM_025231	*ZSCAN16*
1.91	0.0216	NM_021183	*RAP2C*
1.91	0.0352	NM_183241	*C9orf142*
1.91	0.0026	NM_022351	*NECAB1*
1.91	0.0012	NM_152629	*GLIS3*
1.91	0.0233	NM_001195007	*PPIE*
1.91	0.0266	NM_013262	*MYLIP*
1.91	0.0067	NM_001136019	*FCGRT*
1.90	0.0493	NM_015201	*BOP1*
1.90	0.0261	NM_032356	*NAA38*
1.90	0.0357	NM_199289	*NEK5*
1.90	0.0011	NM_021247	*PRM3*

Enrichment over Day 0	T-Test	Access. Number	Gene Symbol
1.90	0.0023	NM_001161364	*NPTN*
1.90	0.0089	NM_001113349	*ECE1*
1.89	0.0311	NM_025027	*ZNF606*
1.89	0.0004	NM_002391	*MDK*
1.89	0.0021	NM_004286	*GTPBP1*
1.89	0.0194	NM_022735	*ACBD3*
1.89	0.0222	NM_005980	*S100P*
1.89	0.0264	NM_000890	*KCNJ5*
1.89	0.0021	NM_024958	*NRSN2*
1.89	0.0006	NM_001039848	*GPX4*
1.89	0.0209	NM_018064	*AKIRIN2*
1.89	0.0321	NM_001111019	*NAV2*
1.89	0.0002	NM_003972	*BTAF1*
1.88	0.0054	NM_005806	*OLIG2*
1.88	0.0099	NM_033301	*RPL8*
1.88	0.0004	NM_032875	*FBXL20*
1.88	0.0476	NM_033396	*TNKS1BP1*
1.88	0.0015	NM_001277226	*LGR5*
1.88	0.0038	NM_014161	*MRPL18*
1.88	0.0221	NM_015295	*SMCHD1*
1.87	0.0279	NM_001277115	*DNAH11*
1.87	0.0017	NM_001029882	*AHDC1*
1.87	0.0379	NM_001201429	*CABIN1*
1.87	0.0197	NM_014256	*B3GNT3*
1.87	0.0154	NM_025090	*USP36*
1.87	0.0034	NM_207581	*DUOXA2*
1.87	0.0099	NM_000377	*WAS*
1.87	0.0062	NM_080588	*PTPN7*
1.87	0.0377	NM_018210	*NAXD*
1.87	0.0019	NM_001004058	*OR8K5*
1.87	0.0102	NM_001098725	*TCL1A*
1.87	0.0067	NM_005334	*HCFC1*
1.87	0.0279	NM_001294	*CLPTM1*
1.87	0.0263	NM_004962	*GDF10*
1.86	0.0192	NM_001146288	*AIFM3*
1.86	0.0083	NM_014719	*TCAF1*
1.86	0.0045	NM_001164761	*PRKAR1B*

Enrichment over Day 0	T-Test	Access. Number	Gene Symbol
1.86	0.0239	NM_001256423	*ARHGAP19*
1.86	0.0464	NM_001009877	*BRD9*
1.86	0.0196	NM_001123225	*SYCE3*
1.86	0.0446	NM_001142317	*TXNL4B*
1.86	0.0025	NM_001040107	*HVCN1*
1.86	0.0152	NM_031460	*KCNK17*
1.86	0.0113	NM_138460	*CMTM5*
1.86	0.0128	NM_003969	*UBE2M*
1.86	0.0015	NM_001039617	*ZDHHC19*
1.85	0.0009	NM_015425	*POLR1A*
1.85	0.0091	NM_002135	*NR4A1*
1.85	0.0150	NM_001278650	*B3GALT5*
1.85	0.0173	NM_014874	*MFN2*
1.85	0.0279	NM_001168214	*C3orf80*
1.85	0.0501	NM_001270643	*LUC7L2*
1.85	0.0182	NM_001253815	*SLC52A2*
1.85	0.0446	NM_001042698	*ZSWIM7*
1.85	0.0157	NM_001127608	*FAM189A2*
1.85	0.0017	NM_006018	*HCAR3*
1.85	0.0153	NM_001248006	*TRIM3*
1.84	0.0416	NM_001197128	*IRF3*
1.84	0.0184	NM_014859	*ARHGAP44*
1.84	0.0082	NM_001042474	*ZNF565*
1.84	0.0108	NM_001099733	*ADCYAP1*
1.84	0.0011	NM_152838	*RBM12*
1.84	0.0304	NM_006290	*TNFAIP3*
1.84	0.0317	NM_024956	*TMEM62*
1.84	0.0229	NM_001171934	*CDH23*
1.84	0.0331	NM_031286	*SH3BGRL3*
1.84	0.0044	NM_001172412	*VANGL1*
1.83	0.0483	NM_198042	*PDLIM2*
1.83	0.0011	NM_000369	*TSHR*
1.83	0.0314	NM_005809	*PRDX2*
1.83	0.0224	NM_053002	*MED12L*
1.83	0.0487	NM_000201	*ICAM1*
1.83	0.0024	NM_001009894	*C12orf29*
1.83	0.0103	NM_001619	*GRK2*

Enrichment over Day 0	T-Test	Access. Number	Gene Symbol
1.83	0.0114	NM_001033953	*CALCA*
1.83	0.0168	NM_000626	*CD79B*
1.83	0.0177	NM_139174	*ADAD2*
1.83	0.0020	NM_015210	*MTCL1*
1.83	0.0334	NM_003512	*HIST1H2AC*
1.83	0.0163	NM_001096	*ACLY*
1.82	0.0059	NM_001276469	*B4GALNT1*
1.82	0.0367	NM_002824	*PTMS*
1.82	0.0032	NM_201569	*SMG7*
1.82	0.0319	NM_001145277	*NECAP2*
1.82	0.0199	NM_004390	*CTSH*
1.82	0.0271	NM_002146	*HOXB3*
1.82	0.0054	NM_133265	*AMOT*
1.82	0.0093	NM_207013	*TCEB2*
1.82	0.0008	NM_001143942	*RBM24*
1.82	0.0152	NM_001011548	*MAGEA4*
1.82	0.0382	NM_001062	*TCN1*
1.82	0.0154	NM_032582	*USP32*
1.82	0.0040	NM_033405	*HELZ2*
1.81	0.0328	NM_130832	*OPA1*
1.81	0.0241	NM_001287601	*THAP5*
1.81	0.0084	NM_145233	*ZNF625*
1.81	0.0120	NM_001256279	*ZNF26*
1.81	0.0384	NM_153183	*NUDT10*
1.81	0.0228	NM_014747	*RIMS3*
1.81	0.0312	NM_018440	*PAG1*
1.81	0.0005	NM_005806	*OLIG2*
1.81	0.0373	NM_206909	*PSD3*
1.81	0.0047	NM_001416	*EIF4A1*
1.81	0.0091	NM_001134773	*STX16*
1.81	0.0127	NM_014475	*DHDH*
1.81	0.0387	NM_000117	*EMD*
1.81	0.0073	NM_139202	*MLC1*
1.81	0.0223	NM_001423	*EMP1*
1.81	0.0018	NM_001122772	*AGAP2*
1.80	0.0455	NM_001204077	*UBE4A*
1.80	0.0007	NM_001243610	*LMO3*

Enrichment over Day 0	T-Test	Access. Number	Gene Symbol
1.80	0.0048	NM_007023	*RAPGEF4*
1.80	0.0170	NM_001261400	*PSMD9*
1.80	0.0251	NM_006828	*ASCC3*
1.80	0.0378	NM_001159747	*CC2D2B*
1.80	0.0179	NM_002188	*IL13*
1.80	0.0245	NM_003510	*HIST1H2AK*
1.80	0.0181	NM_198541	*IGFL1*
1.80	0.0345	NM_181847	*AMIGO2*
1.80	0.0077	NM_001032221	*STXBP1*
1.79	0.0113	NM_001008215	*COA5*
1.79	0.0130	NM_020299	*AKR1B10*
1.79	0.0243	NM_014508	*APOBEC3C*
1.79	0.0052	NM_174855	*IDH3B*
1.79	0.0067	NM_004333	*BRAF*
1.79	0.0052	NM_014431	*PALD1*
1.79	0.0339	NM_001242948	*EPDR1*
1.79	0.0159	NM_005912	*MC4R*
1.79	0.0218	NM_018930	*PCDHB10*
1.79	0.0015	NM_012088	*PGLS*
1.79	0.0403	NM_005222	*DLX6*
1.79	0.0284	NM_001233	*CAV2*
1.79	0.0072	NM_001244973	*NRP1*
1.78	0.0034	NM_144673	*CMTM2*
1.78	0.0013	NM_000722	*CACNA2D1*
1.78	0.0004	NM_005932	*MIPEP*
1.78	0.0069	NM_017848	*FAM120C*
1.78	0.0412	NM_212558	*TMEM215*
1.78	0.0290	NM_031419	*NFKBIZ*
1.78	0.0083	NM_152649	*MLKL*
1.78	0.0277	NM_033258	*GNG8*
1.78	0.0131	NM_003413	*ZIC3*
1.78	0.0061	NM_145020	*CFAP53*
1.78	0.0005	NM_015698	*GPKOW*
1.78	0.0209	NM_017857	*SSH3*
1.78	0.0019	NM_018126	*TMEM33*
1.78	0.0295	NM_021974	*POLR2F*
1.78	0.0008	NM_004718	*COX7A2L*

Enrichment over Day 0	T-Test	Access. Number	Gene Symbol
1.78	0.0009	NM_001080379	*PACRG*
1.78	0.0016	NM_002881	*RALB*
1.78	0.0073	NM_004075	*CRY1*
1.78	0.0453	NM_001242804	*LOC100505549*
1.78	0.0272	NM_001076683	*UBTF*
1.78	0.0166	NM_014392	*NSG1*
1.78	0.0306	NM_001171936	*CDH23*
1.77	0.0065	NM_022457	*RFWD2*
1.77	0.0364	NM_032993	*GAR1*
1.77	0.0085	NM_032871	*RELT*
1.77	0.0223	NM_014288	*ITGB3BP*
1.77	0.0047	NM_001163809	*WDR81*
1.77	0.0005	NM_000876	*IGF2R*
1.77	0.0096	NM_020700	*PPM1H*
1.77	0.0392	NM_006456	*ST6GALNAC2*
1.77	0.0404	NM_001166010	*ECI2*
1.77	0.0479	NM_001242750	*LOC100129924*
1.77	0.0007	NM_005570	*LMAN1*
1.77	0.0020	NM_152281	*GORAB*
1.77	0.0012	NM_016194	*GNB5*
1.77	0.0060	NM_001077620	*PRCD*
1.77	0.0245	NM_003947	*KALRN*
1.77	0.0242	NM_001145438	*PGAP2*
1.77	0.0014	NM_020733	*HEG1*
1.77	0.0219	NM_078474	*TM2D3*
1.76	0.0013	NM_001159770	*SLC39A11*
1.76	0.0004	NM_001035004	*KCNIP4*
1.76	0.0469	NM_024512	*LRRC2*
1.76	0.0057	NM_014207	*CD5*
1.76	0.0000	NM_002353	*TACSTD2*
1.76	0.0213	NM_001270661	*MARCH6*
1.76	0.0047	NM_001271856	*GRASP*
1.76	0.0040	NM_019065	*NECAB2*
1.76	0.0274	NM_031461	*CRISPLD1*
1.76	0.0070	NM_033506	*FBXO24*
1.76	0.0488	NM_001162435	*ANKRD66*
1.76	0.0046	NM_032446	*MEGF10*

Enrichment over Day 0	T-Test	Access. Number	Gene Symbol
1.76	0.0039	NM_017914	*C19orf24*
1.76	0.0051	NM_001127183	*CFLAR*
1.76	0.0009	NM_207311	*BICDL1*
1.76	0.0200	NM_001127482	*SPRYD7*
1.76	0.0082	NM_016329	*SFMBT1*
1.76	0.0317	NM_006346	*PIBF1*
1.76	0.0116	NM_024684	*AAMDC*
1.76	0.0393	NM_001145770	*ADGRG1*
1.76	0.0395	NM_000676	*ADORA2B*
1.76	0.0166	NM_002691	*POLD1*
1.76	0.0372	NM_001242560	*MAP4K4*
1.75	0.0194	NM_052846	*EMILIN3*
1.75	0.0119	NM_005745	*BCAP31*
1.75	0.0470	NM_173499	*SPATA8*
1.75	0.0013	NM_001134707	*SARDH*
1.75	0.0006	NM_033290	*MID1*
1.75	0.0006	NM_003653	*COPS3*
1.75	0.0393	NM_005530	*IDH3A*
1.75	0.0369	NM_005603	*ATP8B1*
1.75	0.0031	NM_138964	*PROKR1*
1.75	0.0041	NM_176823	*S100A7A*
1.75	0.0094	NM_001193621	*PINLYP*
1.75	0.0006	NM_020984	*CHAT*
1.75	0.0156	NM_014953	*DIS3*
1.75	0.0293	NM_001282905	*ZFAND4*
1.75	0.0332	NM_020773	*TBC1D14*
1.75	0.0227	NM_000959	*PTGFR*
1.75	0.0328	NM_033554	*HLA-DPA1*
1.75	0.0489	NM_012368	*OR2C1*
1.74	0.0483	NM_001002296	*GOLGA7*
1.74	0.0022	NM_133433	*NIPBL*
1.74	0.0102	NM_001144755	*MYBL1*
1.74	0.0114	NM_015454	*LARP7*
1.74	0.0466	NM_001452	*FOXF2*
1.74	0.0140	NM_199046	*TEPP*
1.74	0.0006	NM_001252090	*MRAS*
1.74	0.0052	NM_001136108	*R3HCC1*

Enrichment over Day 0	T-Test	Access. Number	Gene Symbol
1.74	0.0147	NM_001146029	*SEMA7A*
1.74	0.0210	NM_175741	*NUTM1*
1.74	0.0478	NM_015274	*MAN2B2*
1.74	0.0162	NM_145637	*APOL2*
1.74	0.0220	NM_001031617	*COX19*
1.74	0.0245	NM_001288998	*GMIP*
1.74	0.0451	NM_020069	*ACRV1*
1.74	0.0350	NM_001040192	*DNAJC28*
1.74	0.0364	NM_004481	*GALNT2*
1.74	0.0057	NM_012138	*AATF*
1.74	0.0140	NM_001033602	*MTUS2*
1.74	0.0168	NM_015122	*FCHO1*
1.74	0.0396	NM_173803	*MPV17L*
1.74	0.0104	NM_001271664	*GOLGA6L22*
1.73	0.0296	NM_001174068	*SYTL4*
1.73	0.0123	NM_173553	*TRIML2*
1.73	0.0138	NM_019106	*SEPT3*
1.73	0.0139	NM_003500	*ACOX2*
1.73	0.0419	NM_197974	*BTN3A3*
1.73	0.0406	NM_001286710	*ACSL1*
1.73	0.0022	NM_032424	*MSANTD4*
1.73	0.0012	NM_001146056	*MBOAT7*
1.73	0.0463	NM_012181	*FKBP8*
1.73	0.0020	NM_001287593	*CLIC1*
1.73	0.0376	NM_001122742	*ESR1*
1.73	0.0142	NM_014737	*RASSF2*
1.73	0.0130	NM_001178146	*IGSF10*
1.73	0.0157	NM_017433	*MYO3A*
1.73	0.0114	NM_001142413	*C9orf47*
1.73	0.0170	NM_015364	*LY96*
1.73	0.0014	NM_205768	*ZBTB18*
1.73	0.0362	NM_024809	*TCTN2*
1.72	0.0200	NM_001256088	*C11orf45*
1.72	0.0178	NM_001130991	*HYOU1*
1.72	0.0104	NM_001006939	*LRRC18*
1.72	0.0047	NM_001143680	*GSTK1*
1.72	0.0050	NM_015911	*ZNF691*

Enrichment over Day 0	T-Test	Access. Number	Gene Symbol
1.72	0.0354	NM_175733	*SYT9*
1.72	0.0198	NM_002507	*NGFR*
1.72	0.0043	NM_001290693	*BECN2*
1.72	0.0036	NM_015023	*WDTC1*
1.72	0.0212	NM_014829	*DDX46*
1.72	0.0346	NM_001256613	*HTR3E*
1.72	0.0053	NM_005502	*ABCA1*
1.72	0.0113	NM_001007101	*ZNF484*
1.72	0.0024	NM_003847	*PEX11A*
1.72	0.0386	NM_000093	*COL5A1*
1.72	0.0487	NM_001163149	*ETV1*
1.72	0.0142	NM_014879	*P2RY14*
1.72	0.0091	NM_003016	*SRSF2*
1.72	0.0015	NM_007190	*SEC23IP*
1.72	0.0029	NM_001204126	*LRMP*
1.72	0.0198	NM_001251974	*RCAN2*
1.72	0.0208	NM_018324	*OLAH*
1.72	0.0242	NM_006068	*TLR6*
1.72	0.0481	NM_001204118	*CLEC17A*
1.72	0.0098	NM_153696	*FOLH1B*
1.72	0.0304	NM_153445	*OR5P3*
1.71	0.0001	NM_020242	*KIF15*
1.71	0.0014	NM_015960	*CUTC*
1.71	0.0175	NM_174902	*LDLRAD3*
1.71	0.0005	NM_016279	*CDH9*
1.71	0.0082	NM_001166105	*TADA2A*
1.71	0.0499	NM_002066	*GML*
1.71	0.0279	NM_001282699	*FAM107B*
1.71	0.0305	NM_001127892	*SALL1*
1.71	0.0001	NM_016725	*FOLR1*
1.71	0.0013	NM_001284291	*CBLL1*
1.71	0.0448	NM_152634	*TCEANC*
1.71	0.0011	NM_031420	*MRPL9*
1.71	0.0490	NM_006945	*SPRR2D*
1.71	0.0351	NM_001202431	*PRDX1*
1.71	0.0103	NM_001678	*ATP1B2*
1.71	0.0084	NM_001199380	*RNF145*

Enrichment over Day 0	T-Test	Access. Number	Gene Symbol
1.71	0.0058	NM_015131	*WDR43*
1.71	0.0274	NM_000562	*C8A*
1.71	0.0497	NM_002688	*SEPT5*
1.71	0.0194	NM_006484	*DYRK1B*
1.70	0.0147	NM_017592	*MED29*
1.70	0.0334	NM_001284413	*HACL1*
1.70	0.0051	NM_173549	*ERICH5*
1.70	0.0134	NM_020640	*DCUN1D1*
1.70	0.0444	NM_004642	*CDK2AP1*
1.70	0.0123	NM_015264	*KIAA0930*
1.70	0.0025	NM_003186	*TAGLN*
1.70	0.0032	NM_181842	*ZBTB12*
1.70	0.0069	NM_001287428	*UPP1*
1.70	0.0355	NM_013339	*ALG6*
1.70	0.0140	NM_031284	*ADPGK*
1.70	0.0199	NM_080760	*DACH1*
1.70	0.0424	NM_001037637	*BTF3*
1.70	0.0491	NM_004609	*TCF15*
1.70	0.0030	NM_001137605	*PARVG*
1.70	0.0388	NM_153025	*SPATA33*
1.70	0.0193	NM_001282313	*EXOC7*
1.70	0.0029	NM_138447	*ZNF689*
1.70	0.0154	NM_007357	*COG2*
1.70	0.0246	NM_024837	*ATP8B4*
1.70	0.0476	NM_022461	*AZI2*
1.70	0.0101	NM_014293	*NPTXR*
1.69	0.0106	NM_001259	*CDK6*
1.69	0.0483	NM_173602	*DIP2B*
1.69	0.0125	NM_001130010	*C15orf41*
1.69	0.0052	NM_001195057	*DDIT3*
1.69	0.0023	NM_147134	*NFX1*
1.69	0.0019	NM_001281723	*BTD*
1.69	0.0147	NM_009587	*LGALS9*
1.69	0.0119	NM_001145064	*GATSL2*
1.69	0.0078	NM_001135197	*CCDC36*
1.69	0.0186	NM_003217	*TMBIM6*
1.69	0.0014	NM_001284353	*KIAA1324*

Enrichment over Day 0	T-Test	Access. Number	Gene Symbol
1.69	0.0342	NM_001286837	*RNASEH1*
1.69	0.0030	NM_001123369	*PPP6C*
1.69	0.0371	NM_001166243	*FHIT*
1.69	0.0015	NM_001146186	*PEG3*
1.69	0.0313	NM_001282680	*GAPVD1*
1.69	0.0149	NM_138700	*TRIM40*
1.69	0.0328	NM_000098	*CPT2*
1.69	0.0048	NM_177538	*CYP20A1*
1.69	0.0136	NM_019556	*MOSPD1*
1.69	0.0189	NM_001083620	*GRIA2*
1.69	0.0356	NM_080927	*DCBLD2*
1.69	0.0333	NM_001170820	*IFITM10*
1.69	0.0155	NM_001282567	*ZCCHC17*
1.68	0.0005	NM_144691	*CAPN12*
1.68	0.0407	NM_015015	*KDM4B*
1.68	0.0164	NM_033200	*LMF2*
1.68	0.0325	NM_032404	*TMPRSS3*
1.68	0.0406	NM_183357	*ADCY5*
1.68	0.0194	NM_001040153	*SLAIN1*
1.68	0.0339	NM_032303	*HSDL2*
1.68	0.0224	NM_014886	*NSA2*
1.68	0.0140	NM_003202	*TCF7*
1.68	0.0008	NM_001099271	*POC5*
1.68	0.0263	NM_001184702	*GYG2*
1.68	0.0369	NM_138343	*KLC4*
1.68	0.0281	NM_001145399	*MPPED2*
1.68	0.0233	NM_018953	*HOXC5*
1.68	0.0053	NM_001009905	*B3GNTL1*
1.68	0.0002	NM_003947	*KALRN*
1.68	0.0235	NM_001040630	*NCALD*
1.68	0.0039	NM_152990	*PXT1*
1.68	0.0231	NM_015063	*SLC8A2*
1.68	0.0443	NM_012425	*RSU1*
1.68	0.0360	NM_007178	*STRAP*
1.68	0.0068	NM_031916	*ROPN1L*
1.68	0.0438	NM_203401	*STMN1*
1.68	0.0338	NM_016247	*IMPG2*

Enrichment over Day 0	T-Test	Access. Number	Gene Symbol
1.67	0.0028	NM_181523	*PIK3R1*
1.67	0.0125	NM_001163391	*ZSCAN12*
1.67	0.0041	NM_001098671	*RASGRP2*
1.67	0.0244	NM_080918	*DGUOK*
1.67	0.0411	NM_015705	*SGSM3*
1.67	0.0284	NM_001024382	*HMBS*
1.67	0.0244	NM_001160224	*RNF170*
1.67	0.0030	NM_003052	*SLC34A1*
1.67	0.0349	NM_001270805	*SYT6*
1.67	0.0494	NM_000276	*OCRL*
1.67	0.0369	NM_001201573	*NUBPL*
1.67	0.0450	NM_001256124	*TRIM65*
1.67	0.0029	NM_001007249	*OR8G2*
1.67	0.0013	NM_001172688	*GGA1*
1.67	0.0281	NM_001242377	*DCP2*
1.67	0.0421	NM_006559	*KHDRBS1*
1.66	0.0156	NM_001145639	*GPSM1*
1.66	0.0031	NM_024043	*DBNDD1*
1.66	0.0157	NM_003914	*CCNA1*
1.66	0.0406	NM_177972	*TUB*
1.66	0.0085	NM_001195087	*GGACT*
1.66	0.0002	NM_001013663	*PTRHD1*
1.66	0.0025	NM_007001	*SLC35D2*
1.66	0.0125	NM_020814	*MARCH4*
1.66	0.0336	NM_022658	*HOXC8*
1.66	0.0498	NM_018440	*PAG1*
1.66	0.0389	NM_013362	*ZNF225*
1.66	0.0456	NM_001161566	*TNIK*
1.66	0.0114	NM_006943	*SOX12*
1.66	0.0262	NM_015251	*ATMIN*
1.66	0.0284	NM_016302	*CRBN*
1.66	0.0454	NM_004977	*KCNC3*
1.66	0.0071	NM_033111	*N4BP2L2*
1.66	0.0019	NM_030929	*KAZALD1*
1.66	0.0191	NM_001289021	*KIF6*
1.66	0.0087	NM_130445	*COL18A1*
1.65	0.0035	NM_001282759	*TRA2A*

Enrichment over Day 0	T-Test	Access. Number	Gene Symbol
1.65	0.0409	NM_000271	*NPC1*
1.65	0.0042	NM_013379	*DPP7*
1.65	0.0159	NM_020406	*CD177*
1.65	0.0096	NM_024567	*HMBOX1*
1.65	0.0019	NM_003198	*TCEB3*
1.65	0.0160	NM_032294	*CAMKK1*
1.65	0.0082	NM_003965	*CCRL2*
1.65	0.0430	NM_001128159	*VPS53*
1.65	0.0116	NM_001164712	*AMT*
1.65	0.0028	NM_001134734	*C1orf94*
1.65	0.0207	NM_001037162	*ACOT6*
1.65	0.0038	NM_018951	*HOXA10*
1.65	0.0181	NM_182511	*CBLN2*
1.65	0.0233	NM_032088	*PCDHGA8*
1.65	0.0395	NM_001005181	*OR56B4*
1.65	0.0026	NM_198567	*SIMC1*
1.65	0.0034	NM_001145812	*SH2B1*
1.65	0.0289	NM_001243775	*LIMA1*
1.65	0.0005	NM_001278491	*ANAPC15*
1.65	0.0294	NM_001144000	*AGAP5*
1.65	0.0408	NM_033140	*CALD1*
1.65	0.0500	NM_139058	*ARX*
1.65	0.0146	NM_001042664	*PLEKHG5*
1.65	0.0352	NM_001289050	*SLC22A17*
1.65	0.0008	NM_000446	*PON1*
1.64	0.0065	NM_153228	*ANKFN1*
1.64	0.0128	NM_018431	*DOK5*
1.64	0.0411	NM_001010888	*ZC3H12B*
1.64	0.0094	NM_001077710	*FAM110C*
1.64	0.0047	NM_178329	*CCR3*
1.64	0.0262	NM_033087	*ALG2*
1.64	0.0271	NM_001290020	*ARMC4*
1.64	0.0182	NM_012274	*HYPM*
1.64	0.0030	NM_001278740	*TSPAN6*
1.64	0.0002	NM_014508	*APOBEC3C*
1.64	0.0268	NM_006187	*OAS3*
1.64	0.0263	NM_001161566	*TNIK*

Enrichment over Day 0	T-Test	Access. Number	Gene Symbol
1.64	0.0088	NM_018999	*CCSER2*
1.64	0.0154	NM_024812	*BAALC*
1.64	0.0144	NM_024940	*DOCK5*
1.64	0.0482	NM_001398	*ECH1*
1.64	0.0195	NM_001128143	*PMS1*
1.64	0.0055	NM_005920	*MEF2D*
1.64	0.0242	NM_015075	*IQSEC2*
1.64	0.0206	NM_001042440	*CAST*
1.64	0.0280	NM_001136195	*TNPO2*
1.64	0.0467	NM_001145541	*TCP11L1*
1.64	0.0098	NM_001282950	*COPS7B*
1.64	0.0027	NM_001289934	*LRCH4*
1.64	0.0328	NM_001282360	*ZNF107*
1.63	0.0418	NM_001201428	*GORASP2*
1.63	0.0174	NM_001135099	*TMPRSS2*
1.63	0.0479	NM_001198994	*NADK*
1.63	0.0027	NM_001039762	*FAM196A*
1.63	0.0054	NM_001127692	*PCCA*
1.63	0.0001	NM_198156	*VHL*
1.63	0.0048	NM_001277207	*P2RY6*
1.63	0.0152	NM_173582	*PGM2L1*
1.63	0.0009	NM_201532	*DGKZ*
1.63	0.0052	NM_025191	*EDEM3*
1.63	0.0466	NM_152872	*FAS*
1.63	0.0375	NM_001134665	*TRMT10A*
1.63	0.0184	NM_001252024	*TRPM1*
1.63	0.0003	NM_020710	*LRRC47*
1.63	0.0224	NM_001289021	*KIF6*
1.63	0.0242	NM_025004	*CCDC15*
1.63	0.0430	NM_001039877	*STRN4*
1.63	0.0153	NM_014705	*DOCK4*
1.63	0.0047	NM_014239	*EIF2B2*
1.63	0.0039	NM_007113	*TCHH*
1.63	0.0018	NM_004803	*SLC22A14*
1.63	0.0074	NM_001831	*CLU*
1.63	0.0057	NM_144639	*UROC1*
1.63	0.0041	NM_080491	*GAB2*

Enrichment over Day 0	T-Test	Access. Number	Gene Symbol
1.63	0.0006	NM_017526	*LEPROT*
1.63	0.0310	NM_001949	*E2F3*
1.63	0.0073	NM_181746	*CERS2*
1.63	0.0241	NM_012229	*NT5C2*
1.63	0.0082	NM_001080508	*TBX18*
1.62	0.0055	NM_023929	*ZBTB10*
1.62	0.0014	NM_001256676	*STOML1*
1.62	0.0307	NM_006699	*MAN1A2*
1.62	0.0003	NM_001199319	*PEX26*
1.62	0.0093	NM_144590	*ANKRD22*
1.62	0.0097	NM_001736	*C5AR1*
1.62	0.0135	NM_004937	*CTNS*
1.62	0.0137	NM_001142587	*NFYC*
1.62	0.0034	NM_016733	*LIMK2*
1.62	0.0010	NM_152688	*KHDRBS2*
1.62	0.0061	NM_182538	*SPNS3*
1.62	0.0073	NM_021873	*CDC25B*
1.62	0.0453	NM_138356	*SHF*
1.62	0.0096	NM_001258020	*GRIA1*
1.62	0.0047	NM_147191	*MMP21*
1.62	0.0200	NM_001077350	*NPRL3*
1.62	0.0053	NM_001286582	*PHRF1*
1.62	0.0030	NM_130435	*PTPRE*
1.62	0.0197	NM_000671	*ADH5*
1.62	0.0038	NM_014720	*SLK*
1.62	0.0380	NM_015409	*EP400*
1.62	0.0008	NM_004330	*BNIP2*
1.62	0.0486	NM_001289132	*C15orf57*
1.62	0.0197	NM_001258214	*IL12RB2*
1.62	0.0032	NM_181846	*ZSCAN22*
1.62	0.0012	NM_001288804	*HEPACAM2*
1.62	0.0000	NM_017881	*NMRK1*
1.62	0.0142	NM_015332	*NUDCD3*
1.62	0.0053	NM_014338	*PISD*
1.62	0.0113	NM_001204477	*CDRT4*
1.62	0.0128	NM_139057	*ADAMTS17*
1.61	0.0231	NM_001161819	*MYO1B*

Enrichment over Day 0	T-Test	Access. Number	Gene Symbol
1.61	0.0077	NM_183419	*RNF19A*
1.61	0.0407	NM_001080471	*PEAR1*
1.61	0.0325	NM_002737	*PRKCA*
1.61	0.0385	NM_001145099	*SLC2A6*
1.61	0.0015	NM_002267	*KPNA3*
1.61	0.0334	NM_005873	*RGS19*
1.61	0.0443	NM_001142621	*TGFBRAP1*
1.61	0.0022	NM_004598	*SPOCK1*
1.61	0.0011	NM_001146114	*DIP2A*
1.61	0.0268	NM_001171868	*TMEM200B*
1.61	0.0176	NM_001004751	*OR51D1*
1.61	0.0282	NM_001165036	*OGDH*
1.61	0.0052	NM_001098831	*MORN4*
1.61	0.0189	NM_003458	*BSN*
1.61	0.0004	NM_025216	*WNT10A*
1.61	0.0488	NM_001081675	*KLHL38*
1.61	0.0292	NM_004045	*ATOX1*
1.61	0.0347	NM_021920	*SCT*
1.61	0.0291	NM_134261	*RORA*
1.61	0.0154	NM_005188	*CBL*
1.61	0.0417	NM_172060	*EYA1*
1.61	0.0361	NM_001288967	*CEP70*
1.61	0.0126	NM_000395	*CSF2RB*
1.61	0.0092	NM_002300	*LDHB*
1.61	0.0352	NM_018030	*OSBPL1A*
1.61	0.0437	NM_001002254	*AWAT2*
1.61	0.0149	NM_001011655	*TMEM44*
1.61	0.0227	NM_000390	*CHM*
1.60	0.0010	NM_003728	*UNC5C*
1.60	0.0094	NM_001122965	*RPTN*
1.60	0.0126	NM_178434	*LCE3C*
1.60	0.0332	NM_018939	*PCDHB6*
1.60	0.0336	NM_012353	*OR1C1*
1.60	0.0306	NM_182577	*ODF3L2*
1.60	0.0334	NM_198585	*ENTPD8*
1.60	0.0076	NM_005980	*S100P*
1.60	0.0382	NM_004767	*GPR37L1*

Enrichment over Day 0	T-Test	Access. Number	Gene Symbol
1.60	0.0240	NM_145274	*TMEM99*
1.60	0.0325	NM_018665	*DDX43*
1.60	0.0025	NM_001270943	*KLF7*
1.60	0.0038	NM_001184971	*PACSIN2*
1.60	0.0021	NM_002929	*GRK1*
1.60	0.0483	NM_144602	*C16orf78*
1.60	0.0279	NM_007021	*C10orf10*
1.60	0.0269	NM_001428	*ENO1*
1.60	0.0064	NM_001145157	*NR2F2*
1.60	0.0376	NM_001282430	*LBX2*
1.60	0.0017	NM_001204192	*TP73*
1.60	0.0227	NM_001004452	*OR1J4*
1.60	0.0049	NM_006158	*NEFL*
1.60	0.0436	NM_138465	*GLI4*
1.60	0.0384	NM_014586	*HUNK*
1.60	0.0002	NM_015338	*ASXL1*
1.60	0.0367	NM_001170795	*ATRAID*
1.60	0.0187	NM_002232	*KCNA3*
1.60	0.0144	NM_173614	*NOMO2*
1.60	0.0164	NM_001193635	*DHRS4L2*
1.60	0.0229	NM_001040451	*RUFY1*
1.60	0.0274	NM_000945	*PPP3R1*
1.60	0.0255	NM_001003827	*TRIM34*
1.60	0.0136	NM_016309	*LCMT1*
1.60	0.0146	NM_014673	*EMC2*
1.60	0.0285	NM_030770	*TMPRSS5*
1.60	0.0010	NM_017970	*NRDE2*
1.60	0.0070	NM_018013	*SOBP*
1.60	0.0265	NM_001169154	*PIH1D3*
1.60	0.0155	NM_182981	*OSGIN1*
1.60	0.0294	NM_001031713	*MCUR1*
1.59	0.0037	NM_001039613	*IAH1*
1.59	0.0387	NM_001099625	*MTFR1L*
1.59	0.0434	NM_173211	*TGIF1*
1.59	0.0297	NM_001407	*CELSR3*
1.59	0.0096	NM_017838	*NHP2*
1.59	0.0241	NM_001037663	*EEF1B2*

Enrichment over Day 0	T-Test	Access. Number	Gene Symbol
1.59	0.0154	NM_031965	*GSG2*
1.59	0.0104	NM_206900	*RTN2*
1.59	0.0016	NM_003071	*HLTF*
1.59	0.0312	NM_001287008	*SUSD3*
1.59	0.0433	NM_007068	*DMC1*
1.59	0.0097	NM_001243877	*TOB1*
1.59	0.0352	NM_001242338	*ATAD2B*
1.59	0.0034	NM_001164356	*THAP4*
1.59	0.0057	NM_001171110	*FAM133A*
1.59	0.0008	NM_001291454	*ATP2C2*
1.59	0.0044	NM_173465	*COL23A1*
1.59	0.0105	NM_005642	*TAF7*
1.59	0.0078	NM_001875	*CPS1*
1.59	0.0040	NM_003317	*NKX2-1*
1.59	0.0247	NM_022913	*GPBP1*
1.59	0.0175	NM_003239	*TGFB3*
1.59	0.0357	NM_001166108	*PALLD*
1.59	0.0003	NM_052946	*NOSTRIN*
1.59	0.0034	NM_001129995	*KCTD15*
1.59	0.0400	NM_001001662	*ZNF782*
1.58	0.0007	NM_001287748	*FDFT1*
1.58	0.0329	NM_000097	*CPOX*
1.58	0.0501	NM_001004492	*OR2B11*
1.58	0.0217	NM_001080435	*WHAMM*
1.58	0.0017	NM_015629	*PRPF31*
1.58	0.0158	NM_018412	*ST7*
1.58	0.0361	NM_053003	*SIGLEC12*
1.58	0.0177	NM_012255	*XRN2*
1.58	0.0233	NM_001144984	*CCDC169*
1.58	0.0129	NM_194312	*ESPNL*
1.58	0.0322	NM_001134836	*SIRPB2*
1.58	0.0233	NM_016356	*DCDC2*
1.58	0.0024	NM_024784	*ZBTB3*
1.58	0.0495	NM_001195386	*TMEM99*
1.58	0.0002	NM_033135	*PDGFD*
1.58	0.0434	NM_020203	*MEPE*
1.58	0.0086	NM_014497	*ZNF638*

Enrichment over Day 0	T-Test	Access. Number	Gene Symbol
1.58	0.0169	NM_003920	*TIMELESS*
1.58	0.0088	NM_001242898	*PPP6R2*
1.58	0.0146	NM_001195202	*LOC100129636*
1.58	0.0413	NM_052924	*RHPN1*
1.58	0.0217	NM_138496	*CYHR1*
1.58	0.0303	NM_181482	*LDLRAD4*
1.58	0.0097	NM_181581	*DUS4L*
1.58	0.0116	NM_181787	*DPY19L4*
1.58	0.0107	NM_015898	*ZBTB7A*
1.58	0.0018	NM_020461	*TUBGCP6*
1.58	0.0103	NM_013943	*CLIC4*
1.58	0.0319	NM_001164372	*GPN3*
1.58	0.0170	NM_001270395	*PKIB*
1.58	0.0034	NM_203288	*RP9*
1.58	0.0297	NM_001160130	*KCNQ5*
1.58	0.0237	NM_033405	*HELZ2*
1.58	0.0119	NM_015305	*ANGEL1*
1.58	0.0399	NM_012455	*PSD4*
1.57	0.0217	NM_001010879	*ZIK1*
1.57	0.0096	NM_004435	*ENDOG*
1.57	0.0269	NM_183008	*UBXN11*
1.57	0.0423	NM_005612	*REST*
1.57	0.0028	NM_001171936	*CDH23*
1.57	0.0176	NM_001101677	*SOHLH1*
1.57	0.0073	NM_032024	*C10orf11*
1.57	0.0027	NM_004885	*NPFFR2*
1.57	0.0251	NM_001204367	*MGST2*
1.57	0.0319	NM_004572	*PKP2*
1.57	0.0228	NM_003248	*THBS4*
1.57	0.0210	NM_018290	*PGM2*
1.57	0.0218	NM_001136116	*ZNF879*
1.57	0.0479	NM_001168378	*ZIC4*
1.57	0.0069	NM_001031738	*TMEM150A*
1.57	0.0495	NM_006785	*MALT1*
1.57	0.0246	NM_005433	*YES1*
1.57	0.0040	NM_001199096	*BAIAP3*
1.57	0.0161	NM_080426	*GNAS*

Enrichment over Day 0	T-Test	Access. Number	Gene Symbol
1.57	0.0300	NM_016131	*RAB10*
1.57	0.0071	NM_000597	*IGFBP2*
1.57	0.0004	NM_001077654	*TNFAIP8*
1.57	0.0210	NM_002826	*QSOX1*
1.57	0.0358	NM_014347	*ZNF324*
1.57	0.0056	NM_001136026	*SEC13*
1.57	0.0001	NM_178270	*ATG4A*
1.57	0.0336	NM_178518	*TMEM102*
1.56	0.0069	NM_003407	*ZFP36*
1.56	0.0101	NM_024855	*ACTR5*
1.56	0.0443	NM_001135051	*FAM160B1*
1.56	0.0450	NM_014410	*CLUL1*
1.56	0.0008	NM_207113	*SLC37A3*
1.56	0.0144	NM_001256172	*ZNF85*
1.56	0.0272	NM_153437	*ODF2*
1.56	0.0376	NM_015013	*KDM1A*
1.56	0.0007	NM_000665	*ACHE*
1.56	0.0228	NM_001290137	*HAGHL*
1.56	0.0426	NM_002910	*RENBP*
1.56	0.0128	NM_001270411	*APOBEC3B*
1.56	0.0192	NM_015038	*KIAA0754*
1.56	0.0019	NM_002833	*PTPN9*
1.56	0.0014	NM_033316	*MELTF*
1.56	0.0183	NM_022896	*LPIN3*
1.56	0.0195	NM_001103184	*FMN1*
1.56	0.0090	NM_022358	*KCNK15*
1.56	0.0284	NM_201286	*USP51*
1.56	0.0102	NM_021071	*ART4*
1.56	0.0145	NM_001190947	*TRAF1*
1.56	0.0475	NM_205837	*LST1*
1.56	0.0010	NM_001243740	*ZNF124*
1.56	0.0469	NM_170784	*MKKS*
1.56	0.0009	NM_001288953	*TTC7A*
1.56	0.0038	NM_024885	*TAF7L*
1.56	0.0256	NM_018071	*ARHGEF40*
1.56	0.0092	NM_001130677	*C17orf96*
1.56	0.0260	NM_001289807	*CRYAB*

Enrichment over Day 0	T-Test	Access. Number	Gene Symbol
1.56	0.0445	NM_001242809	*ANKRD6*
1.56	0.0182	NM_002431	*MNAT1*
1.56	0.0197	NM_005971	*FXYD3*
1.56	0.0005	NM_001146009	*SLCO5A1*
1.56	0.0001	NM_001243743	*FANCC*
1.56	0.0477	NM_153182	*MINA*
1.56	0.0212	NM_175617	*MT1E*
1.56	0.0008	NM_001276501	*GPSM3*
1.56	0.0238	NM_001145137	*CPT1B*
1.56	0.0377	NM_003042	*SLC6A1*
1.56	0.0004	NM_033204	*ZNF101*
1.56	0.0047	NM_004001	*FCGR2B*
1.56	0.0039	NM_002388	*MCM3*
1.56	0.0071	NM_001033560	*DYX1C1*
1.56	0.0049	NM_182642	*CTDSP1*
1.56	0.0008	NM_001278500	*ADORA2A*
1.56	0.0208	NM_138459	*NUS1*
1.56	0.0285	NM_139126	*PPIL4*
1.56	0.0089	NM_173199	*NR4A3*
1.55	0.0021	NM_001146192	*ZMYND12*
1.55	0.0007	NM_014981	*MYH15*
1.55	0.0141	NM_032799	*ZDHHC12*
1.55	0.0040	NM_001031712	*TRMT11*
1.55	0.0011	NM_000594	*TNF*
1.55	0.0144	NM_003883	*HDAC3*
1.55	0.0359	NM_000914	*OPRM1*
1.55	0.0161	NM_001201377	*ALDH7A1*
1.55	0.0344	NM_174905	*FAM98C*
1.55	0.0032	NM_001136197	*FZR1*
1.55	0.0009	NM_005545	*ISLR*
1.55	0.0382	NM_014298	*QPRT*
1.55	0.0199	NM_001102402	*PCTP*
1.55	0.0366	NM_021777	*ADAM28*
1.55	0.0057	NM_004541	*NDUFA1*
1.55	0.0102	NM_006005	*WFS1*
1.55	0.0200	NM_006120	*HLA-DMA*
1.55	0.0315	NM_080821	*FAM210B*

Enrichment over Day 0	T-Test	Access. Number	Gene Symbol
1.55	0.0225	NM_152353	*CLDND2*
1.55	0.0400	NM_001142610	*ULK2*
1.55	0.0000	NM_012310	*KIF4A*
1.55	0.0171	NM_018302	*C4orf19*
1.55	0.0420	NM_001281439	*SMAP1*
1.55	0.0131	NM_138346	*KIAA2013*
1.55	0.0349	NM_001134473	*GSE1*
1.55	0.0367	NM_130901	*OTUD7A*
1.55	0.0232	NM_207037	*TCF12*
1.55	0.0495	NM_005425	*TNP2*
1.55	0.0187	NM_001167990	*PQBP1*
1.55	0.0403	NM_015150	*RFTN1*
1.55	0.0351	NM_199482	*MOB4*
1.55	0.0288	NM_021222	*PRUNE1*
1.55	0.0166	NM_000392	*ABCC2*
1.55	0.0236	NM_001199174	*MLST8*
1.55	0.0078	NM_015575	*GIGYF2*
1.55	0.0227	NM_020924	*ZBTB26*
1.55	0.0459	NM_032296	*FLYWCH1*
1.55	0.0063	NM_006771	*KRT38*
1.55	0.0070	NM_032636	*PSRC1*
1.55	0.0029	NM_001009894	*C12orf29*
1.55	0.0355	NM_001286554	*USP49*
1.54	0.0026	NM_153325	*DEFB125*
1.54	0.0010	NM_021246	*LY6G6D*
1.54	0.0061	NM_001017523	*BTBD11*
1.54	0.0012	NM_007031	*HSF2BP*
1.54	0.0017	NM_001009613	*SPANXN4*
1.54	0.0050	NM_004587	*RRBP1*
1.54	0.0047	NM_031423	*NUF2*
1.54	0.0002	NM_005667	*RNF103*
1.54	0.0288	NM_198239	*WISP3*
1.54	0.0276	NM_033540	*MFN1*
1.54	0.0342	NM_015506	*MMACHC*
1.54	0.0102	NM_006540	*NCOA2*
1.54	0.0379	NM_003815	*ADAM15*
1.54	0.0394	NM_000906	*NPR1*

Enrichment over Day 0	T-Test	Access. Number	Gene Symbol
1.54	0.0250	NM_002973	*ATXN2*
1.54	0.0024	NM_017425	*SPA17*
1.54	0.0454	NM_001171936	*CDH23*
1.54	0.0264	NM_024623	*OGFOD2*
1.54	0.0002	NM_014932	*NLGN1*
1.54	0.0337	NM_001136506	*SLC22A24*
1.54	0.0035	NM_014437	*SLC39A1*
1.54	0.0398	NM_198531	*ATP9B*
1.54	0.0051	NM_020194	*MFF*
1.54	0.0307	NM_001080435	*WHAMM*
1.54	0.0152	NM_014034	*ASF1A*
1.54	0.0073	NM_001253724	*DPYSL5*
1.54	0.0063	NM_145047	*OSCP1*
1.54	0.0493	NM_020374	*C12orf4*
1.54	0.0162	NM_001111309	*PDE4A*
1.54	0.0275	NM_001199621	*NCOA7*
1.54	0.0472	NM_001201573	*NUBPL*
1.54	0.0272	NM_002518	*NPAS2*
1.54	0.0012	NM_005193	*CDX4*
1.54	0.0253	NM_019069	*WDR5B*
1.54	0.0361	NM_014902	*DLGAP4*
1.54	0.0213	NM_153633	*HOXC4*
1.54	0.0244	NM_014373	*GPR160*
1.54	0.0053	NM_001828	*CLC*
1.54	0.0029	NM_001083607	*PTCH1*
1.54	0.0122	NM_001105556	*THEMIS2*
1.54	0.0051	NM_024082	*PRRG3*
1.54	0.0039	NM_001287001	*ST6GALNAC6*
1.53	0.0087	NM_152857	*WTAP*
1.53	0.0012	NM_017416	*IL1RAPL2*
1.53	0.0037	NM_000495	*COL4A5*
1.53	0.0105	NM_203504	*G3BP2*
1.53	0.0394	NM_001284207	*DCAF5*
1.53	0.0410	NM_001291543	*CT45A7*
1.53	0.0191	NM_001098722	*GNG4*
1.53	0.0286	NM_001010905	*C6orf58*
1.53	0.0007	NM_052896	*CSMD2*

Enrichment over Day 0	T-Test	Access. Number	Gene Symbol
1.53	0.0472	NM_014752	*SPCS2*
1.53	0.0172	NM_006817	*ERP29*
1.53	0.0196	NM_001257305	*KRTAP1-4*
1.53	0.0198	NM_002493	*NDUFB6*
1.53	0.0262	NM_001008895	*CUL4A*
1.53	0.0084	NM_004938	*DAPK1*
1.53	0.0172	NM_170662	*CBLB*
1.53	0.0132	NM_005116	*SLC23A2*
1.53	0.0254	NM_001134650	*EIF4E3*
1.53	0.0179	NM_001202474	*PGR*
1.53	0.0253	NM_032124	*HDHD2*
1.53	0.0146	NM_032207	*C19orf44*
1.53	0.0278	NM_213560	*PKN1*
1.53	0.0227	NM_012153	*EHF*
1.53	0.0489	NM_031417	*MARK4*
1.53	0.0365	NM_001242575	*LOC100130451*
1.53	0.0386	NM_002693	*POLG*
1.53	0.0066	NM_013328	*PYCR2*
1.53	0.0366	NM_033113	*ZNF628*
1.53	0.0164	NM_001783	*CD79A*
1.53	0.0452	NM_001145006	*MUC7*
1.53	0.0039	NM_031913	*ESYT3*
1.53	0.0230	NM_006570	*RRAGA*
1.53	0.0173	NM_015203	*RPRD2*
1.53	0.0068	NM_001204744	*CDH16*
1.53	0.0208	NM_021217	*ZNF77*
1.53	0.0049	NM_206824	*VKORC1*
1.53	0.0110	NM_014616	*ATP11B*
1.53	0.0272	NM_017559	*FNDC8*
1.53	0.0146	NM_024873	*TNIP3*
1.53	0.0075	NM_015470	*RAB11FIP5*
1.53	0.0175	NM_006694	*JTB*
1.53	0.0393	NM_001136504	*SYT2*
1.53	0.0207	NM_001267048	*KIAA1324*
1.53	0.0123	NM_001282587	*H6PD*
1.53	0.0053	NM_001167912	*VEPH1*
1.53	0.0070	NM_001161575	*LRRC36*

Enrichment over Day 0	T-Test	Access. Number	Gene Symbol
1.52	0.0023	NM_032726	*PLCD4*
1.52	0.0149	NM_213656	*KRT39*
1.52	0.0138	NM_001161834	*C7orf72*
1.52	0.0411	NM_001278063	*SKOR2*
1.52	0.0255	NM_005528	*DNAJC4*
1.52	0.0464	NM_175734	*C17orf74*
1.52	0.0041	NM_001190447	*PPP2R3A*
1.52	0.0050	NM_025147	*COQ10B*
1.52	0.0161	NM_006284	*TAF10*
1.52	0.0007	NM_001136155	*ERG*
1.52	0.0087	NM_001024212	*S100A13*
1.52	0.0056	NM_001190182	*CCDC17*
1.52	0.0060	NM_178135	*HSD17B13*
1.52	0.0094	NM_017633	*FAM46A*
1.52	0.0043	NM_018003	*UACA*
1.52	0.0090	NM_152832	*FAM89B*
1.52	0.0468	NM_022486	*SUSD1*
1.52	0.0049	NM_004052	*BNIP3*
1.52	0.0007	NM_001167608	*RHBDD1*
1.52	0.0227	NM_017924	*C14orf119*
1.52	0.0415	NM_080720	*H2AFB3*
1.52	0.0028	NM_006979	*SLC39A7*
1.52	0.0226	NM_001130514	*ERICH4*
1.52	0.0317	NM_016161	*A4GNT*
1.52	0.0167	NM_015043	*TBC1D9B*
1.52	0.0019	NM_175882	*SPPL2C*
1.52	0.0016	NM_001007523	*F8A2*
1.52	0.0095	NM_001321	*CSRP2*
1.52	0.0179	NM_053004	*GNB1L*
1.52	0.0188	NM_020794	*LRRC7*
1.52	0.0117	NM_014364	*GAPDHS*
1.52	0.0134	NM_001253791	*SMIM22*
1.52	0.0275	NM_005839	*SRRM1*
1.52	0.0310	NM_021632	*ZNF350*
1.52	0.0097	NM_006040	*HS3ST4*
1.52	0.0486	NM_198273	*LYSMD3*
1.52	0.0248	NM_001127219	*ACCS*

Enrichment over Day 0	T-Test	Access. Number	Gene Symbol
1.52	0.0441	NM_015271	*TRIM2*
1.52	0.0309	NM_004651	*USP11*
1.52	0.0043	NM_014248	*RBX1*
1.52	0.0346	NM_152325	*TEX26*
1.51	0.0420	NM_001139498	*FGF13*
1.51	0.0392	NM_033180	*OR51B2*
1.51	0.0049	NM_003588	*CUL4B*
1.51	0.0240	NM_152460	*C17orf77*
1.51	0.0323	NM_019601	*SUSD2*
1.51	0.0320	NM_173635	*SIGLECL1*
1.51	0.0259	NM_174983	*MFSD12*
1.51	0.0116	NM_001167670	*TMEM239*
1.51	0.0163	NM_002743	*PRKCSH*
1.51	0.0234	NM_001906	*CTRB1*
1.51	0.0361	NM_017956	*TRMT12*
1.51	0.0117	NM_003430	*ZNF91*
1.51	0.0066	NM_001013653	*LRRC26*
1.51	0.0278	NM_033446	*MVB12B*
1.51	0.0135	NM_002612	*PDK4*
1.51	0.0142	NM_001284269	*EFCAB11*
1.51	0.0291	NM_002751	*MAPK11*
1.51	0.0128	NM_138331	*RNASE8*
1.51	0.0009	NM_139319	*SLC17A8*
1.51	0.0046	NM_032306	*ALKBH7*
1.51	0.0363	NM_006311	*NCOR1*
1.51	0.0447	NM_015395	*TECPR1*
1.51	0.0226	NM_003126	*SPTA1*
1.51	0.0016	NM_004820	*CYP7B1*
1.51	0.0360	NM_080872	*UNC5D*
1.51	0.0389	NM_001142482	*NREP*
1.51	0.0499	NM_006539	*CACNG3*
1.51	0.0499	NM_001164104	*MOV10L1*
1.51	0.0289	NM_001146109	*PTGR1*
1.51	0.0008	NM_004610	*TCP10*
1.51	0.0027	NM_023002	*HAPLN4*
1.51	0.0003	NM_001042581	*SNUPN*
1.51	0.0070	NM_001037340	*PDE4B*

Enrichment over Day 0	T-Test	Access. Number	Gene Symbol
1.51	0.0502	NM_080627	*SOGA1*
1.51	0.0288	NM_001278098	*GDNF*
1.51	0.0166	NM_001126336	*VCAN*
1.51	0.0396	NM_001287049	*SAXO1*
1.51	0.0070	NM_003145	*SSR2*
1.50	0.0232	NM_007110	*TEP1*
1.50	0.0031	NM_004619	*TRAF5*
1.50	0.0458	NM_003794	*SNX4*
1.50	0.0060	NM_001004464	*OR10G8*
1.50	0.0121	NM_001171909	*CXorf40A*
1.50	0.0187	NM_014222	*NDUFA8*
1.50	0.0032	NM_001136530	*SERPINE2*
1.50	0.0019	NM_001081675	*KLHL38*
1.50	0.0128	NM_000862	*HSD3B1*
1.50	0.0462	NM_001127180	*MYO7A*
1.50	0.0373	NM_022464	*SIL1*
1.50	0.0097	NM_001114635	*PLAG1*
1.50	0.0189	NM_001134888	*RTL1*
1.50	0.0171	NM_001033521	*CSTF1*
1.50	0.0155	NM_006454	*MXD4*
1.50	0.0220	NM_001134771	*SLC12A5*
1.50	0.0233	NM_001286756	*ANAPC4*
1.50	0.0016	NM_001166426	*WDR13*
1.50	0.0384	NM_144635	*FAM131A*
1.50	0.0418	NM_031883	*PCDHAC2*
1.50	0.0015	NM_001004450	*OR1B1*
1.50	0.0314	NM_207411	*XKR5*
1.50	0.0484	NM_002277	*KRT31*
1.50	0.0215	NM_006240	*PPEF1*
1.50	0.0035	NM_001077693	*ECSCR*
1.50	0.0248	NM_018998	*FBXW5*
1.50	0.0411	NM_001031804	*MAF*
1.50	0.0056	NM_014383	*ZBTB32*
1.50	0.0222	NM_015322	*FEM1B*
1.50	0.0188	NM_001083601	*NAA60*
1.50	0.0001	NM_033011	*PLAT*
1.50	0.0085	NM_001143841	*TMEM106C*

Enrichment over Day 0	T-Test	Access. Number	Gene Symbol
1.50	0.0177	NM_001278790	*GORASP1*
1.50	0.0277	NM_002003	*FCN1*
1.50	0.0231	NM_020733	*HEG1*
1.50	0.0207	NM_173511	*FAM117B*
1.50	0.0466	NM_007014	*WWP2*
1.50	0.0129	NM_018132	*CENPQ*
1.50	0.0187	NM_025049	*PIF1*
1.50	0.0415	NM_001080453	*INTS1*
1.50	0.0168	NM_031488	*L3MBTL2*
1.50	0.0013	NM_001174122	*ZFYVE27*
1.50	0.0046	NM_001170535	*ATAD3A*
1.50	0.0091	NM_016613	*FAM198B*
1.50	0.0148	NM_001127323	*GRM8*
1.50	0.0240	NM_015062	*PPRC1*
1.50	0.0400	NM_001720	*BMP8B*

Supplemental Table 2. Genes with at least 2 sgRNAs enriched ≧1.5-fold after T cell killing compared to Day 0 reference control, p <0.05

Enrichment over Day 0	T-Test	Access. Number	Gene Symbol
8.13	0.0123	NM_152629	*GLIS3*
6.31	0.0159	NM_001143680	*GSTK1*
5.76	0.0032	NM_001803	*CD52*
5.11	0.0413	NM_001030288	*SPN*
5.10	0.0496	NM_001030288	*SPN*
4.96	0.0002	NM_173598	*KSR2*
4.44	0.0063	NM_001803	*CD52*
3.41	0.0005	NM_005603	*ATP8B1*
3.21	0.0108	NM_001110354	*ZP3*
3.01	0.0140	NM_002135	*NR4A1*
2.97	0.0039	NM_001270616	*PROX1*
2.65	0.0314	NM_001005855	*ATP8B2*
2.60	0.0033	NM_006290	*TNFAIP3*
2.54	0.0020	NM_021960	*MCL1*
2.52	0.0131	NM_033554	*HLA-DPA1*
2.40	0.0357	NM_001110354	*ZP3*
2.35	0.0260	NM_001276469	*B4GALNT1*
2.26	0.0356	NM_183419	*RNF19A*
2.16	0.0414	NM_001039877	*STRN4*
2.11	0.0055	NM_021960	*MCL1*
2.05	0.0344	NM_173598	*KSR2*
2.05	0.0034	NM_133433	*NIPBL*
2.03	0.0476	NM_001407	*CELSR3*
2.01	0.0285	NM_030765	*B3GNT4*
1.98	0.0004	NM_002507	*NGFR*
1.97	0.0005	NM_030765	*B3GNT4*
1.96	0.0044	NM_001005855	*ATP8B2*
1.96	0.0025	NM_001284269	*EFCAB11*
1.95	0.0289	NM_005188	*CBL*
1.94	0.0070	NM_005839	*SRRM1*
1.94	0.0377	NM_001270616	*PROX1*
1.91	0.0012	NM_152629	*GLIS3*
1.89	0.0222	NM_005980	*S100P*
1.88	0.0054	NM_005806	*OLIG2*

Enrichment over Day 0	T-Test	Access. Number	Gene Symbol
1.85	0.0091	NM_002135	*NR4A1*
1.84	0.0304	NM_006290	*TNFAIP3*
1.83	0.0024	NM_001009894	*C12orf29*
1.82	0.0059	NM_001276469	*B4GALNT1*
1.82	0.0040	NM_033405	*HELZ2*
1.81	0.0312	NM_018440	*PAG1*
1.81	0.0005	NM_005806	*OLIG2*
1.79	0.0243	NM_014508	*APOBEC3C*
1.78	0.0306	NM_001171936	*CDH23*
1.77	0.0245	NM_003947	*KALRN*
1.77	0.0014	NM_020733	*HEG1*
1.75	0.0369	NM_005603	*ATP8B1*
1.75	0.0328	NM_033554	*HLA-DPA1*
1.74	0.0022	NM_133433	*NIPBL*
1.72	0.0047	NM_001143680	*GSTK1*
1.72	0.0198	NM_002507	*NGFR*
1.68	0.0002	NM_003947	*KALRN*
1.67	0.0369	NM_001201573	*NUBPL*
1.66	0.0498	NM_018440	*PAG1*
1.66	0.0456	NM_001161566	*TNIK*
1.66	0.0191	NM_001289021	*KIF6*
1.64	0.0002	NM_014508	*APOBEC3C*
1.64	0.0263	NM_001161566	*TNIK*
1.63	0.0224	NM_001289021	*KIF6*
1.63	0.0430	NM_001039877	*STRN4*
1.61	0.0077	NM_183419	*RNF19A*
1.61	0.0488	NM_001081675	*KLHL38*
1.61	0.0154	NM_005188	*CBL*
1.60	0.0076	NM_005980	*S100P*
1.59	0.0297	NM_001407	*CELSR3*
1.58	0.0217	NM_001080435	*WHAMM*
1.58	0.0237	NM_033405	*HELZ2*
1.57	0.0028	NM_001171936	*CDH23*
1.55	0.0029	NM_001009894	*C12orf29*
1.54	0.0454	NM_001171936	*CDH23*
1.54	0.0307	NM_001080435	*WHAMM*
1.54	0.0472	NM_001201573	*NUBPL*

Enrichment over Day 0	T-Test	Access. Number	Gene Symbol
1.52	0.0275	NM_005839	*SRRM1*
1.51	0.0142	NM_001284269	*EFCAB11*
1.50	0.0019	NM_001081675	*KLHL38*
1.50	0.0231	NM_020733	*HEG1*

Supplemental Table 3. sgRNAs enriched ≧ 1.5-fold after 10 population doublings compared to Day 0 reference control, $p < 0.05$

Enrichment over Day 0	T-Test	Access. Number	Gene Symbol
4.00	0.0137	NM_001204294	*MUC1*
3.83	0.0237	NM_001172697	*TSFM*
3.51	0.0177	NM_001204294	*MUC1*
3.35	0.0050	NM_001144925	*MX1*
3.24	0.0119	NM_177454	*FAM171B*
2.91	0.0126	NM_018103	*LRRC8D*
2.76	0.0001	NM_145109	*MAP2K3*
2.74	0.0418	NM_001079881	*PRKD2*
2.70	0.0132	NM_006577	*B3GNT2*
2.66	0.0115	NM_001803	*CD52*
2.58	0.0035	NM_053277	*CLIC6*
2.54	0.0028	NM_153682	*PIGP*
2.53	0.0235	NM_001010909	*MUC21*
2.53	0.0273	NM_080552	*SLC32A1*
2.51	0.0108	NM_020851	*ISLR2*
2.43	0.0222	NM_001803	*CD52*
2.40	0.0206	NM_001030288	*SPN*
2.40	0.0237	NM_001169110	*SCO2*
2.35	0.0084	NM_001030288	*SPN*
2.30	0.0001	NM_002994	*CXCL5*
2.30	0.0106	NM_033425	*DIXDC1*
2.29	0.0145	NM_001425	*EMP3*
2.28	0.0147	NM_002865	*RAB2A*
2.28	0.0113	NM_139283	*PPTC7*
2.27	0.0019	NM_001525	*HCRTR1*
2.25	0.0240	NM_017887	*C1orf123*
2.25	0.0241	NM_015879	*ST8SIA3*
2.23	0.0489	NM_002699	*POU3F1*
2.23	0.0057	NM_032451	*SPIRE2*
2.22	0.0072	NM_001110354	*ZP3*
2.20	0.0492	NM_030647	*KDM7A*
2.18	0.0309	NM_000744	*CHRNA4*
2.17	0.0311	NM_003123	*SPN*
2.15	0.0057	NM_153240	*NPHP3*

Enrichment over Day 0	T-Test	Access. Number	Gene Symbol
2.13	0.0149	NM_033554	*HLA-DPA1*
2.13	0.0251	NM_022550	*XRCC4*
2.13	0.0019	NM_001040126	*PQLC2*
2.12	0.0040	NM_020958	*PPP4R4*
2.12	0.0137	NM_005603	*ATP8B1*
2.11	0.0293	NM_001229	*CASP9*
2.08	0.0052	NM_001136030	*TESPA1*
2.07	0.0084	NM_006412	*AGPAT2*
2.07	0.0035	NM_001135057	*LRRC15*
2.05	0.0001	NM_006499	*LGALS8*
2.05	0.0006	NM_145887	*PIDD1*
2.03	0.0275	NM_000391	*TPP1*
2.02	0.0178	NM_001206736	*NSFL1C*
2.00	0.0290	NM_197962	*GLRX2*
2.00	0.0463	NM_020992	*PDLIM1*
1.99	0.0066	NM_138433	*KLHDC7B*
1.97	0.0181	NM_001009877	*BRD9*
1.97	0.0044	NM_080622	*ABHD16B*
1.97	0.0220	NM_032693	*NAA11*
1.96	0.0338	NM_001009568	*SMPDL3B*
1.96	0.0369	NM_001256302	*ETF1*
1.94	0.0085	NM_001204862	*TCEB1*
1.94	0.0068	NM_001278116	*L1CAM*
1.91	0.0354	NM_001111125	*IQSEC2*
1.91	0.0009	NM_007023	*RAPGEF4*
1.91	0.0150	NM_007215	*POLG2*
1.91	0.0164	NM_001037171	*ACOT9*
1.91	0.0003	NM_015247	*CYLD*
1.91	0.0233	NM_002698	*POU2F2*
1.91	0.0097	NM_001137560	*TMEM151B*
1.90	0.0339	NM_001270616	*PROX1*
1.90	0.0185	NM_001198656	*AKAP2*
1.89	0.0009	NM_032878	*ALKBH6*
1.89	0.0036	NM_194250	*ZNF804A*
1.88	0.0012	NM_001012991	*KNOP1*
1.87	0.0202	NM_001076683	*UBTF*
1.87	0.0025	NM_032511	*FAXC*

Enrichment over Day 0	T-Test	Access. Number	Gene Symbol
1.87	0.0395	NM_020979	*SH2B2*
1.86	0.0091	NM_206827	*RASL11A*
1.86	0.0143	NM_001001330	*REEP3*
1.86	0.0219	NM_001282352	*ADAMTS10*
1.86	0.0039	NM_173598	*KSR2*
1.85	0.0421	NM_001042410	*ANKZF1*
1.85	0.0153	NM_002630	*PGC*
1.84	0.0270	NM_201550	*LRRC10*
1.84	0.0182	NM_000651	*CR1*
1.84	0.0033	NM_058244	*WNT8A*
1.84	0.0033	NM_000641	*IL11*
1.84	0.0287	NM_018026	*PACS1*
1.84	0.0031	NM_001256915	*IDNK*
1.83	0.0200	NM_013272	*SLCO3A1*
1.83	0.0067	NM_005224	*ARID3A*
1.83	0.0441	NM_001127196	*CNBP*
1.83	0.0089	NM_001102654	*NTF3*
1.82	0.0000	NM_000417	*IL2RA*
1.82	0.0106	NM_001127216	*GFI1*
1.82	0.0051	NM_004333	*BRAF*
1.82	0.0011	NM_001204353	*SPOCK3*
1.81	0.0305	NM_003857	*GALR2*
1.81	0.0004	NM_001267548	*ARFRP1*
1.80	0.0306	NM_001009894	*C12orf29*
1.80	0.0092	NM_145168	*SDR42E1*
1.79	0.0258	NM_080391	*PTP4A2*
1.79	0.0368	NM_022659	*EBF2*
1.79	0.0011	NM_020452	*ATP8B2*
1.78	0.0145	NM_016548	*GOLM1*
1.78	0.0000	NM_145044	*ZNF501*
1.78	0.0075	NM_182488	*USP12*
1.78	0.0396	NM_001012279	*SOGA3*
1.78	0.0034	NM_015140	*TTLL12*
1.78	0.0496	NM_000121	*EPOR*
1.77	0.0267	NM_177543	*PLPP2*
1.77	0.0008	NM_018416	*FOXJ2*
1.77	0.0261	NM_001142534	*SH2D3C*

Enrichment over Day 0	T-Test	Access. Number	Gene Symbol
1.77	0.0294	NM_001193301	*SEMA4A*
1.76	0.0126	NM_032107	*L3MBTL1*
1.76	0.0047	NM_017882	*CLN6*
1.76	0.0036	NM_001201429	*CABIN1*
1.76	0.0001	NM_014290	*TDRD7*
1.76	0.0159	NM_004564	*GATB*
1.76	0.0487	NM_194454	*KRIT1*
1.76	0.0051	NM_001407	*CELSR3*
1.75	0.0081	NM_133640	*MED22*
1.74	0.0036	NM_018026	*PACS1*
1.74	0.0001	NM_001110822	*TDRD12*
1.74	0.0000	NM_016937	*POLA1*
1.74	0.0430	NM_000913	*OPRL1*
1.74	0.0275	NM_001257392	*CD63*
1.74	0.0010	NM_006984	*CLDN10*
1.73	0.0053	NM_001042474	*ZNF565*
1.72	0.0055	NM_207661	*ZC3H14*
1.72	0.0151	NM_005113	*GOLGA5*
1.72	0.0008	NM_001258346	*PACRGL*
1.72	0.0058	NM_002488	*NDUFA2*
1.72	0.0127	NM_033301	*RPL8*
1.72	0.0073	NM_138412	*RDH13*
1.72	0.0004	NM_001122772	*AGAP2*
1.71	0.0012	NM_001048195	*RCC1*
1.71	0.0006	NM_178836	*PLD6*
1.71	0.0133	NM_013305	*ST8SIA5*
1.71	0.0462	NM_000363	*TNNI3*
1.71	0.0001	NM_001135655	*LY6H*
1.71	0.0127	NM_003701	*TNFSF11*
1.71	0.0098	NM_001099409	*EHBP1L1*
1.70	0.0073	NM_133459	*CCBE1*
1.70	0.0047	NM_000537	*REN*
1.70	0.0000	NM_001170779	*FAM122C*
1.70	0.0384	NM_006547	*IGF2BP3*
1.70	0.0277	NM_024122	*APOO*
1.70	0.0495	NM_001198832	*PDE4DIP*
1.70	0.0012	NM_014347	*ZNF324*

Enrichment over Day 0	T-Test	Access. Number	Gene Symbol
1.69	0.0311	NM_012401	*PLXNB2*
1.69	0.0140	NM_145644	*MRPL35*
1.69	0.0110	NM_001144883	*PRICKLE1*
1.69	0.0290	NM_016354	*SLCO4A1*
1.69	0.0104	NM_014800	*ELMO1*
1.69	0.0164	NM_014161	*MRPL18*
1.69	0.0068	NM_001007527	*LMBRD2*
1.69	0.0228	NM_001282652	*STIP1*
1.69	0.0028	NM_014079	*KLF15*
1.69	0.0019	NM_199047	*TBPL2*
1.69	0.0105	NM_147134	*NFX1*
1.69	0.0004	NM_031439	*SOX7*
1.68	0.0062	NM_001134775	*KLC2*
1.68	0.0264	NM_033518	*SLC38A5*
1.68	0.0230	NM_005263	*GFI1*
1.68	0.0022	NM_001145204	*SHISA9*
1.68	0.0086	NM_004075	*CRY1*
1.68	0.0072	NM_001127183	*CFLAR*
1.68	0.0224	NM_014298	*QPRT*
1.68	0.0175	NM_006278	*ST3GAL4*
1.68	0.0028	NM_016562	*TLR7*
1.68	0.0053	NM_016086	*STYXL1*
1.67	0.0024	NM_001160302	*SYNJ1*
1.67	0.0025	NM_032875	*FBXL20*
1.67	0.0148	NM_030810	*TXNDC5*
1.67	0.0056	NM_005828	*DCAF7*
1.67	0.0179	NM_032421	*CLIP2*
1.67	0.0031	NM_003044	*SLC6A12*
1.67	0.0221	NM_080622	*ABHD16B*
1.67	0.0375	NM_000685	*AGTR1*
1.67	0.0016	NM_001286754	*SYNPO2*
1.67	0.0183	NM_001290061	*SEMA3B*
1.67	0.0028	NM_213662	*STAT3*
1.66	0.0260	NM_004789	*LHX2*
1.66	0.0091	NM_001940	*ATN1*
1.66	0.0057	NM_017955	*CDCA4*
1.66	0.0067	NM_017613	*DONSON*

Enrichment over Day 0	T-Test	Access. Number	Gene Symbol
1.66	0.0067	NM_001166105	*TADA2A*
1.66	0.0354	NM_001253798	*ZNF331*
1.66	0.0226	NM_001145648	*RASGRF1*
1.66	0.0323	NM_000161	*GCH1*
1.65	0.0264	NM_014938	*MLXIP*
1.65	0.0080	NM_020375	*TIGAR*
1.65	0.0101	NM_001207008	*AFDN*
1.65	0.0008	NM_001965	*EGR4*
1.65	0.0034	NM_005188	*CBL*
1.65	0.0193	NM_001278613	*PCDH1*
1.65	0.0267	NM_002235	*KCNA6*
1.65	0.0011	NM_014053	*FLVCR1*
1.65	0.0026	NM_003022	*SH3BGRL*
1.65	0.0186	NM_001002258	*ATP5G3*
1.65	0.0005	NM_002214	*ITGB8*
1.64	0.0311	NM_001168393	*CREBRF*
1.64	0.0024	NM_002027	*FNTA*
1.64	0.0502	NM_001276469	*B4GALNT1*
1.64	0.0008	NM_017675	*CDHR2*
1.64	0.0258	NM_001080524	*C16orf90*
1.64	0.0065	NM_001171088	*CLCN2*
1.64	0.0029	NM_006311	*NCOR1*
1.64	0.0016	NM_025150	*TARS2*
1.64	0.0069	NM_001113381	*RGS4*
1.64	0.0200	NM_017881	*NMRK1*
1.63	0.0150	NM_130901	*OTUD7A*
1.63	0.0150	NM_001177802	*RANGRF*
1.63	0.0004	NM_015221	*DNMBP*
1.63	0.0043	NM_003766	*BECN1*
1.63	0.0114	NM_019087	*ARL15*
1.63	0.0044	NM_005917	*MDH1*
1.63	0.0006	NM_024531	*SLC52A2*
1.63	0.0035	NM_001270661	*MARCH6*
1.62	0.0283	NM_019892	*INPP5E*
1.62	0.0126	NM_001017915	*INPP5D*
1.62	0.0195	NM_001031628	*SMAGP*
1.62	0.0081	NM_001039877	*STRN4*

Enrichment over Day 0	T-Test	Access. Number	Gene Symbol
1.62	0.0035	NM_014235	*UBL4A*
1.62	0.0324	NM_145912	*NFAM1*
1.62	0.0011	NM_001243646	*CD2BP2*
1.62	0.0136	NM_015985	*ANGPT4*
1.62	0.0068	NM_001159531	*BEGAIN*
1.62	0.0016	NM_006828	*ASCC3*
1.62	0.0116	NM_153213	*ARHGEF19*
1.62	0.0347	NM_030818	*CCDC130*
1.62	0.0179	NM_001201407	*ZNF778*
1.61	0.0032	NM_001004754	*OR51I2*
1.61	0.0008	NM_001144769	*DST*
1.61	0.0049	NM_002916	*RFC4*
1.61	0.0149	NM_001127892	*SALL1*
1.61	0.0222	NM_001287397	*C6orf1*
1.61	0.0005	NM_001256653	*ZNF43*
1.61	0.0055	NM_080862	*SPSB4*
1.61	0.0250	NM_001040100	*SPTSSB*
1.61	0.0031	NM_001244871	*DAB2*
1.61	0.0222	NM_024805	*RBFA*
1.61	0.0231	NM_001099294	*KIAA1644*
1.61	0.0127	NM_006662	*SRCAP*
1.61	0.0008	NM_032889	*MFSD5*
1.60	0.0020	NM_052886	*MAL2*
1.60	0.0040	NM_024665	*TBL1XR1*
1.60	0.0116	NM_002121	*HLA-DPB1*
1.60	0.0016	NM_020163	*SEMA3G*
1.60	0.0134	NM_031421	*TTC25*
1.60	0.0009	NM_024958	*NRSN2*
1.60	0.0224	NM_001452	*FOXF2*
1.60	0.0318	NM_001033953	*CALCA*
1.60	0.0083	NM_018689	*CEMIP*
1.60	0.0477	NM_014236	*GNPAT*
1.60	0.0018	NM_015131	*WDR43*
1.60	0.0168	NM_001005185	*OR6N1*
1.60	0.0152	NM_020182	*PMEPA1*
1.60	0.0378	NM_153324	*DEFB123*
1.60	0.0049	NM_001267698	*CD63*

Enrichment over Day 0	T-Test	Access. Number	Gene Symbol
1.60	0.0031	NM_002332	*LRP1*
1.60	0.0077	NM_014078	*MRPL13*
1.60	0.0093	NM_020637	*FGF22*
1.59	0.0025	NM_020436	*SALL4*
1.59	0.0301	NM_015912	*FAM135B*
1.59	0.0012	NM_144627	*TSACC*
1.59	0.0403	NM_001257096	*PAX1*
1.59	0.0000	NM_004207	*SLC16A3*
1.59	0.0155	NM_003965	*CCRL2*
1.59	0.0289	NM_021960	*MCL1*
1.59	0.0325	NM_001769	*CD9*
1.59	0.0059	NM_016175	*C5orf45*
1.59	0.0033	NM_016354	*SLCO4A1*
1.59	0.0094	NM_002476	*MYL4*
1.59	0.0036	NM_018132	*CENPQ*
1.59	0.0047	NM_016261	*TUBD1*
1.59	0.0465	NM_152448	*TERB2*
1.58	0.0020	NM_001098794	*FAM160A2*
1.58	0.0048	NM_021630	*PDLIM2*
1.58	0.0104	NM_183380	*DST*
1.58	0.0031	NM_001243254	*DENND5A*
1.58	0.0092	NM_020726	*NLN*
1.58	0.0266	NM_001040715	*KIAA0895L*
1.58	0.0066	NM_207510	*LCNL1*
1.58	0.0317	NM_001122770	*ZBTB37*
1.58	0.0450	NM_005391	*PDK3*
1.58	0.0072	NM_020753	*CASKIN2*
1.58	0.0091	NM_007068	*DMC1*
1.58	0.0032	NM_025261	*LY6G6C*
1.58	0.0047	NM_032356	*NAA38*
1.58	0.0003	NM_005285	*NPBWR1*
1.58	0.0214	NM_005304	*FFAR3*
1.58	0.0145	NM_001190826	*FAM217B*
1.58	0.0007	NM_153228	*ANKFN1*
1.58	0.0140	NM_130775	*XAGE5*
1.58	0.0003	NM_024831	*TGS1*
1.58	0.0000	NM_014586	*HUNK*

Enrichment over Day 0	T-Test	Access. Number	Gene Symbol
1.58	0.0012	NM_032042	*FAM172A*
1.57	0.0396	NM_001128165	*FBLN7*
1.57	0.0006	NM_001289021	*KIF6*
1.57	0.0234	NM_001001479	*SLC35E4*
1.57	0.0057	NM_017877	*SLC35F6*
1.57	0.0446	NM_015364	*LY96*
1.57	0.0181	NM_020404	*CD248*
1.57	0.0402	NM_025069	*ZNF703*
1.57	0.0094	NM_003619	*PRSS12*
1.57	0.0003	NM_001198695	*MFAP4*
1.57	0.0326	NM_130832	*OPA1*
1.57	0.0140	NM_032431	*SYVN1*
1.57	0.0130	NM_018110	*DOK4*
1.57	0.0086	NM_024717	*MCTP1*
1.57	0.0081	NM_015264	*KIAA0930*
1.57	0.0027	NM_001286582	*PHRF1*
1.57	0.0007	NM_025004	*CCDC15*
1.57	0.0007	NM_145060	*SKA1*
1.57	0.0018	NM_020870	*SH3RF1*
1.57	0.0011	NM_024337	*IRX1*
1.57	0.0004	NM_017723	*TOR4A*
1.57	0.0169	NM_003120	*SPI1*
1.57	0.0032	NM_001197294	*DPYSL3*
1.56	0.0452	NM_207391	*RGS9BP*
1.56	0.0034	NM_001038	*SCNN1A*
1.56	0.0005	NM_001168364	*KRTCAP3*
1.56	0.0027	NM_016026	*RDH11*
1.56	0.0137	NM_022351	*NECAB1*
1.56	0.0009	NM_032876	*AJUBA*
1.56	0.0143	NM_000056	*BCKDHB*
1.56	0.0247	NM_152470	*RNF165*
1.56	0.0105	NM_001037162	*ACOT6*
1.56	0.0138	NM_015960	*CUTC*
1.56	0.0213	NM_001135095	*FNDC3B*
1.56	0.0088	NM_006829	*ADIRF*
1.55	0.0041	NM_183419	*RNF19A*
1.55	0.0412	NM_198508	*KLRG2*

Enrichment over Day 0	T-Test	Access. Number	Gene Symbol
1.55	0.0002	NM_032871	*RELT*
1.55	0.0001	NM_005116	*SLC23A2*
1.55	0.0259	NM_015169	*RRS1*
1.55	0.0017	NM_021241	*WIZ*
1.55	0.0452	NM_003000	*SDHB*
1.55	0.0042	NM_001243942	*PRPSAP2*
1.55	0.0456	NM_203401	*STMN1*
1.55	0.0117	NM_003033	*ST3GAL1*
1.55	0.0150	NM_001029880	*SFMBT2*
1.55	0.0205	NM_138375	*CABLES1*
1.55	0.0026	NM_020211	*RGMA*
1.55	0.0208	NM_006540	*NCOA2*
1.55	0.0026	NM_173499	*SPATA8*
1.55	0.0122	NM_001204126	*LRMP*
1.55	0.0035	NM_020384	*CLDN2*
1.55	0.0071	NM_019004	*ANKIB1*
1.55	0.0224	NM_000868	*HTR2C*
1.54	0.0043	NM_002473	*MYH9*
1.54	0.0246	NM_001113349	*ECE1*
1.54	0.0118	NM_000117	*EMD*
1.54	0.0142	NM_017791	*FLVCR2*
1.54	0.0052	NM_000369	*TSHR*
1.54	0.0262	NM_001127896	*CHST8*
1.54	0.0001	NM_005806	*OLIG2*
1.54	0.0061	NM_014478	*CRCP*
1.54	0.0020	NM_001193269	*EML2*
1.54	0.0160	NM_001145365	*ZNF652*
1.54	0.0071	NM_024009	*GJB3*
1.54	0.0010	NM_001258214	*IL12RB2*
1.54	0.0347	NM_001113203	*NACA*
1.54	0.0243	NM_001144758	*PHLDB1*
1.54	0.0023	NM_024855	*ACTR5*
1.53	0.0280	NM_017848	*FAM120C*
1.53	0.0064	NM_022127	*SLC28A3*
1.53	0.0209	NM_001243254	*DENND5A*
1.53	0.0107	NM_025132	*WDR19*
1.53	0.0001	NM_032088	*PCDHGA8*

Enrichment over Day 0	T-Test	Access. Number	Gene Symbol
1.53	0.0380	NM_004380	*CREBBP*
1.53	0.0011	NM_000447	*PSEN2*
1.53	0.0120	NM_018602	*DNAJA4*
1.53	0.0033	NM_005723	*TSPAN5*
1.53	0.0215	NM_002155	*HSPA6*
1.53	0.0432	NM_017970	*NRDE2*
1.53	0.0324	NM_152413	*GOT1L1*
1.53	0.0024	NM_021117	*CRY2*
1.53	0.0061	NM_001142481	*NREP*
1.53	0.0006	NM_002600	*PDE4B*
1.53	0.0106	NM_000863	*HTR1B*
1.53	0.0055	NM_175902	*OGFOD3*
1.53	0.0077	NM_003914	*CCNA1*
1.53	0.0005	NM_020242	*KIF15*
1.53	0.0005	NM_001032367	*SPINT1*
1.53	0.0085	NM_015237	*KIAA1107*
1.53	0.0109	NM_033258	*GNG8*
1.53	0.0088	NM_001146335	*SLC6A15*
1.53	0.0197	NM_181700	*PPP2R1B*
1.53	0.0280	NM_001284269	*EFCAB11*
1.53	0.0010	NM_001007101	*ZNF484*
1.53	0.0481	NM_001165973	*NRG3*
1.53	0.0095	NM_173650	*DNAJC5G*
1.52	0.0435	NM_014218	*KIR2DL1*
1.52	0.0383	NM_018715	*RCC2*
1.52	0.0006	NM_001195602	*SEPHS1*
1.52	0.0012	NM_001195386	*TMEM99*
1.52	0.0174	NM_001032221	*STXBP1*
1.52	0.0179	NM_019595	*ITSN2*
1.52	0.0116	NM_001202431	*PRDX1*
1.52	0.0110	NM_005604	*POU3F2*
1.52	0.0051	NM_017838	*NHP2*
1.52	0.0013	NM_015575	*GIGYF2*
1.52	0.0054	NM_207401	*C1orf229*
1.52	0.0170	NM_005864	*EFS*
1.52	0.0075	NM_000377	*WAS*
1.52	0.0114	NM_002289	*LALBA*

Enrichment over Day 0	T-Test	Access. Number	Gene Symbol
1.52	0.0010	NM_022456	*RAB3IP*
1.52	0.0069	NM_001685	*ATP5J*
1.52	0.0242	NM_001282934	*PRDM15*
1.52	0.0232	NM_001286693	*RGS22*
1.52	0.0409	NM_001031738	*TMEM150A*
1.52	0.0335	NM_001006939	*LRRC18*
1.52	0.0089	NM_001014380	*KATNAL1*
1.52	0.0028	NM_207581	*DUOXA2*
1.52	0.0016	NM_001040633	*PRKAG2*
1.52	0.0013	NM_201569	*SMG7*
1.52	0.0041	NM_001136019	*FCGRT*
1.52	0.0015	NM_012425	*RSU1*
1.52	0.0021	NM_024003	*L1CAM*
1.52	0.0034	NM_018210	*NAXD*
1.52	0.0148	NM_001166301	*DHX40*
1.52	0.0461	NM_002227	*JAK1*
1.52	0.0020	NM_005920	*MEF2D*
1.52	0.0006	NM_001142964	*C22orf46*
1.52	0.0050	NM_032446	*MEGF10*
1.52	0.0039	NM_152263	*TPM3*
1.51	0.0185	NM_001142645	*NEMP2*
1.51	0.0066	NM_001282690	*SECISBP2*
1.51	0.0327	NM_003248	*THBS4*
1.51	0.0006	NM_013339	*ALG6*
1.51	0.0078	NM_024707	*GEMIN7*
1.51	0.0171	NM_016045	*PRELID3B*
1.51	0.0030	NM_001277163	*CEACAM3*
1.51	0.0002	NM_020959	*ANO8*
1.51	0.0133	NM_001031700	*FAM198B*
1.51	0.0100	NM_003500	*ACOX2*
1.51	0.0210	NM_018155	*SLC25A36*
1.51	0.0062	NM_198581	*ZC3H6*
1.51	0.0003	NM_001134707	*SARDH*
1.51	0.0209	NM_001166449	*ITIH4*
1.51	0.0009	NM_133368	*RSPRY1*
1.51	0.0123	NM_006559	*KHDRBS1*
1.51	0.0291	NM_173553	*TRIML2*

Enrichment over Day 0	T-Test	Access. Number	Gene Symbol
1.51	0.0014	NM_004286	*GTPBP1*
1.51	0.0422	NM_014167	*CCDC59*
1.51	0.0088	NM_144697	*CIART*
1.51	0.0235	NM_001079530	*CFC1B*
1.51	0.0190	NM_152634	*TCEANC*
1.50	0.0105	NM_177925	*H2AFJ*
1.50	0.0017	NM_001159770	*SLC39A11*
1.50	0.0213	NM_004465	*FGF10*
1.50	0.0080	NM_138334	*JOSD2*
1.50	0.0014	NM_001080556	*CFAP52*
1.50	0.0030	NM_015629	*PRPF31*
1.50	0.0108	NM_001146213	*TBC1D15*
1.50	0.0290	NM_130795	*RGS3*
1.50	0.0016	NM_152654	*DAND5*
1.50	0.0004	NM_000390	*CHM*
1.50	0.0021	NM_017822	*KANSL2*
1.50	0.0030	NM_001040630	*NCALD*
1.50	0.0296	NM_145719	*TIGD3*
1.50	0.0008	NM_004667	*HERC2*
1.50	0.0149	NM_014698	*TMEM63A*
1.50	0.0027	NM_175063	*EMC10*
1.50	0.0435	NM_018322	*SAYSD1*
1.50	0.0006	NM_007174	*CIT*
1.50	0.0033	NM_001163809	*WDR81*
1.50	0.0014	NM_022167	*XYLT2*
1.50	0.0114	NM_133433	*NIPBL*
1.50	0.0388	NM_031953	*SNX25*
1.50	0.0005	NM_002492	*NDUFB5*
1.50	0.0050	NM_016309	*LCMT1*
1.50	0.0180	NM_198156	*VHL*
1.50	0.0192	NM_002198	*IRF1*
1.50	0.0002	NM_003129	*SQLE*
1.50	0.0053	NM_000097	*CPOX*
1.50	0.0395	NM_001278116	*L1CAM*
1.50	0.0122	NM_001146288	*AIFM3*
1.50	0.0007	NM_201647	*STAMBP*
1.50	0.0021	NM_174905	*FAM98C*

Enrichment over Day 0	T-Test	Access. Number	Gene Symbol
1.50	0.0080	NM_001243259	*HINFP*
1.50	0.0401	NM_004254	*SLC22A8*
1.50	0.0005	NM_001190447	*PPP2R3A*
1.50	0.0018	NM_014671	*UBE3C*
1.50	0.0089	NM_022044	*SDF2L1*
1.50	0.0084	NM_003947	*KALRN*
1.50	0.0050	NM_000554	*CRX*
1.50	0.0488	NM_001135776	*ZBTB43*
1.50	0.0005	NM_024501	*HOXD1*
1.50	0.0072	NM_001619	*GRK2*
1.50	0.0042	NM_007186	*CEP250*
1.50	0.0008	NM_005570	*LMAN1*
1.50	0.0132	NM_020143	*PNO1*
